# Long non-coding RNA Gm15441 attenuates hepatic inflammasome activation in response to PPARA agonism and fasting

**DOI:** 10.1038/s41467-020-19554-7

**Published:** 2020-11-17

**Authors:** Chad N. Brocker, Donghwan Kim, Tisha Melia, Kritika Karri, Thomas J. Velenosi, Shogo Takahashi, Daisuke Aibara, Jessica A. Bonzo, Moshe Levi, David J. Waxman, Frank J. Gonzalez

**Affiliations:** 1 Laboratory of Metabolism, Center for Cancer Research, National Cancer Institute, National Institutes of Health, Bethesda, MD 20814 USA; 2 Department of Biology and Bioinformatics Program, Boston University, Boston, MA 02215 USA; 3 Biochemistry and Molecular & Cellular Biology, Georgetown University, Washington, DC 20057 USA

**Keywords:** Biochemistry, Long non-coding RNAs

## Abstract

Exploring the molecular mechanisms that prevent inflammation during caloric restriction may yield promising therapeutic targets. During fasting, activation of the nuclear receptor peroxisome proliferator-activated receptor α (PPARα) promotes the utilization of lipids as an energy source. Herein, we show that ligand activation of PPARα directly upregulates the long non-coding RNA gene *Gm15441* through PPARα binding sites within its promoter. *Gm15441* expression suppresses its antisense transcript, encoding thioredoxin interacting protein (TXNIP). This, in turn, decreases TXNIP-stimulated NLR family pyrin domain containing 3 (NLRP3) inflammasome activation, caspase-1 (CASP1) cleavage, and proinflammatory interleukin 1β (IL1B) maturation. *Gm15441*-null mice were developed and shown to be more susceptible to NLRP3 inflammasome activation and to exhibit elevated CASP1 and IL1B cleavage in response to PPARα agonism and fasting. These findings provide evidence for a mechanism by which PPARα attenuates hepatic inflammasome activation in response to metabolic stress through induction of lncRNA *Gm15441*.

## Abstract (web summary)

PPAR-alpha is a ligand responsive transcription factor that mediates energy metabolism during fasting in the liver. Here the authors show that Gm15441 is a PPAR-alpha dependent lncRNA that prevents the expression of its antisense transcript, thioredoxin interacting protein (TXNIP), and attenuates inflammasome activation.

## Introduction

There is growing support for a strong link between metabolism and inflammation^1,2^. Various fasting regimes are known to provide many health benefits, including anti-inflammatory effects^3^. Peroxisome proliferator-activated receptor α (PPARα) is a ligand-activated nuclear receptor and transcription factor that is a key regulator of fatty acid oxidation and the fasting response. PPARα facilitates metabolic remodeling that promotes lipid oxidation, and its dysregulation contributes to metabolic disorders and liver disease^4^. Prolonged activation by the PPARα-specific ligand WY-14643 has many phenotypic characteristics that parallel the fasting response^5–8^. Metabolic changes that promote fat oxidation can lead to metabolic stress through increased lipid peroxidation and reactive oxygen species generation, both of which are known pro-inflammatory signals^9,10^. Interestingly in the context of elevated fatty acid oxidation, several synthetic PPARα agonists, including WY-14643, act as potent anti-inflammatory agents^11^. However, the regulatory mechanisms underlying how PPARα prevents inflammation are not well understood.

Long non-coding RNAs (lncRNAs) act as regulators of gene expression and play important regulatory roles in many metabolic processes. For example, lncRNA Blnc1 works in concert with another important hepatic nuclear receptor, liver X receptor (LXR), to activate the lipogenic gene program^12^. Downregulation of lncRNA lncOb reduces leptin, leading to a leptin responsive form of obesity^13^. Other studies found that lncRNAs are regulated during adipocyte differentiation^14,15^, are expressed in the liver in a sex-dependent manner when regulated by growth hormone^16,17^, and can be strongly induced by xenobiotic exposure^18^. A compartment-specific transcriptional profiling approach revealed that lncRNA PAXIP1-AS1 regulates pulmonary arterial hypertension by modulating smooth muscle cell function^19^. Further, lncRNA HOTAIR influences glucose metabolism by upregulation of GLUT1 in hepatocellular carcinoma cells^20^. It is, therefore, reasonable to consider that lncRNAs may play important roles in the metabolic remodeling and anti-inflammatory actions that occur after PPARα activation.

Thioredoxin interacting protein (TXNIP) acts as a critical relay linking oxidative and endoplasmic reticulum (ER) stress to inflammation through NLR family pyrin domain containing 3 (NLRP3) inflammasome activation^21^. NLRP3 inflammasome activation is a two-step process. The first step involves inflammasome assembly and is referred to as “priming”. At this stage the inflammasome is waiting to be “activated” by diverse stimuli including ionic flux, lysosomal damage, mitochondrial dysfunction, and, notably, oxidative stress^22–24^. These signals then trigger inflammasome activation resulting in caspase cleavage and cleavage of target proteins such as pro-inflammatory cytokines. TXNIP is bound intracellularly to thioredoxin and plays a role in oxidative stress-induced NLRP3 inflammasome activation^25^. As oxidative stress increases, thioredoxin is released from TXNIP freeing it to associate with the NLRP3 inflammasome. Several studies have shown that elevated TXNIP expression impacts NLRP3 inflammasome activation and cleavage of caspase-1 (CASP1) to the mature, proteolytically active p20 form^21,26,27^. Mature CASP1 (p20) then cleaves interleukin 1β (IL1B) precursor to the active pro-inflammatory cytokine. Studies have also indicated that NLRP3 inflammasome activity is attenuated by mono- and polyunsaturated fatty acids, which are endogenous PPARα agonists^28,29^. RNA-seq data presented here revealed that lncRNA *Gm15441*, an antisense lncRNA of *Txnip*, is robustly increased by PPARα activation. The primary *Gm15441* transcript is spliced, generating a mature RNA composed of four exons. Two of the four exons directly overlap with exonic regions of the *Txnip* gene. Antisense transcripts often interact the corresponding sense transcripts encoded from the opposing gene, thereby interfering with post-transcriptional control by promoting transcript degradation or disrupting translation^30^. The *Gm15441* transcript was identified through several independent transcriptome profiling studies providing support that this may be a functionally important lncRNA^31–33^. Using siRNA, one study found that Gm15441 knockdown by siRNA impacted hepatocyte fatty acid oxidation in vitro, however, the mechanism was not determined^31^.

In the current study, the mechanisms by which the PPARα target lncRNA *Gm15441* prevents inflammation during periods of metabolic stress were investigated. ChIP-seq datasets were analyzed for PPARα binding sites within the *Gm15441* promoter, and *Gm15441* regulatory elements were confirmed using reporter gene assays and PPARα ChIP studies. *Gm15441* transgene expression downregulated *Txnip*, demonstrating lncRNA-mediated gene suppression in vitro. CRIPSR/Cas9-mediated gene editing was employed to develop a *Gm15441* knockout (*Gm15441*^LSL^) mouse model. Basal TXNIP levels in *Gm15441*^LSL^ mice were significantly elevated over wild-type mice, supporting the role of *Gm15441* as a negative regulator of *Txnip* expression in vivo. *Gm15441*^LSL^ mice were treated with a PPARα agonist or fasted to assess how loss of *Gm15441* impacts hepatic inflammasome activation in response to both pharmacological and physiologically-induced metabolic stress. TXNIP protein, active cleaved CASP1 levels, and IL1B cleavage were elevated in *Gm15441*^LSL^ mice, and were further increased by PPARα activation, indicating that this lncRNA plays a major role in attenuating inflammasome activation. Thus, hepatic PPARα directly regulates the lncRNA *Gm15441*, which in turn suppresses *Txnip* expression, attenuating NLRP3 inflammasome activation during periods of metabolic stress. Together, these findings support a regulatory mechanism whereby fatty acids mobilized from adipose tissue during fasting activate PPARα, which in turn suppresses the NLRP3 inflammasome by strong induction of the TXNIP-suppressing antisense lncRNA gene *Gm15441*. Thus, these studies reveal a regulatory mechanism supporting the beneficial effects of fasting, namely, reduced inflammation.

## Results

### LncRNA regulation by PPARα is highly tissue-specific

**Fig. 1:**
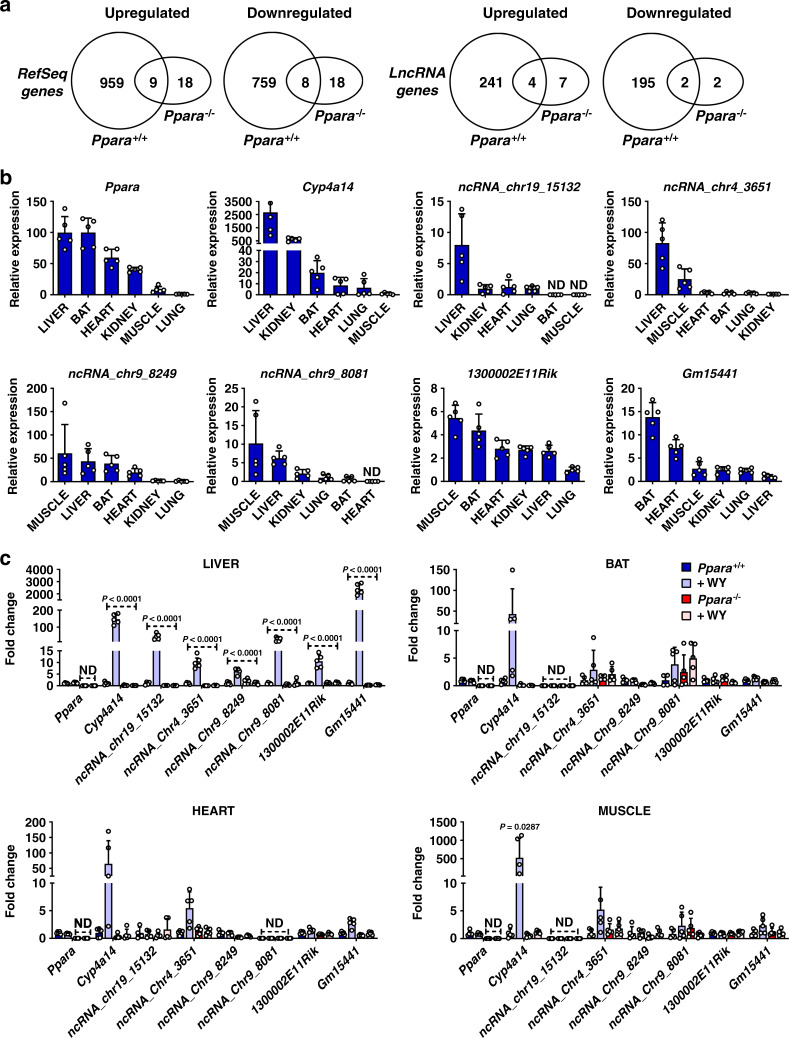
LncRNA identified as PPARα targets are expressed in several oxidative tissues but exhibit liver-specific transcriptional response to WY-14643. **a** Venn diagrams of RefSeq transcripts and lncRNA transcripts that were differentially regulated by WY-14643 (fold change > 2 at FDR < 0.05) in wild-type and in *Ppara*^−/−^ mouse liver, as determined by RNA-seq. 123 of the RefSeq genes are non-coding, indicated by their NR accession numbers. Six RefSeq genes and six lncRNA genes show opposite responses to WY-14643 treatment and are excluded from the gene counts shown. **b** Relative basal lncRNA expression in select tissues. **c** LncRNA expression in tissues from *Ppara*^+/+^ and *Ppara*^−/−^ mice treated with WY-14643 for 48 h. At least five mice were analyzed for each genotype and treatment group. Each bar represents the mean ± SD for *n* = 5 tissue samples. Adjusted *p* values, provided in the panels, as determined by ANOVA with Tukey’s multiple comparison correction, one-sided for comparisons to *Ppara*^+/+^ mice or as shown (dashed horizontal lines). ND not detected.

To assess the regulation of lncRNAs by PPARα, RNA-seq was performed using total liver RNA isolated from *Ppara*^+/+^ and *Ppara*^−/−^ mice, both with and without dietary exposure to the PPARα agonist WY-14643. A lncRNA discovery pipeline was implemented that identified 15,558 liver-expressed lncRNA genes. Of these, 13,343 were intergenic, 1966 were antisense to known coding genes, and 249 were intragenic lncRNAs. Forty-four percent of the 15,558 liver-expressed lncRNAs are considered novel^17^. Differential gene expression analysis revealed that a total of 1735 RefSeq genes and 442 liver-expressed lncRNA genes responded to treatment with WY-14643 at an expression fold-change > 2 at FDR < 0.05, with 968 RefSeq genes and 245 lncRNA genes upregulated, and 767 RefSeq genes and 197 lncRNA genes downregulated following WY-14643 treatment (Supplementary Data 1 and 2). Only 17 RefSeq genes (1.0%) and six lncRNAs (1.3%) responded to WY-14643 in the same manner in *Ppara*-null mice. Thus, ~99% of these transcriptomic responses are PPARα-dependent (Fig. 1a).

Comparison of the PPARα-responsive lncRNAs to lncRNAs responsive to agonist ligands of two other nuclear receptors in mouse liver, namely CAR and PXR^18^, identified eight lncRNAs that are induced by all three nuclear receptors and 11 lncRNAs repressed by all three receptors. Forty-five other lncRNAs were induced, or were repressed, in common by PPARα and CAR, and 30 other lncRNAs were either induced or repressed in common by PPARα and PXR (Supplementary Data 3). Overall, 94 (21%) of the 442 PPARα-responsive liver-expressed lncRNAs showed common responses to CAR and/or PXR activation. Furthermore, 59 other PPARα-responsive lncRNAs showed opposite patterns of response to PPARα as compared to CAR or PXR (Supplementary Data 3). These findings are consistent with the partial functional overlap between these three xenobiotic nuclear receptors and their gene targets^34–36^.

Pathway analysis revealed that the WY-14643 upregulated genes are most highly enriched for the following biological processes: lipid metabolism, peroxisome, DNA replication, DNA repair, cell cycle, and fatty acid oxidation (Supplementary Table 1). The downregulated genes are most highly enriched for: secreted factors, monooxygenase, immunity, serine protease inhibitor, metabolism of xenobiotics, and response to virus (Supplementary Table 2). PPARα-dependent lncRNAs identified by RNA-seq were selected and mRNA levels monitored over a 24-h period after WY-14643 treatment, revealing that lncRNA expression profiles paralleled those of known protein-coding PPARα target genes (Supplementary Fig. 1a–d). As lncRNA expression is often highly tissue specific^16,37,38^, the relative basal levels were measured for PPARα-responsive lncRNAs expressed in oxidative tissues that utilize fatty acids as an energy source and express PPARα at the highest levels. *Ppara* mRNA and its classic target gene *Cyp4a14* served as controls. Basal levels of *Cyp4a14* mRNA and intergenic lncRNAs *ncRNA_chr19_15132* and *ncRNA_chr4_3651* were highest in liver, while *ncRNA_chr9_8249, ncRNA_chr9_8081*, and *1300002E11Rik* were most highly expressed in muscle. One of the PPARα-dependent lncRNAs, *Gm15441*, was also most highly expressed in brown adipose tissue (BAT) (Fig. 1b).

To assess the impact of PPARα activation on lncRNA expression and tissue specificity, *Ppara* wild-type mice (*Ppara*^+/+^) and *Ppara*-null mice (*Ppara*^−/−^) were treated with WY-14643 for 48 h, and four tissues (liver, BAT, heart, muscle) then harvested for analysis. *Cyp4a14* mRNA was markedly induced by WY-14643 in all four tissues and in a *Ppara*-dependent manner, as expected (Fig. 1c). All six lncRNAs were induced in liver, including *Gm15441*, which exhibited >2000-fold increase in wild-type livers, more than 10-fold higher than the other lncRNAs. In contrast to the induction of *Cyp4a14* mRNA, lncRNA induction by WY-14643 was liver-specific, with no significant induction of the five other lncRNAs seen in BAT, heart, or muscle (Fig. 1c). Further, none of these lncRNAs was induced in *Ppara*^−/−^ mice in any tissue examined. Thus, PPARα-mediated induction of these lncRNAs is highly liver-specific, with *Gm15441* showing the most robust induction response.

### LncRNA *Gm15441* is antisense to *Txnip* and exhibits inverse regulation in response to PPARα activation

**Fig. 2:**
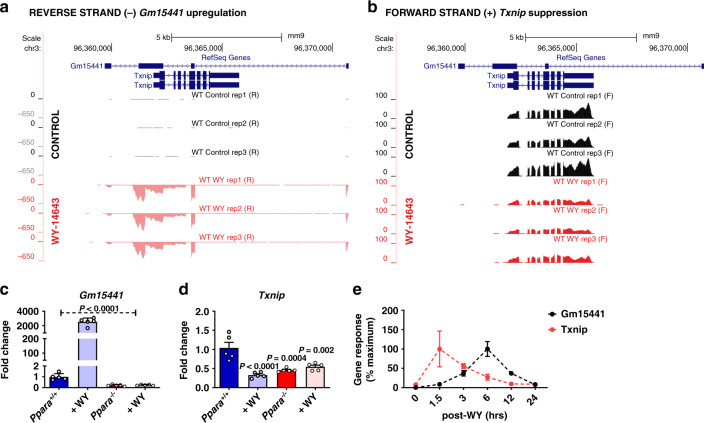
*Txnip* and lncRNA *Gm15441* are inversely regulated following PPARα activation. Wild-type (*Ppara*^+/+^) mice were treated with WY-14643 and stranded RNA-seq was performed on total liver mRNA. **a** Expression of the antisense lncRNA *Gm15441* is strongly upregulated. **b** Expression of the protein-coding *Txnip* mRNA is downregulated. Shown are the changes in expression of *Gm15441* (**c**), and *Txnip* (**d**), mRNAs in *Ppara*^+/+^ and *Ppara*^−/−^ mice treated with WY-14643, or vehicle control, for 48 h, determined by qRT-PCR. **e** Time course for changes in expression of *Gm15441* and *Txnip* mRNA over a 24 h period following treatment with WY-14643 by gavage, determined by qRT-PCR. The maximum response of *Txnip* mRNA was seen at 1.5 h and for *Gm15441* was seen at 6 h. Each data point represents the mean ± SD for *n* = 5 liver samples. Adjusted *p* values, provided in the panels, as determined by ANOVA with Tukey’s multiple comparison correction, two-sided for comparisons to *Ppara*^+/+^ mice or as shown (dashed horizontal line).

LncRNA *Gm15441* overlaps the coding region of *Txnip*, which is located on the opposing strand and responds to WY-14643 treatment, albeit with different kinetics than *Gm15441* (see below). Bioinformatic analysis confirmed that *Gm15441* lacks coding potential and was categorized as a non-coding transcript (Supplementary Fig 2a, b). Analysis of the mapped sequence reads obtained by stranded RNA-sequencing revealed a pronounced increase in expression of *Gm15441* (Fig. 2a), whereas, the *Txnip* transcript on the opposite strand was suppressed by WY-14643 at the 48-hour time point analyzed (Fig. 2b). Rapid amplification of cDNA ends (RACE) results showed that the primary hepatic *Gm15441* transcript exon-exon boundaries matched the current annotation of *Gm15441* (NR_040409.1) (Supplementary Fig. 3a and Supplementary Table 3). Gene and isoform structures analysis for mouse liver *Gm15441* by de novo transcriptome assembly using CuffMerge identified three isoforms of *Gm15441* (Supplementary Fig. 3b). The second isoform (TCONS_00330193) is identical to the RefSeq gene isoform, the first isoform is a splice variant, and the third isoform has an alternative third exon and is missing exon 4 (Supplementary Fig. 3b). All three isoforms share the precise 5′ end, two full length isoforms share a common 3′ end (Supplementary Fig. 3c, d). qRT-PCR analysis confirmed the very large, highly significant increase in *Gm15441* expression and its complete inhibition in *Ppara*^−/−^ liver (Fig. 2c). Further, *Txnip* RNA levels were significantly suppressed by WY-14643 in wild-type mice (Fig. 2d). Additionally, *Txnip* expression was attenuated in untreated *Ppara*^−/−^ compared to *Ppara*^*+/+*^ mouse liver, indicating a role for PPARα in maintaining basal expression of *Txnip* (Fig. 2d). Given that *Txnip* expression can be upregulated in human neuroblastoma cells by the PPARα activator fenofibrate, which suppresses proliferation and migration^39^, the effects of a single dose of WY-14643 on *Gm15441* and *Txnip* mRNA were examined over a 24 h period. *Txnip* mRNA was induced rapidly, with maximum expression seen at 1.5 h, followed by a marked decrease coincident with the increased expression of *Gm15441* (Fig. 2e). Inverse regulation of *Txnip* mRNA and *Gm15441* RNA were also observed in response to fasting (Supplementary Fig. 4). These time courses are markedly different from those of 14 other WY-14643-inducible protein-coding and lncRNA genes examined, where peak induction occurred 6–12 h after WY-14643 treatment (Supplementary Fig. 1), as was also found for *Gm15441*. The unusual kinetics seen with *Txnip*—early induction followed by repression coinciding with the activation of *Gm15441*—indicate that *Txnip* is a PPARα target gene whose expression in liver is inversely regulated with that of *Gm15441* after PPARα activation. Reporter assays confirmed that the *Txnip* promoter region has functional PPARα regulatory elements that overlap the sites of PPARα binding detected by ChIP-seq (Supplementary Fig. 5a, b).

### PPARα directly regulates lncRNA *Gm15441* by binding to its promotor

**Fig. 3:**
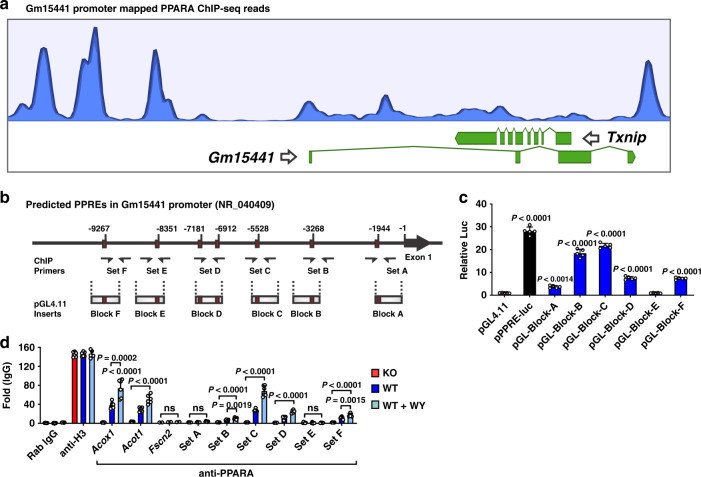
LncRNA *Gm15441* is a direct PPARα target gene. **a** PPARα ChIP-seq read peaks from agonist (GW7647)-treated mouse liver. **b** Schematic representation of seven PPRE sequences found within the *Gm15441* promoter (−10 kb). ChIP primer binding sites and reporter gene construct inserts are shown. **c** Luciferase-based reporter assays identified several functional PPREs within the *Gm15441* promoter, based on *n* = 3 replicates. **d** PPARα ChIP assays assessed PPRE binding in liver samples from *Ppara*^+/+^ and *Ppara*^−/−^ mice treated with WY-14643. Experiments were performed with at least four different livers. Rabbit IgG and antibody to histone H3 were used as negative and positive controls, respectively. Each data point represents the mean ± SD for *n* = 5 liver samples. Adjusted *p* values, provided in the panels, as determined by ANOVA with Tukey’s multiple comparison correction, one-sided for comparisons to pGL4.11 empty vector (**c**) or as indicated (**d**). ns not significant.

To identify PPARα binding sites at the *Gm15441*/*Txnip* locus, PPARα ChIP-seq datasets (GSE61817) from PPARα agonist-treated mice^40^ were analyzed (Fig. 3a). Three major ChIP-seq peaks, indicating PPARα binding, were seen upstream of *Gm15441*, and one major peak was seen in the promoter region of *Txnip*. Examination of genomic sequences upstream of *Gm15441* using Genomatix MatInspector (Genomatix, Munich, Germany) identified seven peroxisome proliferator response elements (PPREs) within six genomic regions, designated A to F, within 10 kilobase (kb) upstream of the *Gm15441* transcriptional start site (TSS) (Fig. 3b). These six *Gm15441* upstream sequences were synthesized and cloned into the pGL4.11 reporter, and luciferase assays were performed to assay the functionality of PPARα binding to the *Gm15441* promoter. A PPRE-luciferase construct containing an *Acox1* PPRE repeat was used as a positive control, and an empty pGL4.11 plasmid was used as a negative control. Luciferase activity was significantly elevated in primary mouse hepatocytes transfected with five of the six pGL4.11 constructs, consistent with direct regulation by PPARα at multiple loci (Fig. 3c). PPARα binding was also assessed by ChIP assays using a polyclonal PPARα antibody and chromatin isolated from livers of wild-type and *Ppara*^−/−^ mice fed either control diet or a diet containing WY-14643. Enrichment of PPARα binding to PPREs of known PPARα target genes, namely *Acot1* and *Acox1*, was determined by comparing binding to liver chromatin from wild-type mice fed control diet vs. WY-14643-containing diet (Fig. 3d). *Ppara*^−/−^ livers were used as a negative control to identify non-specific binding. *Fscn2* primers were used as a non-target gene promoter and negative control. Enrichment of PPARα binding was found using primer sets covering *Gm15441* regions B, C, D, and F, while no binding was seen with *Gm15441* region E (Fig. 3d), which was transcriptionally inactive (Fig. 3c). Enrichment was strongest with region C, in agreement with the luciferase reporter gene data. Enrichment at sites C and D was significantly increased by WY-14643 treatment, indicating increased PPARα recruitment at these sites. No PPARα binding was seen with chromatin from *Ppara*^−/−^ mice on the control diet. Together, these data indicate that PPARα regulates *Gm15441* transcription by direct binding of PPARα to multiple PPRE sites within 10 kb of the *Gm15441* TSS.

### Generation of a strand-specific Gm15441 knockout mouse

**Fig. 4:**
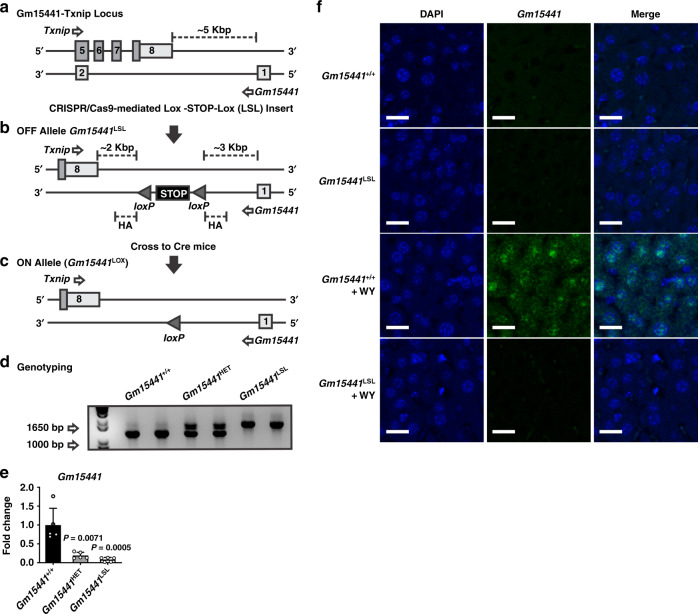
Generation of the *Gm15441*-null mouse line. Targeting strategy for generating a strand-specific *Gm15441*-null mouse line. **a** Exon structure of the targeted *Gm15441*-*Txnip* locus. **b** CRISPR/Cas9-mediated insertion of Lox-STOP-Lox (LSL) cassette selectively ablates *Gm15441* expression. **c** Cre-mediated removal of STOP cassette rescues *Gm15441* expression. **d** *Gm15441* knockout mouse genotyping. **e** Analysis of *Gm15441* RNA by qRT-PCR from livers of *Gm15441*^+/+^, *Gm15441*^+/−^, and *Gm15441*^LSL^ mice. Each data point represents the mean ± SD for *n* = 5 liver samples. Adjusted *p* values, provided in the panels, as determined by ANOVA with Tukey’s multiple comparison correction, one-sided for comparisons to *Gm15441*^+/+^ mice. **f** Representative fluorescence in situ hybridization staining of Gm15441 RNA in livers of *Gm15441*^+/+^ and *Gm15441*^LSL^ mice treated with WY-14643 for 48 h (*n* = 5, 3 images/mouse). Scale bars represents 20 nm (×200).

A gene targeting strategy was developed to generate a *Gm15441* knockout mouse without impacting *Txnip* expression on the opposing strand. To accomplish this goal, CRISPR/Cas9 was used to insert a Lox-STOP-Lox (LSL) cassette downstream of *Gm15441* exon 1 (Fig. 4a, b). The LSL cassette prevents transcription of *Gm15441* in a *Gm15441*-floxed mouse but has no effect on transcription of *Txnip*, which is inserted >2 kb downstream of the last exon of *Txnip*. Crossing with a transgenic *Cre* mouse deletes the LSL cassette and restores *Gm15441* expression (Fig. 4c). All mouse lines were on a pure C57BL/6J background. The genotyping scheme generates an approximately 1200 bp band in *Gm15441* wild-type mice (*Gm15441*^+/+^) and a 1700 bp band in *Gm15441*^LSL^ mice (Fig. 4d). Basal expression of *Gm15441* was neglectable in livers of *Gm15441*^LSL^ when compared to *Gm15441*^+/+^ mice (Fig. 4e). Expression in heterozygous *Gm15441*^HET^ mice was comparable to *Gm15441*^LSL^. To verify the subcellular localization and loss of expression of *Gm15441*, fluorescence in situ hybridization (FISH) staining was performed on livers from *Gm15441*^+/+^ and *Gm15441*^LSL^ mice fed control diet or treated with WY-14643. DAPI staining was used as a counter stain to detect nuclei in liver sections. A pronounced increase in Gm15441 fluorescence was detected in the cytoplasm and nuclei of hepatocytes in livers from WY-14643-treated *Gm15441*^+/+^ mice. The *Gm15441* RNA signal was absent in livers from vehicle-treated *Gm15441*^+/+^ and *Gm15441*^LSL^ mice, and in livers from WY-14643-treated *Gm15441*^LSL^ mice (Fig. 4f). *Gm15441* transcript copy number was determined to be around 100 RNA molecules per hepatocyte (Supplementary Fig. 6a, b). These data validate *Gm15441*^LSL^ mice as an effective knockout mouse model and reveal that hepatic *Gm15441* expression is both nuclear and cytosolic evoking several possible mechanisms by which it regulates *Txnip*.

### Loss of *Gm15441* potentiates inflammasome activation by WY-14643-induced metabolic stimulation

**Fig. 5:**
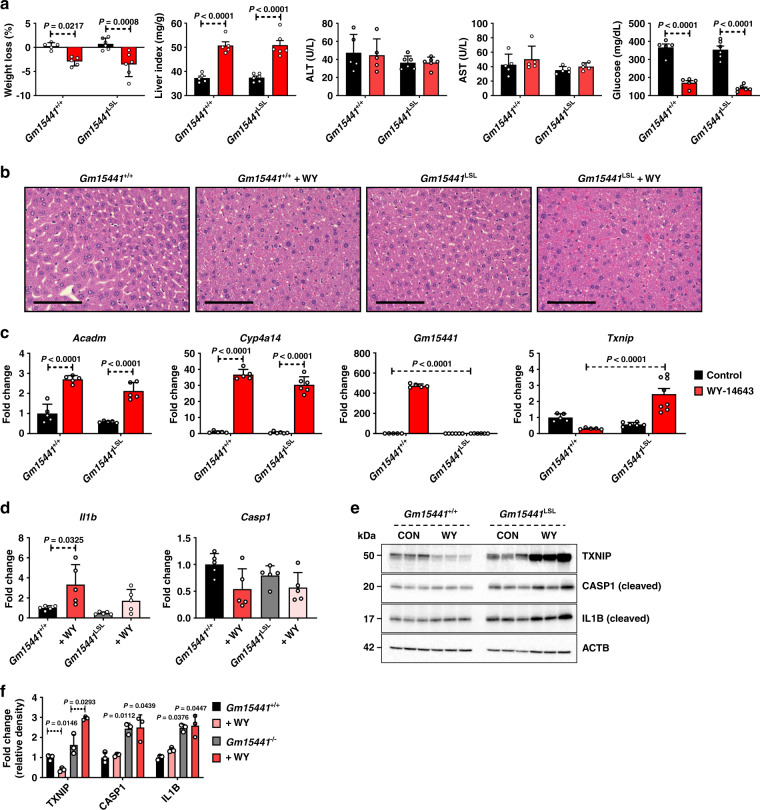
Loss of *Gm15441* potentiates inflammasome activation by WY-14643. **a** Physiological endpoints from *Gm15441*^+/+^ and *Gm15441*^LSL^ mice treated with WY-14643 for 48 h. Weight loss and liver indices (mg liver/g body mass; a measure of hepatomegaly), as well as serum ALT, AST, and glucose levels, in response to WY-14643 treatment. **b** Representative H&E staining from liver of *Gm15441*^+/+^ and *Gm15441*^LSL^ mice treated with WY-14643 for 48 h (*n* = 5, 3 images/mouse). Scale bars represent 100 nm (×400). **c** qRT-PCR analysis of *Acadm*, *Cyp4a14*, *Txnip*, and *Gm15441* expression in livers of mice treated with WY-14643 for 48 h. **d** qRT-PCR analysis of *Il1b* and *Casp1* mRNA in livers of mice treated with WY-14643. **e** Western blot analysis of TXNIP, ACTB, CASP1 (cleaved), and IL1B (cleaved) protein in livers from mice treated with WY-14643 for 48 h. **f** Densitometric analysis of TXNIP, CASP1 (cleaved), and IL1B (cleaved) protein levels (*n* = 3). Each data bar represents mean ± SD for *n* = 5 liver samples; red bars, WY-14643 treatment. Adjusted *p* values, provided in the panels, as determined by ANOVA with Tukey’s multiple comparison correction, two-sided for comparisons between WY-14643 and control livers of the same genotype, or as shown (dashed horizontal lines).

PPARα plays a role in attenuating inflammation in many tissues and disease models^41,42^. In the liver, PPARα ameliorates inflammation by reducing ER stress in hepatocytes^43^. TXNIP is known to facilitate ER stress-induced NLRP3 inflammasome activation^27,44^. By regulating TXNIP levels, Gm15441 could serve as a link between PPARα and the NLRP3 inflammasome. To ascertain whether lncRNA Gm15441 attenuates inflammasome activation by regulating TXNIP levels, mice were treated with WY-14643 for 48 h, and weight loss, liver index (mg liver/g total body mass), ALT, AST, and serum glucose levels were measured. There was no discernable difference in weight loss between *Gm15441*^+/+^ and *Gm15441*^LSL^ mice on a WY-14643 diet (Fig. 5a). Serum ALT and AST levels were unchanged by genotype or following WY-14643 treatment. Serum glucose levels were significantly decreased by WY-14643 in both *Gm15441*^+/+^ and *Gm15441*^LSL^ mice, but the decrease was significantly greater in *Gm15441*^LSL^ mice (Fig. 5a). However, insulin and glucose tolerance tests, performed on *Gm15441*^LSL^ mice, indicate that these animals are insulin sensitive (Supplementary Fig. 7a–d). Furthermore, pronounced hepatomegaly was induced by WY-14643 treatment in both *Gm15441*^+/+^ and *Gm15441*^LSL^ mice, as indicated by the significant increase in liver size (Fig. 5a, liver index), and by the pronounced swelling of hepatocytes seen by H&E staining (Fig. 5b). Expression of the PPARα target genes acyl-CoA dehydrogenase medium chain (*Acadm*) and *Cyp4a14* was significantly increased by WY-14643 in both *Gm15441*^+/+^ and *Gm15441*^LSL^ mice. *Gm15441* was strongly upregulated in livers of wild-type mice but was not detected in *Gm15441*^LSL^ mice. *Txnip* mRNA was significantly decreased by WY-14643 in livers of wild-type animals, consistent with Fig. 2, but was significantly induced in *Gm15441*^LSL^ livers (Fig. 5c). There was also a small but significant increase in *Il1b* mRNA expression in wild-type mice treated with WY-14643 (Fig. 5d). Basal levels of the inflammasome-related proteins TXNIP, cleaved CASP1 (p20), and cleaved IL1B were elevated in livers of *Gm15441*^LSL^ mice compared to wild-type mice, and further increased following WY-14643 treatment (Fig. 5e, f). Overall, levels of all three proteins were much higher in livers of WY-14643-treated *Gm15441*^LSL^ mice than wild-type mice (Fig. 5e). To test whether TXNIP is responsible for the phenotype observed in *Gm15441*^LSL^ mice, an adenovirus associated virus (AAV) expressing short hairpin (sh) *Txnip* (AAV-shRNA-*Txnip*) vector was generated. The AAV-shRNA-*Txnip* virus was injected into the tail vein of *Gm15441*^+/+^ and *Gm15441*^LSL^ mice, and following a recovery period, the mice were placed on a control diet or WY-14643-containing diet for 24 h. WY-14643-induced liver indices and histological changes were not affected by *Txnip* knockdown (Supplementary Fig. 8a, b, g). *Txnip* mRNA and protein expression were suppressed by *Txnip* knockdown, or by WY-14643 treatment, in *Gm15441*^+/+^ mice (Supplementary Fig. 8a, c, d); they were also suppressed by *Txnip* knockdown in WY-14643-treated *Gm15441*^LSL^ mice (Supplementary Fig. 8b, e, f). Cleaved CASP1 and IL1B protein were significantly suppressed by *Txnip* knockdown in WY-14643-treated *Gm15441*^+/+^ mice, but not in WY-14643-treated *Gm15441*^LSL^ mice (Supplementary Fig. 8c–f and Supplementary Table 4). These data support the view that *Gm15441* plays an important role in WY-14643 induced metabolic stress in the liver. Thus, the ablation of *Gm15441* increases the expression of TXNIP and maturation of two other proteins associated with NLRP3 inflammasome activation, namely, cleaved CASP1 and IL1B.

### Loss of Gm15441 potentiates inflammasome activation during physiological response to acute fasting

**Fig. 6:**
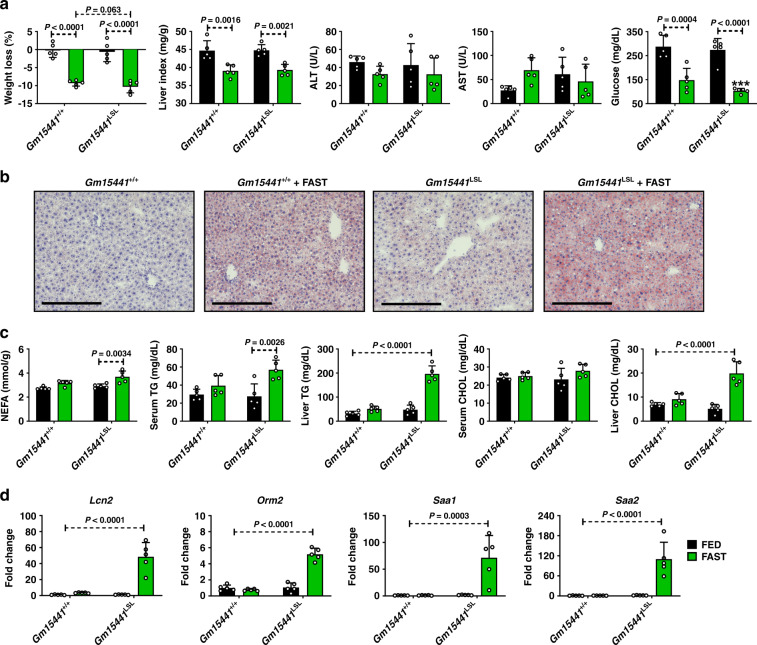
Loss of *Gm15441* potentiates inflammasome activation during physiological response to acute fasting. **a** Physiological endpoints from *Gm15441*^+/+^ and *Gm15441*^LSL^ mice after 24 h fasting. Weight loss and liver indices (mg liver/g body mass; a measure of hepatomegaly), as well as blood ALT, AST, and glucose levels, in response to fasting. **b** Representative ORO staining of liver tissues after a 24 h fed or fast in *Gm15441*^+/+^ and *Gm15441*^LSL^ mice (*n* = 5, 3 images/mouse). Scale bars represents 100 nm (×200). **c** Non-esterified fatty acid (NEFA) from serum, triglycerides (TG) and cholesterol (CHOL) levels from serum and liver tissues after a 24 h fast. **d** Analysis of *Apoa4*, *Bhmt*, *Lcn2*, *Orm2*, *Saa1*, and *Saa2* mRNAs in livers of *Gm15441*^+/+^ and *Gm15441*^LSL^ mice after 24 h fasting. Each data bar represents the mean ± SD for *n* = 5 liver samples; green bars, 24 h fasting. Adjusted *p* values, provided in the panels, as determined by ANOVA with Tukey’s multiple comparison correction, two-sided for comparisons between fast-stimulated and unstimulated livers of the same genotype, or as shown (dashed horizontal lines).

During fasting, hepatic PPARα facilitates the metabolic remodeling that promotes use of lipids as an alternate energy source^4^. Fasting is also associated with many anti-inflammatory effects^3^. To determine whether *Gm15441* regulates inflammasome activation during physiological fasting, *Gm15441*^+/+^ and *Gm15441*^LSL^ mice were fasted for 24 h. Serum ALT and AST were not changed between *Gm15441*^+/+^ and *Gm15441*^LSL^ mice, both with and without WY-14643 treatment (Fig. 6a). Fasting decreased serum glucose levels in both *Gm15441*^+/+^ and *Gm15441*^LSL^ mice, but the decrease was greater in *Gm15441*^LSL^ mice (Fig. 6a). Expression of *Acadm*, *Cyp4a14*, and *Txnip* mRNAs was significantly increased by fasting in both *Gm15441*^+/+^ and *Gm15441*^LSL^ mice (Supplementary Fig. 9a). *Gm15441* was significantly induced by fasting, although to a much lower degree than with WY-14643 treatment (Supplementary Fig. 9a). Expression of *Txnip* mRNA was increased to the same level in livers of fasted *Gm15441*^LSL^ mice as in fasted *Gm15441*^+/+^ mice (Supplementary Fig. 9a). Fasting also increased TXNIP, cleaved CASP1 (p20), and cleaved IL1B protein levels in *Gm15441*^LSL^ mice, in contrast to either a modest increase (TXNIP) or no increase (CASP1, IL1B) in *Gm15441*^+/+^ mice (Supplementary Fig. 9b, c). Fasting markedly promotes lipid accumulation in mouse liver^4^. Lipid droplet accumulation was also observed and more widespread in fasting *Gm15441*^LSL^ mice than fasting *Gm15441*^+/+^ mice (Supplementary Fig. 9d). Oil Red O staining was performed to validate lipid accumulation in response to fasting in *Gm15441*^LSL^ mice. A greater amount of lipid accumulation was observed in fasting *Gm15441*^LSL^ liver than in fasting *Gm15441*^+/+^ mouse liver (Fig. 6b). Expression of lipid metabolism-related genes was not significantly impacted in *Gm15441*^LSL^ mice under fasting conditions (Supplementary Fig. 10). Serum non-esterified fatty acids (NEFA), serum and liver triglyceride (TG) levels, and liver (but not serum) total cholesterol (CHOL) levels, were significantly elevated in fasting *Gm15441*^LSL^ mouse livers compared to fasting *Gm15441*^+/+^ mouse livers (Fig. 6c). These findings were supported by RNA-sequencing analysis, which revealed that liver damage and lipid accumulation-related genes were impacted by *Gm15441* deficiency, albeit in a complex manner (Supplementary Table 5). To check whether Gm15441 influences lipid mobilization or uptake, total lipase activity was measured in liver, skeletal muscle, and inguinal white adipose tissue (IWAT) from fed and fasted mice (Supplementary Fig. 11a–c). Total lipase activity was elevated in the livers of fasted *Gm15441*^LSL^ mice (Supplementary Fig. 11a). Total lipase activity in skeletal muscle was decreased by fasting in *Gm15441*^+/+^ mice and fed or fasted *Gm15441*^LSL^ mice when compared to fed wild-type mice (Supplementary Fig. 11b). However, the difference between fasted *Gm15441*^+/+^ and *Gm15441*^LSL^ mice was not significant (Supplementary Fig. 11b). Total lipid activity in IWAT was not significantly different between genotype with or without fasting (Supplementary Fig. 11c). Furthermore, analysis of the impact of fasting on mRNAs for lipocalin 2 (*Lcn2*), orosomucoid 2 (*Orm2*), serum amyloid A1 (*Saa1*), and serum amyloid A2 (*Saa2*) – biomarker genes for liver damage, inflammation, and/or fatty accumulation – showed these genes were significantly elevated with fasting in *Gm15441*^LSL^ compared to *Gm15441*^+/+^ mouse livers (Fig. 6d). ELISA results indicated serum SAA1 levels were also significantly increased by fasting in *Gm15441*^LSL^ mice (Supplementary Fig. 12). Fasting induced lower but still significant increases in several of these mRNAs in *Gm15441*^+/+^ livers, which could reflect the fasting-induced increase in *Txnip* expression (Supplementary Fig. 9a–d). Thus, *Gm15441* negatively regulates the NLRP3 inflammasome pathway and lipid accumulation, potentially by preventing *Txnip* expression, in response to fasting.

### Translational regulation of TXNIP by Gm15441 is partially dependent on 5′UTR sequences

**Fig. 7:**
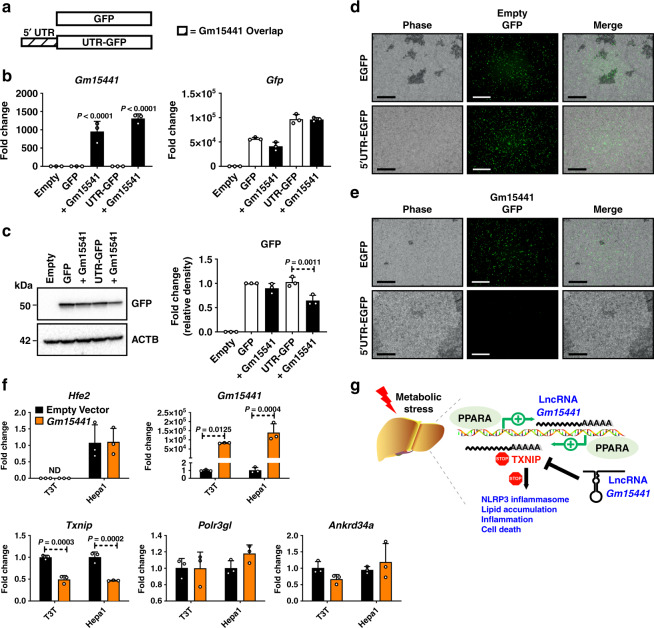
*Gm15441* regulates TXNIP translation in part through IRES sequences found within the 5′UTR of TXNIP. **a** Schematic representation of GFP construct inserts with or without the TXNIP 5′UTR sequence. **b** Analysis of *Gm15441* RNA and *Gfp* mRNA in Hepa-1 cells. **c** Western blot analysis of GFP protein and relative density of GFP signal from Hepa-1 cells (right). **d** Fluorescence of GFP in Hepa-1 cells transfected with GFP with empty or *Gm15441* plasmid DNA for 48 h (*n* = 3). Scale bars represent 20 nm (100x). **e** Fluorescence of GFP in Hepa-1 cells transfected with 5′ UTR sequence containing GFP with empty or *Gm15441* plasmid DNA for 48 h (*n* = 3). Scale bars represent 20 nm (100x). **f** Analysis of *Hfe2*, *Pol3gl*, and *Ankrd34a* mRNAs from Hepa-1 and NIHT3T cells transfected with empty plasmid or *Gm15441* expression vector for 24 h. Each data point represents mean ± SD for *n* = 3 replicates. Adjusted *p* values, provided in the panels, as determined by ANOVA with Tukey’s multiple comparison correction, one-sided for comparisons in the absence of Gm15441 (**b**, **c**) or to empty vector (**f**). **g** Model for role of Gm15441 in suppressing TXNIP-mediated inflammasome activation.

Translational regulation of TXNIP can occur via internal ribosome entry site (IRES) sequences found in the 5′UTR^45^. IRES sequences are commonly associated with cell survival- and stress response-related genes and utilized when cap-dependent translation is inhibited during nutrient deprivation^46^. The 5′UTR of *Txnip* contains binding sites for IRES trans-acting factors (ITAFs), which regulate IRES-mediated translation^45^. As *Gm15441* transcription generates an RNA antisense to the 5′UTR of *Txnip*, *Gm15441* RNA may block ITAF protein binding to the *Txnip* 5′UTR and thereby inhibit *Txnip* translation. To investigate this regulatory mechanism, the translational inhibitory potential of the *Txnip* 5′UTR was examined when linked to the coding sequence for GFP (Fig. 7a). The GFP and the 5′UTR-GFP expression vectors were each transfected into Hepa-1 cells together with a *Gm15441* expression vector or an empty expression plasmid. After 48 h, strong *Gm15441* expression was achieved, but there were no effects on the RNA levels of GFP or UTR-GFP (Fig. 7b). In contrast, GFP protein translated from the *Txnip* 5′UTR-GFP template was significantly reduced in cells co-transfected with *Gm15441* expression plasmid (Fig. 7c). This 5′UTR-dependent decrease in GFP protein was most striking when examined by fluorescent microscopy (Fig. 7d, e). These findings suggest that *Gm15441* regulates TXNIP translation through IRES sites found within its 5′UTR. In vitro transcription-translation assays performed using reticulocyte lysates provided additional evidence that *Gm15441* directly suppresses TXNIP protein levels, whereby increasing Gm15441 expression levels caused a corresponding decrease in TXNIP protein expression (Supplementary Fig. 13a, b). To examine whether lncRNA *Gm15441* impacts expression of genes that flank *Gm15441* on mouse chromosome 3, namely, *Hfe2 (Hjv)*, *Pol3gl*, and *Ankrd34a*, Hepa-1 and NIH3T3 mouse embryonic fibroblast cells were transfected with *Gm15441* expression vector, or empty plasmid. The *Gm15441* transgene was expressed and significantly downregulated *Txnip* without impacting the expression of *Hfe2*, *Pol3gl*, or *Ankrd34a* (Fig. 7f). Furthermore, TXNIP protein expression was suppressed by *Gm15441* expression in vitro (Supplementary Fig. 14a, b). Together, these data support a regulatory mechanism whereby PPARα induces *Gm15441*, which in turn attenuates inflammation through TXNIP and the NLRP3 inflammasome pathway (Fig. 7g).

## Discussion

The physiological alterations that accompany fasting impart several health benefits, including anti-inflammatory effects^47^. PPARα activation during fasting is a key regulatory event of lipid and glucose metabolism. A growing body of evidence indicates that PPARα activation also potently suppresses inflammation in several disease models and tissues^48^. However, the mechanism by which PPARα modulates metabolic stress-induced inflammation is not known. The direct regulation of protein-coding genes by PPARα is well characterized, but it was not known whether lncRNAs – which may influence gene expression through a variety of mechanisms^49^—also serve as direct targets contributing to physiological changes induced by PPARα activation. The present study identified several hundred PPARα-responsive liver-expressed lncRNA genes including several antisense lncRNAs, which often contribute to the regulation of genes on the opposing strand^50,51^. Further, a PPARα-dependent regulatory axis involving one such antisense lncRNA, *Gm15441*, was characterized. *Gm15441* was shown to be transcribed in a liver-specific, PPARα-dependent manner to yield an antisense lncRNA that protects against metabolic stress by suppressing expression of the opposing sense transcript, TXNIP, and thereby suppresses TXNIP-mediated NLRP3 inflammasome activation.

RNA-seq analysis of livers from wild-type and *Ppara*^−/−^ mice treated with the PPARα-specific agonist WY-14643 identified more than 400 liver-expressed lncRNA transcripts that were significantly upregulated or downregulated 48 h after WY-14643 treatment. Only six of these lncRNAs were similarly responsive to WY-14643 in *Ppara*^−/−^ mice, thus establishing the striking PPARα dependence of these non-coding RNA transcriptomic responses. PPARα-dependent, liver-specific expression was verified for six non-coding RNAs, and detailed functional studies were carried out on one such gene, *Gm15441*, which showed an unusually strong induction in WY-14643-treated liver. Importantly, the genomic orientation of *Gm15441* is antisense to that of *Txnip*. The *Txnip* gene encodes a ubiquitously-expressed protein that facilitates cellular responses to oxidative stress and inflammation^52^. TXNIP was originally identified as a negative regulator of thioredoxin^53^, and subsequent studies found that TXNIP contributes to a wide array of processes in several tissues. TXNIP is key regulator of NLRP3 inflammasome activation, which plays an important role in liver fibrosis and hepatocellular carcinoma^54^, and its activation is associated with the NLRP3 inflammasome pathway in human diseases^55^. TXNIP has also emerged as an important glucose sensor that regulates glucose uptake in response to insulin^56^ and plays an important role in metabolic stress^57,58^. An earlier study found that *Ppara* mRNA and PPARα target gene mRNAs were elevated in *Txnip*-null mice, and that *Txnip* expression attenuated PPRE-luciferase activity when co-transfected with PPARα/RXR and treated with WY-14643^59^, suggesting a *Txnip-Ppara* feedback regulatory loop that may also involve *Gm15441*.

LncRNAs regulate diverse cellular processes, including metabolism-related genes expressed on the opposing strand^60^. The present results showed that *Gm15441* negatively regulates *Txnip* expression during metabolic stress. LncRNA *Gm15441* is antisense to *Txnip*, whose mRNA is decreased in liver coincident with the robust increase in *Gm15441* expression beginning 3 h after activation of PPARα by WY-14643. The underlying PPARα regulatory interactions controlling activation of the *Gm15441* promoter were investigated, and several PPARα binding sites identified within 10 kb of the *Gm15441* TSS were confirmed by ChIP assays and shown to be functional in reporter gene assays. Thus, the TXNIP antisense lncRNA *Gm15441* is a direct target of PPARα in WY-14643-stimulated mouse liver.

Pharmacological activation of PPARα deregulates NLRP3 inflammasome activity in a mouse model of diabetes^61^ and prolonged hepatic NLRP3 inflammasome activation leads to hepatocyte death, inflammation, and fibrosis^62,63^. Mice treated solely with the PPARα agonist WY-14643 exhibit pronounced liver hepatomegaly, hepatocyte hypertrophy, and lipid accumulation. These changes are coupled to an increase in oxidative stress^64^. Oxidative stress is known to stimulate NLRP3 inflammasome activation and IL1B maturation^22–24,65,66^. Interestingly, differential gene expression and pathway analysis of RNA-sequencing data in wild-type mice treated solely with WY-14643 revealed activation of some acute inflammatory response pathways. Thus, the TXNIP-mediated NLRP3 inflammasome pathway was examined. *Txnip* mRNA was significantly decreased by PPARα activation in livers of wild-type *and Gm15441*^+/+^ mice, while TXNIP protein was elevated in livers of *Gm15441*^LSL^ mice. Furthermore, TXNIP-mediated NLRP3 inflammasome activation resulted in cleavage of CASP1 to its mature, proteolytically active p20 form. Mature CASP1 subsequently cleaves target proteins, including the pro-inflammatory cytokine IL1B to its corresponding mature, active form. Mature, cleaved CASP1 (p20) and IL1B protein were elevated in *Gm15441*^LSL^ mice, indicating an increase in inflammasome activation. Thus, PPARα activation of lncRNA Gm15441 suppresses the TXNIP-mediated NLRP3 inflammasome pathway in response to metabolic stress. PPARα plays an important regulatory role in the fasted mouse liver model^4^. Hepatic expression of PPARα target genes *Acadm* and *Cyp4a14* was significantly increased by fasting, as was the expression of *Txnip* mRNA, in livers of *Gm15441*^LSL^ mice as well as *Gm15441*^+/+^ mice compared with fed mice. However, TXNIP protein was more highly increased in fasting *Gm15441*^LSL^ mice compared to fasting *Gm15441*^+/+^ mice. Fasting *Txnip* mRNA levels are closely associated with lipid and glucose regulation^67^. In addition, TXNIP-mediated NLRP3 inflammasome pathway target protein cleavage (i.e., CASP1 and IL1B) was significantly elevated in livers of fasting *Gm15441*^LSL^ mice compared to *Gm15441*^+/+^ mice. These results support the proposal that physiological upregulation of lncRNA *Gm15441* in response to fasting prevents metabolic stress by suppressing the TXNIP-mediated NLRP3 inflammasome response.

PPARα, a key regulator of global lipid homeostasis, modulates fasting-induced lipid accumulation and hepatosteatosis in mice^4^. Lipid droplets appeared in livers of fasted *Gm15441*^+/+^ mice but were more widely distributed in livers of fasting *Gm15441*^LSL^ mice. Histological lipid staining revealed considerably larger amounts of lipid accumulation in livers of fasted *Gm15441*^LSL^ mice than in wild-type mouse livers. Moreover, serum and liver TG levels and liver CHOL levels were also significantly elevated by fasting in *Gm15441*^LSL^ mice. To determine whether *Gm15441* influences lipid mobilization or uptake, total lipase activity was measured in liver, skeletal muscle, and inguinal white adipose tissue (IWAT) from fed and fasted *Gm15441*^+/+^ and *Gm15441*^LSL^ mice. Total lipase activity in liver was elevated in fasted *Gm15441*^LSL^ mice after 24 h while fasting didn’t affect lipase levels in wild-type mice. Elevated hepatic lipase activity in fasted *Gm15441*^LSL^ mice is suggestive of an increase in lipid uptake. As lipids and TGs in the liver accumulate, spillover into circulation would be expected and may explain the observed increase. Lipase activity in the skeletal muscle of fed *Gm15441*^LSL^ mice was significantly lower than wild-type suggesting *Gm15441* may influence lipid uptake into this tissue. Interestingly, our data shows that basal *Gm15441* expression levels are considerably higher in skeletal muscle than liver suggesting that this lncRNA may have yet-to-be determined functions within other non-hepatic tissues. RNA-seq analysis revealed that liver damage, inflammation, and fatty accumulation biomarkers such as *Lcn2*, *Orm2*, *Saa1*, and *Saa2* mRNAs showed significantly higher expression under fasting-induced metabolic stress in *Gm15441*^LSL^ compared to wild-type. Hepatic *Lcn2* and *Orm2* mRNAs are both upregulated in the liver in response to IL1B^68,69^. SAA1 and SAA2 are major acute phase proteins which are activated in response to inflammation^70^. Activation is dependent on proteolytic cleavage by matrix metallopeptidases (MMPs), namely MMP9^71^. Hepatic MMP9 expression is responsive to IL1B activation^72^. Therefore, the increase in SAA1 and SAA2 further substantiates elevated NLRP3-induced inflammation in our model. Like SAA1 and SAA2, OSM2 is an acute phase protein associated with inflammation that is primarily produced in the liver^73^. Although the exact function of this protein has not been discovered, elevation of its expression is linked to several inflammatory diseases, suggesting that it is an important marker of inflammation^73–76^. Lipocalin-2 (LCN2) is also specifically associated with NLRP3 inflammasome activation^77^. The increase in mature, cleaved IL1B in *Gm15441*^LSL^ mice may at least partially explain the increased hepatic expression of *Lcn2, Orm2, Saa1*, and *Saa2* mRNAs in fasted *Gm15441*^LSL^ mice. Thus, these genes are all associated with inflammation/oxidative stress, consistent with the PPARα-dependent attenuation by *Gm15441* of TXNIP-associated hepatic inflammation in response to metabolic stress.

IRES is an RNA element that allows for translation initiation of certain mRNAs, including TXNIP, when cap-dependent translation is inhibited under stress conditions^45^. ITAFs such as polypyrimidine tract binding protein were shown to regulate TXNIP expression during nutrition starvation by binding to IRES sequences within the *Txnip* 5′UTR^45^. By binding to these IRES sequences, ITAF proteins regulate translation initiation and TXNIP protein expression. As lncRNA *Gm15441* is antisense to TXNIP and its exon 3 almost entirely overlaps the *Txnip* 5′UTR, *Gm15441* may block IRES sequences needed for translation of TXNIP. The ability of Gm15441 to modulate TXNIP protein levels via *Txnip* 5′UTR IRES sequences was demonstrated in vitro by co-transfection of a GFP reporter with a *Gm15441* expression vector. A decrease in GFP expression was dependent on both *Gm15441* and the *Txnip* 5′UTR, indicating that *Gm15441* in part regulates TXNIP through its 5′UTR, presumably by masking functional IRES sites. In vitro transcription-translation assays provided additional evidence that *Gm15441* directly suppresses TXNIP protein levels. Incrementally increasing Gm15441 expression caused a corresponding decrease in TXNIP protein suggesting that translation was repressed. Together, these data support a mechanism whereby Gm15441 translationally regulates TXNIP protein levels through IRES sites found within the TXNIP 5′UTR.

The strand-specific *Gm15441* knockout mouse developed in this study enabled us to assess the function of this lncRNA without impacting regulation of its antisense protein-coding partner, *Txnip*. In contrast, previous knockout mouse models developed to evaluate TXNIP function disrupt both *Txnip* and *Gm15441*, and therefore abolish the unique regulatory loop that exists between these two genes. *Txnip*-null mice show a higher incidence of hepatocellular carcinoma, with approximately 40% of male mice developing hepatic tumors^78^. However, the *Txnip*-null mice lack exons 1–4, and thus *Gm15441* is also ablated^79^. TXNIP expression in vitro inhibited hepatocellular carcinoma cell proliferation and induced apoptosis^80^. Thus, prolonged *Gm15441*-mediated suppression of TXNIP may also contribute to PPARα agonist induced hepatocellular carcinoma^81^.

In summary, the present study focused on the role of lncRNAs as potential metabolic regulators. Pharmacological activation of PPARα promoted induction of large numbers of hepatic lncRNAs, a subset of which are also targeted by the nuclear receptors CAR and PXR. *Gm15441* was identified as a liver-specific, PPARα-dependent lncRNA positioned antisense to the pro-inflammatory gene *Txnip*. Gm15441 expression was shown to suppress TXNIP protein by a mechanism involving the blocking of IRES sites within its 5′UTR. By suppressing TXNIP translation, Gm15441 inhibited TXNIP-mediated activation of the NLRP3 inflammasome as well as subsequent CASP1 cleavage and IL1B maturation. Taken together, these results demonstrate that lncRNA *Gm15441* can directly prevent metabolic stress-induced inflammation and represents a therapeutic target for the treatment of inflammatory disorders.

## Methods

### Mouse models

Male 8- to 12-week-old mice were used for all studies and all mouse strains were on the C57BL/6J background and maintained on a grain-based control diet (NIH-31). Mice were housed in light (12 h light: 12 h darkness cycle) and temperature-controlled rooms (humidity 40–60%) and were provided with water and pelleted chow *ad libitum*. For pharmacological studies, the mice were provided a grain-based control diet or matched diet containing 0.1% WY-14643 for 24 or 48 h. For monitoring the time dependence of gene responses, WY-14643 was dissolved in 1% carboxymethyl cellulose (CMC) solution and orally administered by gavage (50 mg/kg in 200 μl). At the end of the treatment period, the mice were killed by CO_2_ asphyxiation and tissues harvested. For physiological studies, food was removed for 24 h starting shortly after the onset of the light cycle and endpoints collected at the same time the following day. Animals were then killed, and tissue samples harvested for further analysis. Blood was collected by venipuncture of the caudal vena cava. All animal experiments were performed in accordance with the Association for Assessment and Accreditation of Laboratory Animal Care international guidelines and approved by the National Cancer Institute Animal Care and Use Committee.

### Generation of *Gm15441*-null mice

SAGE Laboratories (Cambridge, UK) provided design and construction services for the CRISPR/Cas gene targeting technologies used to create a *Gm15441*-null (*Gm15441*^LSL^) mouse line. The targeting strategy results in the insertion of a floxed cassette containing a transcriptional stop repeat within the first intron of *Gm15441* (NR_040409.1) (Supplementary Table 6). Presence of the cassette prevents *Gm15441* expression. Crossing with a *Cre* mouse line removes the stop cassette and allows *Gm15441* expression to proceed. Microinjection-ready sgRNA, *Cas9* mRNA, and a plasmid donor with a floxed stop cassette were purchased from SAGE Laboratories. The sgRNA, Cas9 mRNA, and plasmid donor were then injected into C57BL/6J mouse embryos by the Transgenic Mouse Model Laboratory at the National Cancer Institute (Fredrick, MD) using the manufacturer’s recommended protocol. Founder animals were genotyped using primer sets in Supplementary Table 7, and all modifications were confirmed by targeted sequencing. Homozygous mice were then backcrossed ten times into the C57BL6 background to breed out any off-target effects.

### Mouse genotyping

Genomic DNA was extracted from mouse tail snips using the Sigma REDExtract-N-Amp Kit. Genotyping was performed following the manufacturer’s protocol with the following modifications. Twenty five µl of E buffer and 7 ul of TPS buffer were added to each snip and the samples stored room temperature for 10 min. Samples were heated at 95 °C for 5 min then 25 µl of N buffer was added. Polymerase chain reactions for genotyping *Gm15441* mice used the following primers: forward: 5′-TGCGAGGCACGATATGGCGA-3′, reverse: 5′-AGCGCACCTGTCACTTTCCTGC-3′. Bands of 1200 bp or 1700 bp were detected in wild-type and *Gm15441*^LSL^ mice, respectively.

### Preparation of adeno-associated virus (AAV) expressing short hairpin (sh) RNA targeting TXNIP

For the TXNIP knockdown studies, AAV gene delivery vectors were constructed by cloning TXNIP shRNA sequences into an AAV-shRNA plasmid (Addgene #85741, Watertown, MA). The AAV-shRNA expression plasmid was digested with XbaI and BamHI and a shRNA sequence (5′-GGACTACTTGCGCTATGAAGA-3′) targeting the first exon of TXNIP was inserted. AAV2/8 virus was generated by transfecting HEK293T cells with the pAAV2 insert containing either shRNA control or shRNA-Txnip under the control of the mouse U6 promoter, and flanked by serotype-2 inverted terminal repeats, pXR1 containing rep and cap genes of AAV serotype-8, and pHelper encoding the adenovirus helper functions (Addgene, Watertown, MA). Cell lysates were subjected to three rounds of freeze/thaw and then treated with 1 ul of benzonase (Sigma St. Louis, MO) per 5 ml of lysate for 30 min at 37 °C and clarified by centrifugation. Virus was purified by iodixanol gradient ultracentrifugation and titered by qRT-PCR. Mice 11-12 weeks old were given AAV-shRNA-*Txnip* or AAV-shRNA-Ctrl at a dose of 1 × 10^12^ genome copies/mouse by tail vein injection. Six weeks later, the mice were given a second AAV injection and allowed another two weeks to recover. Mice were then provided with control or WY-14643-containing diets and killed 24 h later.

### RNA isolation, library construction, and RNA sequencing

RNeasy Plus Mini Kits (Qiagen, Valencia, CA, USA) were used to extract total RNA from livers from four different treatment and control groups, namely wild-type and *Ppara*^−/−^ mice treated with control or WY-14643-containing diets. Mice were fed either control diet or fed a matching diet containing 0.1% WY-14643 for 48 h. All mice were killed between 1 and 3 PM. RNAs were extracted from *n* = 9 to 15 independent livers per group and RNA quality assessed using a TapeStation 4200 (Agilent, Santa Clara, CA, USA). High quality RNA samples (RIN > 9.0) were pooled, as described below, and used to construct stranded RNA-seq libraries from polyA-selected total liver RNA using an Illumina stranded TruSeq mRNA Prep Kit (Illumina, San Diego, CA, USA). Three pooled RNA samples were prepared for each of the four treatment groups with each pool comprised of *n* = 3-5 individual liver samples. The libraries were subjected to paired-end sequencing using an Illumina HiSeq 2500 instrument (Illumina) at the NCI-CCR sequencing facility (Frederic, USA) at a depth of 30-42 million read pairs for each of the 12 RNA-seq libraries. For RNA-seq analysis of *Gm15441*^LSL^ mice, total liver RNA was isolated from wild-type mice and *Gm15441*^LSL^ mice fed either control diet or a diet containing 0.1% WY-14643 for 24 h and killed between 1 and 3 PM. RNAs were extracted from *n* = 9 independent livers per experimental group, analyzed for RNA quality (RIN > 9.0), and used to prepare sequencing libraries. Three pooled RNA samples were prepared for each of the 4 treatment groups, with each pool comprised of *n* = 3 individual liver RNA samples. Libraries were subjected to 150 cycle paired-end Illumina sequencing at a depth of 13–21 million read pairs for each of the 12 RNA-seq libraries.

### Analysis of sequencing results

Data were analyzed using a custom RNA-seq analysis pipeline^18,82^. Briefly, sequence reads were mapped to the mouse genome (release mm9) using TopHat2 (v2.1.1)^83^. Genomic regions that contain exonic sequence in at least one isoform of a gene (exon collapsed regions^82^;) were defined for each RefSeq gene and for each lncRNA gene. HTSeq (0.6.1p1) was then used to obtain read counts for exon collapsed regions of RefSeq genes, and featureCounts (1.4.6-p5) was used to obtain read counts for exon collapsed regions of lncRNA genes. A set of 24,197 annotated mouse RefSeq genes (which includes some RefSeq lncRNAs) and a set of 15,558 liver-expressed lncRNA genes^17,18^ was considered for differential expression analysis. These lncRNAs include intergenic lncRNAs, as well as lncRNAs that are antisense or intragenic with respect to RefSeq genes, and were discovered using a computational pipeline for lncRNA discovery described elsewhere^16^ based on 186 mouse liver RNA-seq datasets representing 30 different biological conditions. RefSeq and lncRNA genes that showed significant differential expression following exposure to WY-14643 were identified by EdgeR as outlined elsewhere^16^. Genes dysregulated with an expression fold-change (i.e., either upregulation or downregulation) >2 at a false discovery rate (FDR), i.e., an adjusted *P*-value < 0.05 were considered significant and are shown in Supplementary Table 1 and Supplementary Table 5. Raw and processed RNA-seq data are available at GEO (https://www.ncbi.nlm.nih.gov/gds) accession numbers GSE132385 and GSE132386.

### Cell culture and transfection

Primary hepatocytes were isolated from C57BL6N mice as previously reported^84^ and seeded on collagen-coated 12-well plates (Becton Dickinson and Company, Franklin Lakes, NJ) at a density of 2 × 10^5^ cells in Williams’ Medium E (Thermo-Fisher Scientific, Waltham, MA) supplemented with 5% FBS and penicillin/streptomycin/amphotericin B solution (Gemini Bio-products, West Sacramento, CA). Hepa-1 mouse hepatoma cells and NIHT3T mouse embryonic fibroblast cells (ATCC, Manassas, VA) were maintained at 37 °C in a humidified atmosphere of 5% CO_2_ in Dulbecco’s Modified Eagle Medium (DMEM) containing 10% Fetal Bovine Serum (FBS) and 1% of penicillin/streptomycin mixture (Invitrogen, Waltham, MA). pCMV-EGFP plasmid was purchased from Addgene (Addgene #11153, Watertown, MA). pCMV-Txnip plasmid was purchased from Origene (Origene #PS10001). Spliced Gm15441 transcript insert was cloned into pCMV-EGFP to generate pCMV-Gm15441. One day after seeding, Hepa-1 and NIHT3T cells were transfected with recombinant pCMV-Gm15441 and pCMV-Txnip for 48 h. The cells were then harvested and analyzed by qRT-PCR and western blotting. For 5′UTR transfection experiments, the 5′UTR sequence from *Txnip* was inserted into pCMV-EGFP plasmid followed by transfection in Hepa-1 cells for 48 h. Non-5′UTR inserted pCMV-EGFP was used as a negative control. The cells were subjected to microscopic analysis or harvested and subjected to qRT-PCR and western blotting.

### Histological staining

Fresh liver tissue was immediately fixed in 10% phosphate-buffered formalin for 24 h and then processed in paraffin blocks. Four-micrometer sections were used for H&E staining. For Oil Red O (ORO) staining, fresh liver tissue was placed into a cryomold and filled with OCT Compound (Tissue-Tek), then transferred to a beaker of isopentane prechilled in liquid nitrogen. Sections were processed by HistoServ, Inc. (Germantown, MD). Slide imaging was performed using a Keyence BZ-X700 series all-in-one microscope with both ×20 and ×40 objectives, ×200 and ×400 magnification, respectively.

### Serum biochemistry

Blood was collected from mice and transferred to BD Microtainer Serum Separator Tubes (Becton Dickinson, Franklin Lakes, NJ). Serum was flash frozen in liquid nitrogen and stored at -80C. Serum chemistry analysis for total cholesterol (CHOL), non-esterified fatty acids (NEFA), and triglycerides (TG) was performed using Wako Clinical Diagnostics kits (WakoUSA, Richmond, VA). Serum alanine aminotransferase (ALT) and aspartate aminotransferase (AST) levels were measured using Catachem VETSPEC Kits as recommended by the manufacturer (Catachem, Oxford, CT). Blood glucose (GLU) levels were measured using a Contour blood glucose meter (Bayer, Mishawaka, IN). Serum SAA1 analysis was performed using Mouse Serum Amyloid A Quantikine ELISA Kit (R&D Systems, Minneapolis, MN).

### Metabolic assays

For the glucose tolerance tests (GTT), mice were fasted overnight for 16 h. For the insulin tolerance tests (ITT), the mice were fasted for 4 h. Glucose at 2 g/kg or insulin (Eli Lilly, Washington, DC) at 0.8 U/kg in saline were injected intraperitoneally into animals and blood glucose measured from tail bleeds using a Contour Glucometer (Bayer, Mishawaka, IN). Blood glucose was measured before the injection and at intervals of 15 min for 2 h post injection using the glucometer.

### Western blot analysis

Whole-cell extracts were prepared from mouse liver tissue or mouse primary hepatocytes using RIPA buffer supplemented with Halt Protease and Phosphatase Inhibitor Cocktail (Thermo-Fisher Scientific) and 1 mM PMSF. Protein concentrations were determined using the Pierce BCA Protein Assay Kit (Pierce, Rockford, IL). Twenty to forty μg of protein lysate was loaded per lane on a 4–12% Criterion TGX Precast Gel (Bio-Rad) then transferred to PVDF membranes using a Trans-Blot Turbo Transfer System (Bio-Rad). Membranes were blocked in 5% nonfat milk followed by an overnight incubation with primary antibodies targeting TXNIP (Novus #NBP1-54578), CASP1 (Invitrogen #14-9832-82), or IL1B (Cell Signaling #12242) at 4 °C. Primary antibodies were diluted at 1:500 before use. Following primary antibody incubation, the blots were washed and incubated with HRP-conjugated secondary antibodies for one hour (Cell Signaling #7074S, #7076S). Secondary antibodies were diluted at 1:5000 before use. The blots were then stripped using Restore Western Blot Stripping Buffer (Thermo-Fisher Scientific) and re-probed with alternate antibodies. An antibody against ACTB (Cell Signaling #8457L) was used as a loading control. Blot imaging was performed on a ChemiDoc MP System (Bio-Rad) after exposing the blot to Clarity Western ECL Blotting Substrate (Bio-Rad). Protein expression was quantitatively analyzed using band density and ImageJ software (NIH, Bethesda MD)^85^.

### Quantitative reverse transcription PCR assays

Total RNA was isolated from fresh mouse liver, mouse primary hepatocytes, and Hepa-1 cells using TRIzol Reagent (Thermo-Fisher Scientific, Waltham, MA, USA) and quantified using a NanoDrop Spectrophotometer (NanoDrop Products, Wilmington, DE, USA). Total RNA (2 μg) was reverse transcribed using All-in-One cDNA Synthesis SuperMix (BioTool, Houston, TX, USA). qRT-PCR analysis was performed using SYBR Green qPCR Master Mix (BioTool). Primers were designed for gene specificity and to cross exon-exon junctions using Primer-BLAST (www.ncbi.nlm.nih.gov/tools/primer-blast/) and purchased from IDT DNA Technologies (Coralville, IA, USA) (Supplementary Table 7). qRT-PCR experiments were designed and performed according to Minimum Information for Publication of Quantitative Real-Time PCR Experiments (MIQE) guidelines^86^. Results are normalized to actin expression. Values given are fold over control or relative expression value, where appropriate, calculated using the 2ΔCt QPCR calculation method^87^.

### Luciferase reporter assays

For luciferase assays, pSG5-PPARα (mouse) and pSG5-RXRA (mouse) were used for transcription factor expression^88^. Custom GeneBlocks (IDT DNA) were synthesized containing the predicted PPRE sites for Gm15441. GeneBlocks were digested and purified using a Qiagen PCR Purification Kit (Qiagen, Valencia, CA), and cloned into the pGL4.11 or pGL4.27 for PPRE reporter constructs (Promega, Madison, WI) using a BioRad Quick Ligation Kit (BioRad, Hercules, CA, USA). Prior to performing assays, all constructs were confirmed by Sanger sequencing at the NCI Center for Cancer Research Genomics Core. The phRL-TK renilla luciferase construct was used as a control to normalize for transfection efficiency. Primary hepatocytes were seeded into 12-well plates (4 × 10^4^ cells/well). PPRE reporter constructs were co-transfected into hepatocytes with PPARα and RXR expression vectors. PPRE-luc plasmid containing an *Acox1* PPRE site repeat was used as a positive control^89^. Empty pGL4.11 or pGL4.27 plasmids were used as negative controls. Plasmids were transfected using Lipofectamine 3000 Reagent (Thermo-Fisher Scientific). Luciferase activities were measured and plotted relative to lysate protein concentrations using the Promega Dual Luciferase Reporter (Promega) assays according to the manufacturer’s protocol. Measurements were taken on a Veritas microplate luminometer (Turner Biosystems, Sunnyvale, CA, USA).

### Chromatin immunoprecipitation

Chromatin was prepared from hepatocytes for ChIP assays as previously described^90^. Cells were fixed with 4% paraformaldehyde for 15 min, then glycine was added to a final concentration of 0.125 M and incubated for 10 min before harvesting. Chromatin was sonicated using a Bioruptor Pico (Diagenode, Denville, NJ, USA). Chromatin preparations were subjected to ChIP using a ChIP-IT High Sensitivity Kit and Protein G Agarose Prepacked Columns (Active Motif, Carlsbad, CA, USA) using either PPARα (Abcam Ab24509) antibody, normal rabbit IgG (Cell Signaling Technologies #2729S), or Histone H3 (Cell Signaling Technologies #4620) antibody. Rabbit IgG and Histone H3 were used as negative and positive controls, respectively. DNA was purified and concentrated using MinElute Reaction Cleanup columns (Qiagen). qRT-PCR and conventional PCR were performed using 2 μl of ChIP DNA samples from the 50 μl of purified samples using gene-specific primers (Supplementary Table 7). Cycle threshold (Ct) values of ChIP and input samples were calculated and presented as fold change.

### RNA fluorescent in situ hybridization (FISH)

For FISH staining of Gm15441, *Gm15441*^+/+^, and *Gm15441*^LSL^ mice were fed either a control diet or diet containing WY-14643 for 36 h. Livers were harvested, fixed in 4% of paraformaldehyde overnight then sent to the Molecular Pathology Laboratory at the National Cancer Institute for processing. RNA FISH experiments were performed using custom RNAscope probes and reagents developed by Advanced Cell Diagnostics (Newark, CA). Proprietary FISH probes targeted a region of *Gm15441* that does not overlap with *Txnip* to prevent signal interference (Supplementary Table 8). Slide imaging was performed using Aperio ImageScope software (Leica Biosystems, Buffalo Grove, IL, USA).

### Tissue total lipase activity assays

Liver, skeletal muscle (soleus), and inguinal white adipose tissue (IWAT) were homogenized in ice-cold PBS buffer using a Percellys bead homogenizer and 1 mm zirconia/silica beads. Tissue total lipase activity was assayed using a fluorometric lipase activity assay kit (Abcam #ab204721) following the manufacturer’s recommendations and normalized to total protein. According to the manufacturer, assay specificity has been confirmed for LPL, hormone-sensitive lipase [also known as (a.k.a.) LIPE], hepatic TG lipase (a.k.a. LIPC), and pancreatic TG lipase (a.k.a. PNLIP). Adipose TG lipase (a.k.a. PNPLA2) activity should also be detectable, although specificity has not directly been confirmed. Protein concentrations were determined using a Pierce BCA Protein Assay Kit (Thermo-Fisher).

### In vitro transcription-translation assay

TNT T7 Quick Coupled Transcription/Translation System was used for checking transcription and translation (Promega) according to the manufacturer’s protocol. Linear pCMV-Empty, pCMV-Gm15441 (GenScript custom gene synthesis construct containing mature, spliced Gm15441 transcript; NR_040409.1), and pCMV-Txnip (Origene; MR206196; NM_023719) plasmids were used as templates. All plasmids included the T7 promoter sequence for RNA transcription. pCMV-Txnip plasmid (0.5 µg) was added to reticulocyte lysate in combination with increasing amounts (0.1, 0.3, and 0.5 µg) of pCMV-Empty or pCMV-Gm15441 plasmid. Western blots were then performed on lysates using anti-TXNIP antibody as described above. Blot imaging was performed on a ChemiDoc MP System (Bio-Rad) after exposing the blot to Clarity Western ECL Blotting Substrate (Bio-Rad).

### 5′ Rapid amplification of cDNA ends (5′ RACE)

ExtractSTART Eukaryotic mRNA 5′-& 3′-RACE kit was used for cDNA library preparation according to the manufacturer’s protocol (Epicentre Biotechnologies, Illumina). 2 µg of total RNA from livers of mice treated with WY-14643 for 24 h was used for template. All steps followed the manufacturer’s protocol. PCR products were analyzed by Sanger Sequencing (2-ABI 3500xL and 1-3730xL DNA sequencers, Illumina) after cleanup using a PCR purification kit (Qiagen). The sequencing primers are listed in Supplementary Table 6.

### Data and statistical analyses

PPARα ChIP-seq data was downloaded from NCBI Gene Expression Omnibus (GSE61817). ChIP-seq data was uploaded to the Galaxy public server at usegalaxy.org for analysis^91^. More specifically, data were converted to bigwig file format using Galaxy tools. Bigwig files were then visualized using CLC workbench (Qiagen) and Integrated Genome Browser (version 9.0.0)^92^. All results are expressed as means ± SD. Significance was determined by t-test or one-way ANOVA with Bonferroni correction using Prism 7.0 software (GraphPad Software, La Jolla, CA, USA). A *P* value less than 0.05 was considered significant and statistical significance is indicated in the figure legends.

### Reporting summary

Further information on research design is available in the Nature Research Reporting Summary linked to this article.

## Supplementary information

Supplementary Information

Description of Additional Supplementary Files

Supplemental Data 1

Supplemental Data 2

Supplemental Data 3

Reporting Summary

## Data Availability

The data that support the findings of this study are available from the corresponding authors upon request. Supplementary information are provided in Supplementary Figs. 1–14, Supplementary Tables 1–8, and Supplementary Data 1–3. RNA-seq data have been deposited in the Gene Expression Omnibus under the accession code GSE132385 and GSE132386. PPARA ChIP-seq data in Fig. 3a was downloaded from Gene Expression Omnibus under the accession code GSE61817. Source data are provided with this paper.

## Source data

Source Data
