# Supplementary material for: Long non-coding RNA Gm15441 attenuates hepatic inflammasome activation in response to PPARA agonism and fasting
Source: Nat Commun. 2020 Nov 17;11:5847. doi: 10.1038/s41467-020-19554-7 (PMC7673042; doi:10.1038/s41467-020-19554-7)
Supplement: Supplementary file 1 — Supplementary Information [file 41467_2020_19554_MOESM1_ESM.pdf]

Supplemental information

Long non-coding RNA Gm15441 attenuates hepatic inflammasome activation in response to PPARA agonism and fasting

Brocker CN, Kim D et al.,

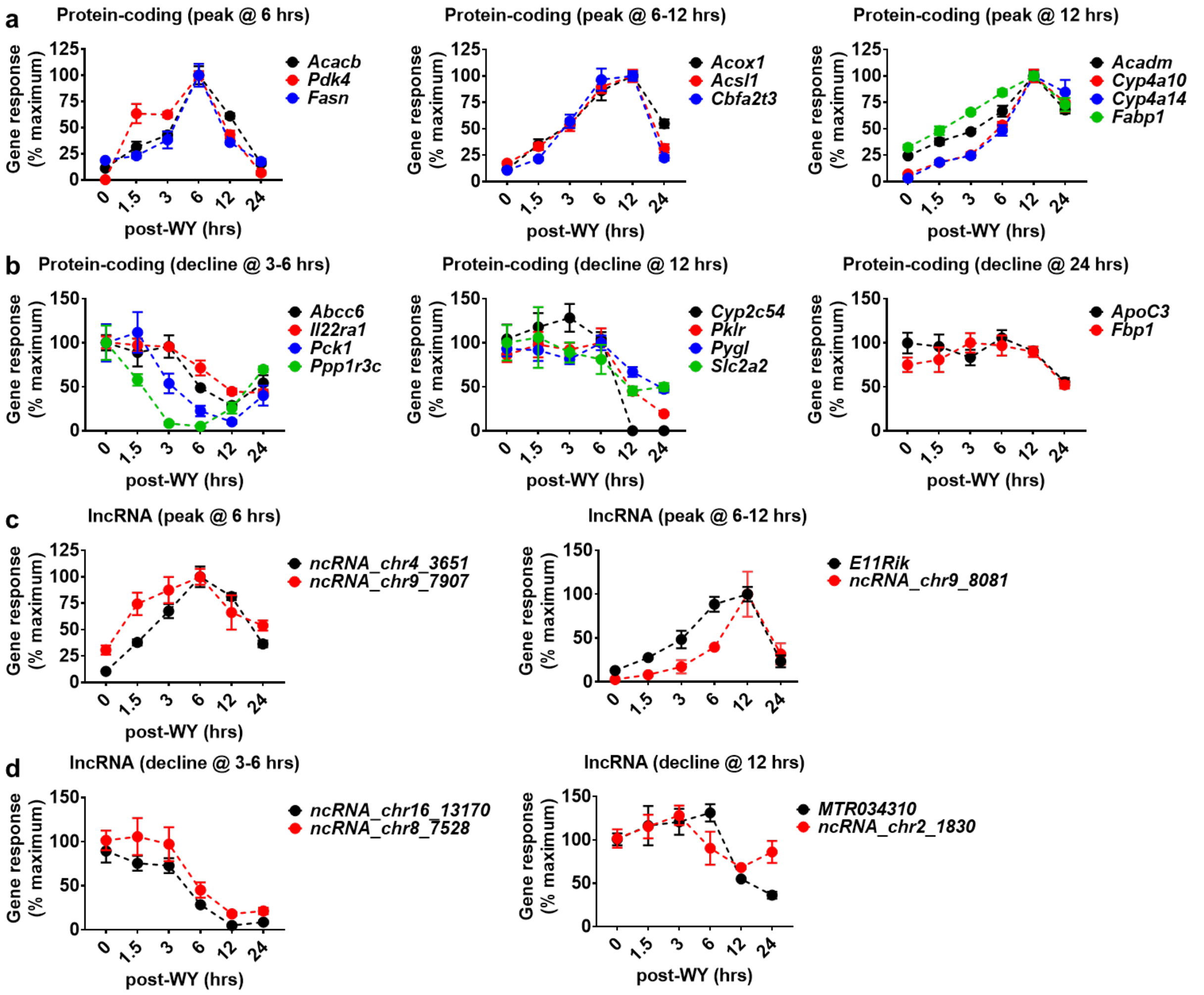

Supplementary Figure 1. PPARα-dependent lncRNA expression profiles are analogous to protein-coding genes.

Ppara$^{+/+}$ mice gavaged with the PPARα agonist WY-14643 (50 mg/kg). Livers were collected at t = 0, 1.5, 3, 6, 12, and 24 hours post-treatment. **a**, Analysis of upregulated protein-coding PPARα target gene response by qRT-PCR (n = 4). **b**, Analysis of downregulated protein-coding gene response by qRT-PCR (n = 4). **c**, Analysis of upregulated PPARα target lncRNAs response by qRT-PCR (n = 4). **d**, Analysis of downregulated PPARα target lncRNAs response by qRT-PCR (n = 4). Each bar represents the mean ± SD.

a

NO.	Transcript ID	Index	CNIT Score
1	NR_040409.1 Mus musculus predicted gene 15441 (Gm15441), long non-coding RNA	noncoding	-0.33681608841

b

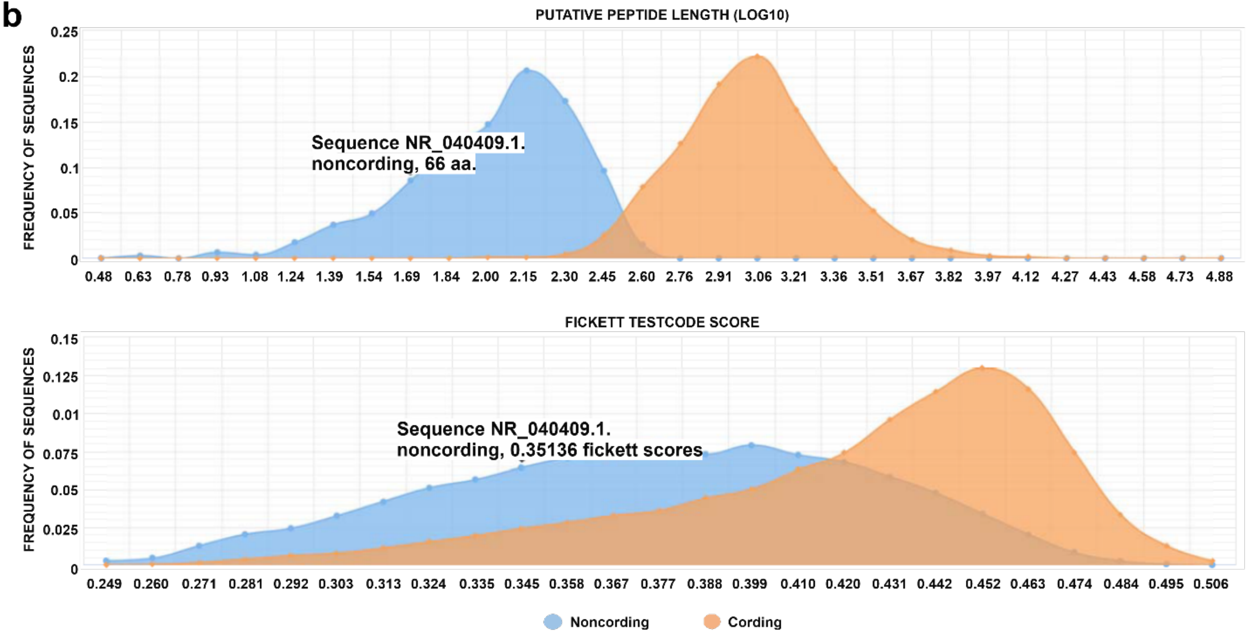

Supplementary Figure 2. Gm15441 transcript coding potential characterization.

a, Gm15441 protein-coding potential prediction by CNIT. **b**, Gm15441 protein-coding potential prediction by CPC2.

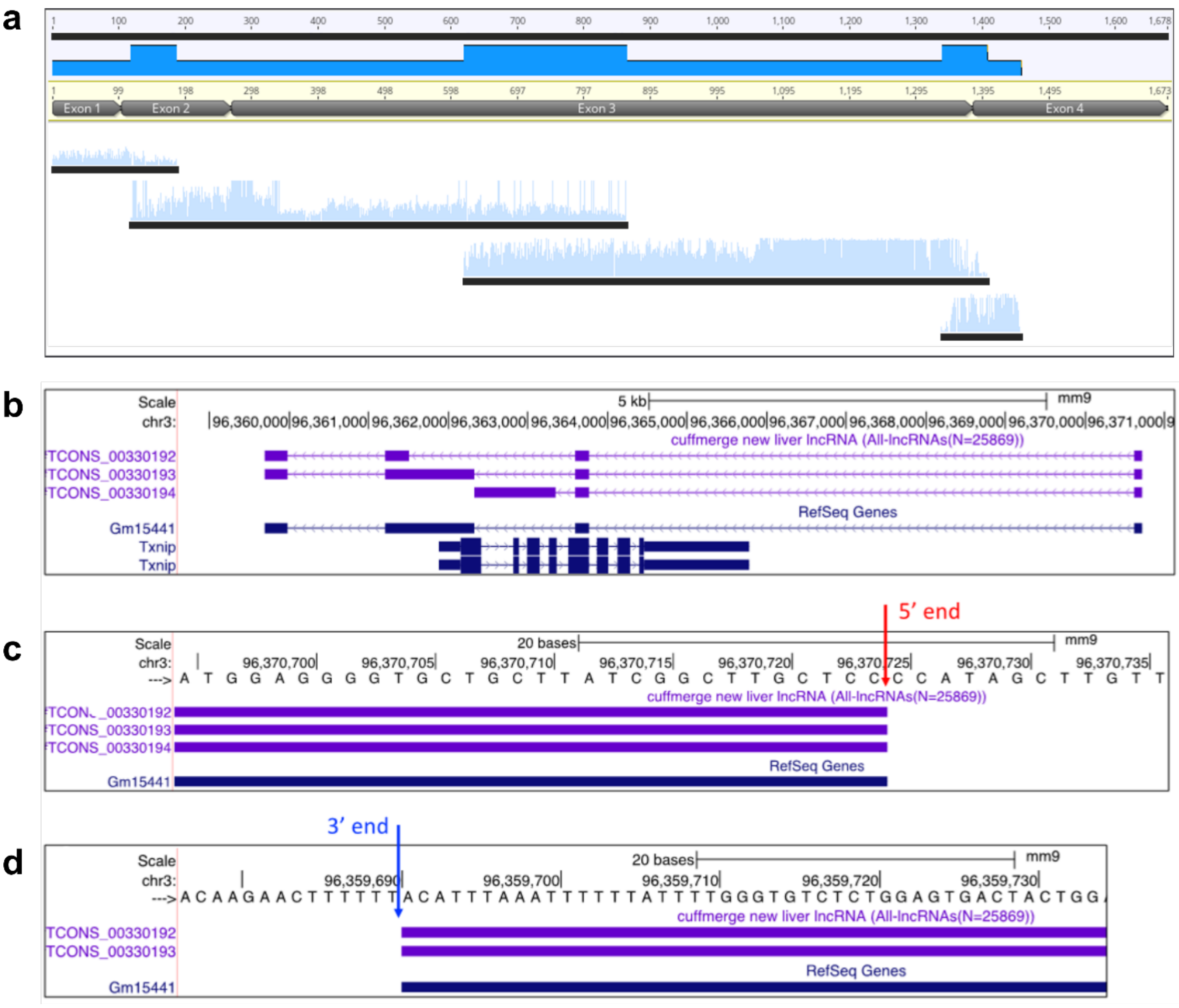

Supplementary Figure 3. Gene and structure analysis of Gm15441.

a. Alignment of assembled sequences from 5’ RACE end products aligned to *Gm15441* transcript annotation (NR_040409.1). **b**. Three isoforms for *Gm15441* identified by CuffMerge. **c**. All three isoforms share the precise 5' end. **d**. Two full-length isoforms share a common 3' end. mm9 mouse coordinates are as indicated.

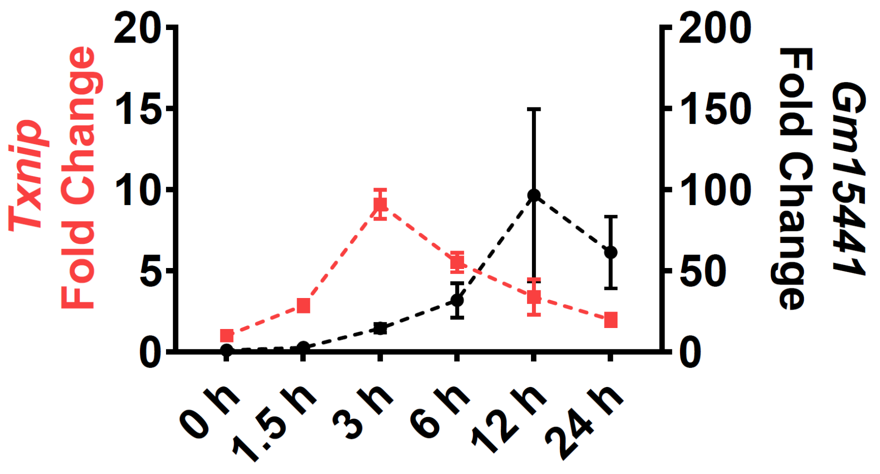

Supplementary Figure 4. Time course for changes in expression of *Gm15441* and *Txnip* mRNA over a 24 hour fasting period.

Relative changes in hepatic *Gm15441* and *Txnip* mRNA expression by qRT-PCR over a 24 hour fast. Each data point represents the mean ± SD for n = 5 liver samples.

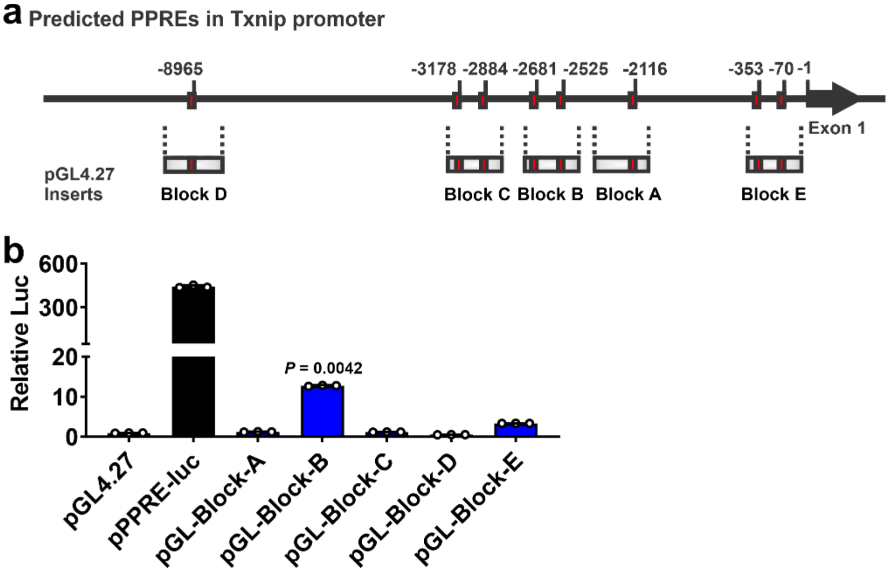

Supplementary Figure 5. TXNIP is a direct PPARα target gene.

a, Schematic representation of five predicted PPRE sequences found within the mouse *Txnip* promoter (-10 kb). **b**, Luciferase-based reporter assays identified functionally active PPREs within the *Txnip* promoter. Each bar represents the mean ± SD for n = 3 replicates. Adjusted *p* values, provided in the panels, as determined by ANOVA with Tukey's multiple comparison correction, one-sided for comparisons to pGL4.27 empty vector.

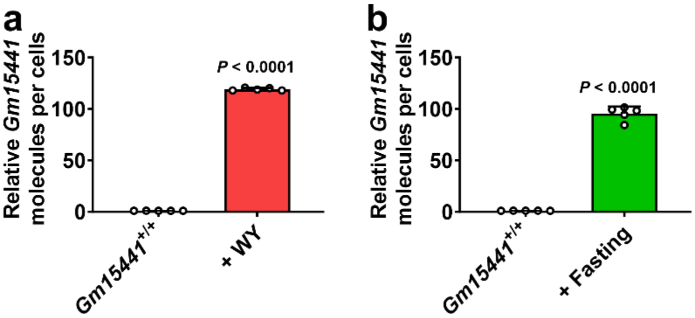

Supplementary Figure 6. *Gm15441* transcript copy number per hepatocyte.

a, Transcript copy number in livers of mice treated with WY-14643 for 48 hours (n =5). **b**, Transcript copy number in livers of mice fasted for 24 hours (n = 5). Each data point represents the mean ± SD. Adjusted *p* values, provided in the panels, as determined by *t*-test.

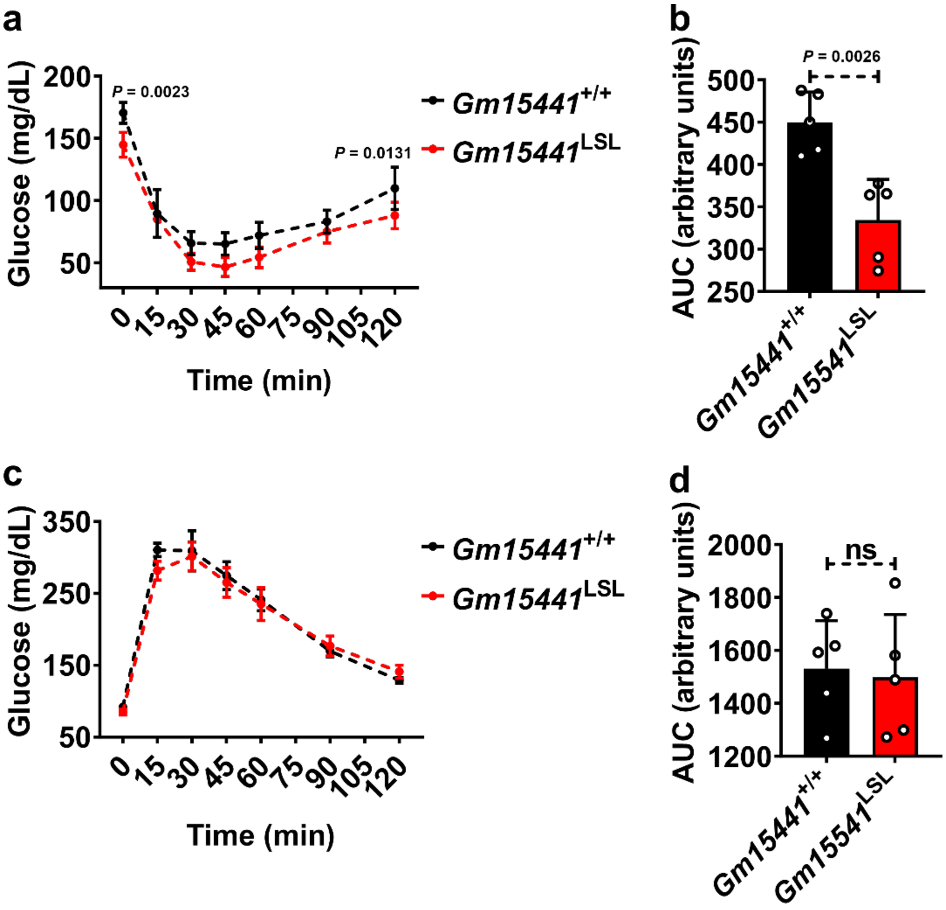

Supplementary Figure 7. Insulin (ITT) and glucose (GTT) tolerance tests in *Gm15441*$^{+/+}$ and *Gm15441*^{LSL} mice.

a, Insulin tolerance test (ITT) results in *Gm15441*$^{+/+}$ and *Gm15441*^{LSL} mice. **b**, Calculated area under the curve (AUC) for ITT tests (n = 5). **c**, Glucose tolerance test (GTT) results in *Gm15441*$^{+/+}$ and *Gm15441*^{LSL} mice. **d,** Calculated AUC for GTT tests (n = 5). Each data point represents the mean ± SD ns, not significant Adjusted *p* values, provided in the panels, as determined by ANOVA with Tukey's multiple comparison correction, two-sided for comparisons to *Gm15441*$^{+/+}$ and *Gm15441*^{LSL} mice.

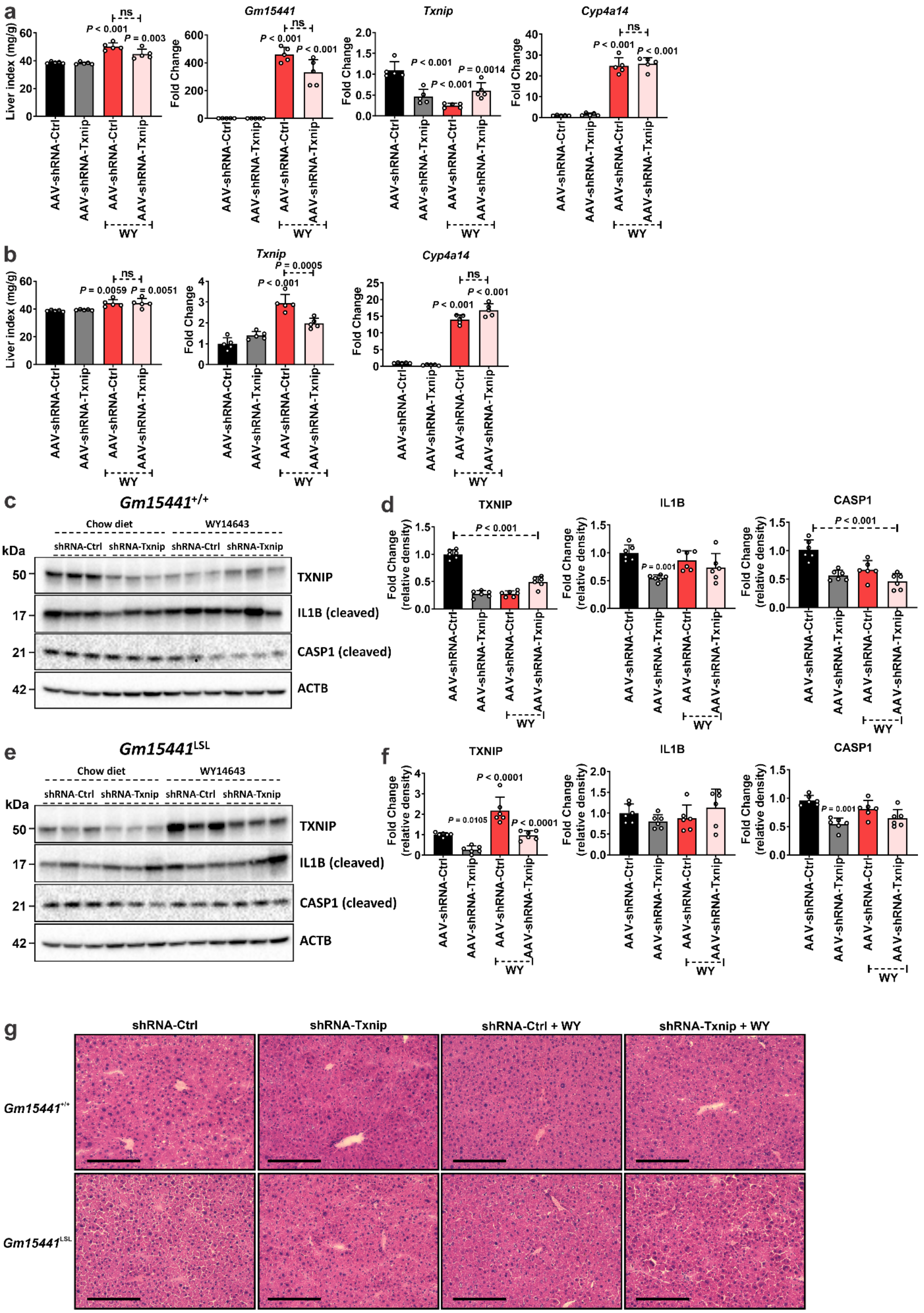

a
Gm15441
Txnip
Cyp4a14
Liver index (mg/g)
Fold Change
AAV-shRNA-Ctrl
AAV-shRNA-Txnip
WY
ns
P < 0.001
P = 0.003
b
P = 0.0059
P = 0.0051
P = 0.0005
P = 0.0014
c
Gm15441+/+
Chow diet
WY14643
shRNA-Ctrl
shRNA-Txnip
kDa
TXNIP
IL1B (cleaved)
CASP1 (cleaved)
ACTB
d
IL1B
CASP1
Fold Change (relative density)
e
Gm15441LSL
f
P < 0.0001
P = 0.0105
g
shRNA-Ctrl + WY
shRNA-Txnip + WY

Supplementary Figure 8. Knockdown of TXNIP attenuates PPARα-induced NLRP3 inflammasome activation in *Gm15441*$^{+/+}$ mice.

a, Liver indices (mg liver/g body mass; a measure of hepatomegaly) from AAV-shRNA-Ctrl or AAV-shRNA-Txnip injected mice treated with the PPARα agonist WY-14643 and quantitation of *Gm15441* RNA and *Txnip* and *Cyp4a14* mRNAs in *Gm15441*$^{+/+}$ mice (n = 5). **b**, Liver indices (mg liver/g body mass) from AAV-shRNA-Ctrl or AAV-shRNA-Txnip injected mice treated with the PPARα agonist WY and quantitation of *Gm15441*, *Txnip*, and *Cyp4a14* in *Gm15441*^{LSL} (n = 5). **c**, Western blotting of TXNIP, CASP1 (cleaved), and IL1B (cleaved) from whole liver extracts in *Gm15441*$^{+/+}$ mice. **d**, Densitometric analysis of TXNIP, CASP1 (cleaved), and IL1B (cleaved) protein levels from whole liver extracts in *Gm15441*$^{+/+}$ mice (n = 6). **e**, Western blotting of TXNIP, CASP1 (cleaved), IL1B (cleaved) from whole liver extracts in *Gm15441*^{LSL} mice. **f**, Densitometric analysis of TXNIP, CASP1 (cleaved), and IL1B (cleaved) protein levels from whole liver extracts in *Gm15441*^{LSL} mice (n = 6). **g**, Representative H&E staining of liver tissue from *Gm15441*$^{+/+}$ and *Gm15441*^{LSL} mice treated with WY-14643 for 24 hours (n = 5, 3 images/mouse). Scale bars represent 100 μm (200X). At least five mice were analyzed from each group. Each data point represents the mean ± SD. Adjusted *p* values, provided in the panels, as determined by ANOVA with Tukey's multiple comparison correction, one-sided for comparisons to AAV-shRNA-Ctrl or as shown (dashed horizontal lines).

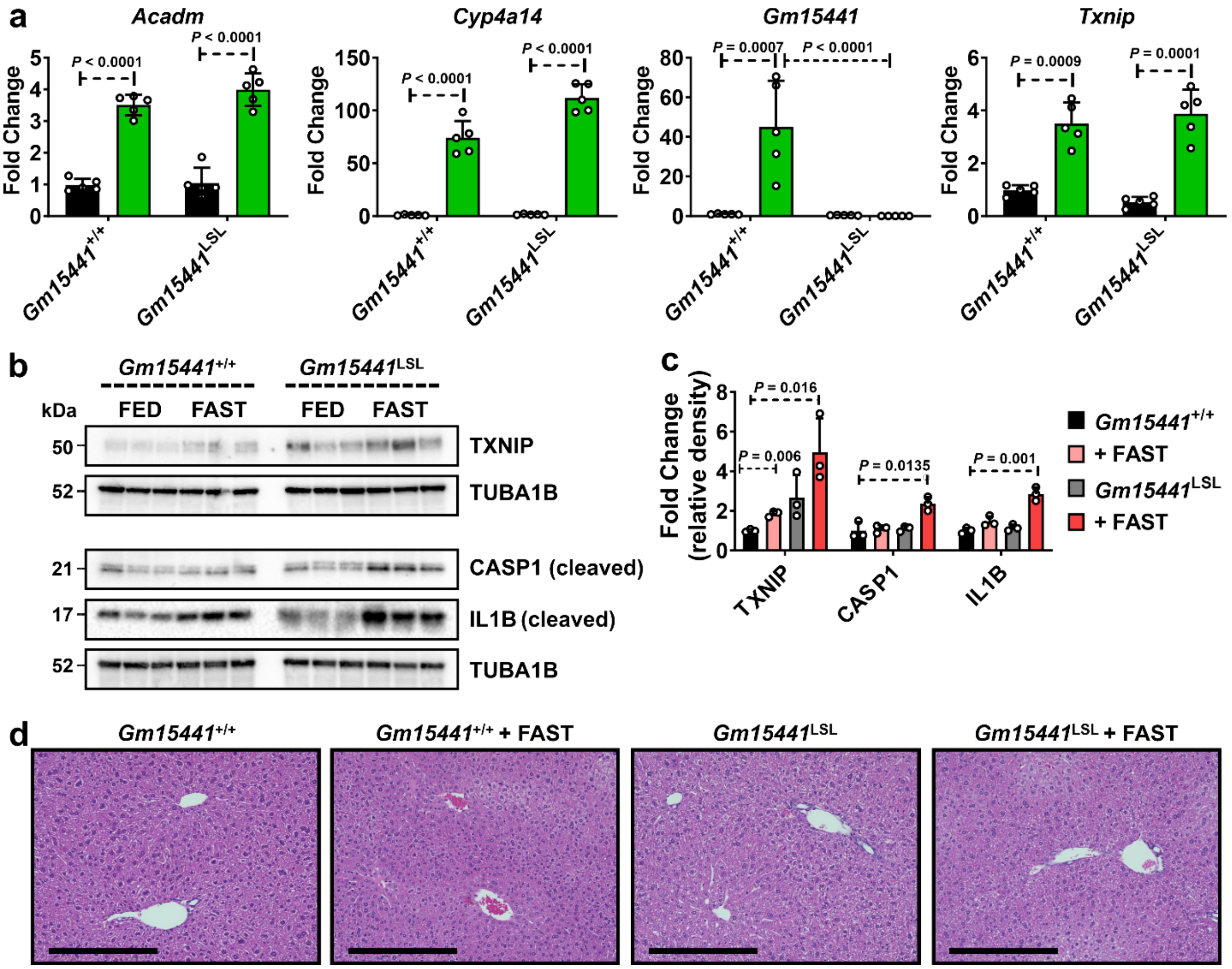

Supplementary Figure 9. Loss of *Gm15441* potentiates inflammasome activation during physiological response to acute fasting.

a, Analysis of *Acadm*, *Cyp4a14*, and *Txnip* mRNA and *Gm15441* RNA expression from livers of *Gm15441*$^{+/+}$ and *Gm15441*^{LSL} mice fed or fasted for 24 hours as determined by qRT-PCR (n = 5). **b**, Analysis of TXNIP, CASP1 (cleaved), and IL1B (cleaved) protein expression from livers of fed and fasted *Gm15441*$^{+/+}$ and *Gm15441*^{LSL} mice. **c**, Densitometric analysis of TXNIP, CASP1 (cleaved), and IL1B (cleaved) protein expression (n = 3). **d**, Representative H&E staining of liver tissues from fed or 24 h fasted *Gm15441*$^{+/+}$ and *Gm15441*^{LSL} mice (n = 5, 3 images/mouse). Scale bars represents 100 nm (200x). Each data point represents the mean ± SD. Adjusted *p* values, provided in the panels, as determined by ANOVA with Tukey's multiple comparison correction, two-sided for comparisons to fast-stimulated and unstimulated livers of the same genotype, or as shown (dashed horizontal lines).

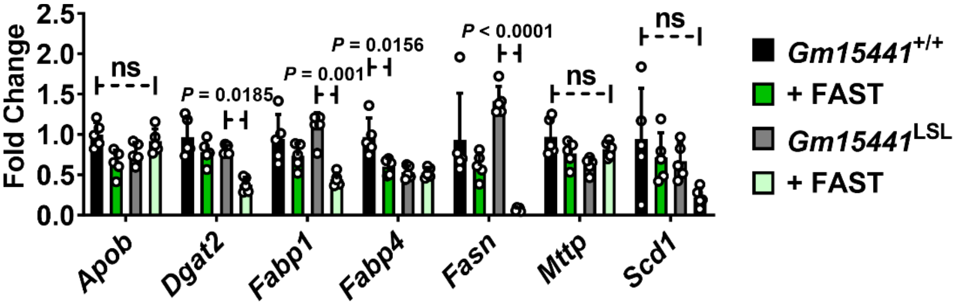

Supplementary Figure 10. Lipid metabolism-related gene expression in *Gm15441*$^{+/+}$ and *Gm15441*^{LSL} mice under fed or fasting conditions.

Hepatic expression of lipid metabolism-related genes (*Apob*, *Dgat2*, *Fabp1*, *Fabp2*, *Fasn*, *Mttp*, and *Scd1*) in *Gm15441*$^{+/+}$ and *Gm15441*^{LSL} mice fed or fasted for 24 hours as determined by qRT-PCR. Each data point represents the mean ± SD for n = 5 liver samples. Adjusted *p* values, provided in the panels, as determined by ANOVA with Tukey's multiple comparison correction, two-sided for comparisons between fast-stimulated and unstimulated livers of the same genotype, or as shown (dashed horizontal lines). ns, not significant.

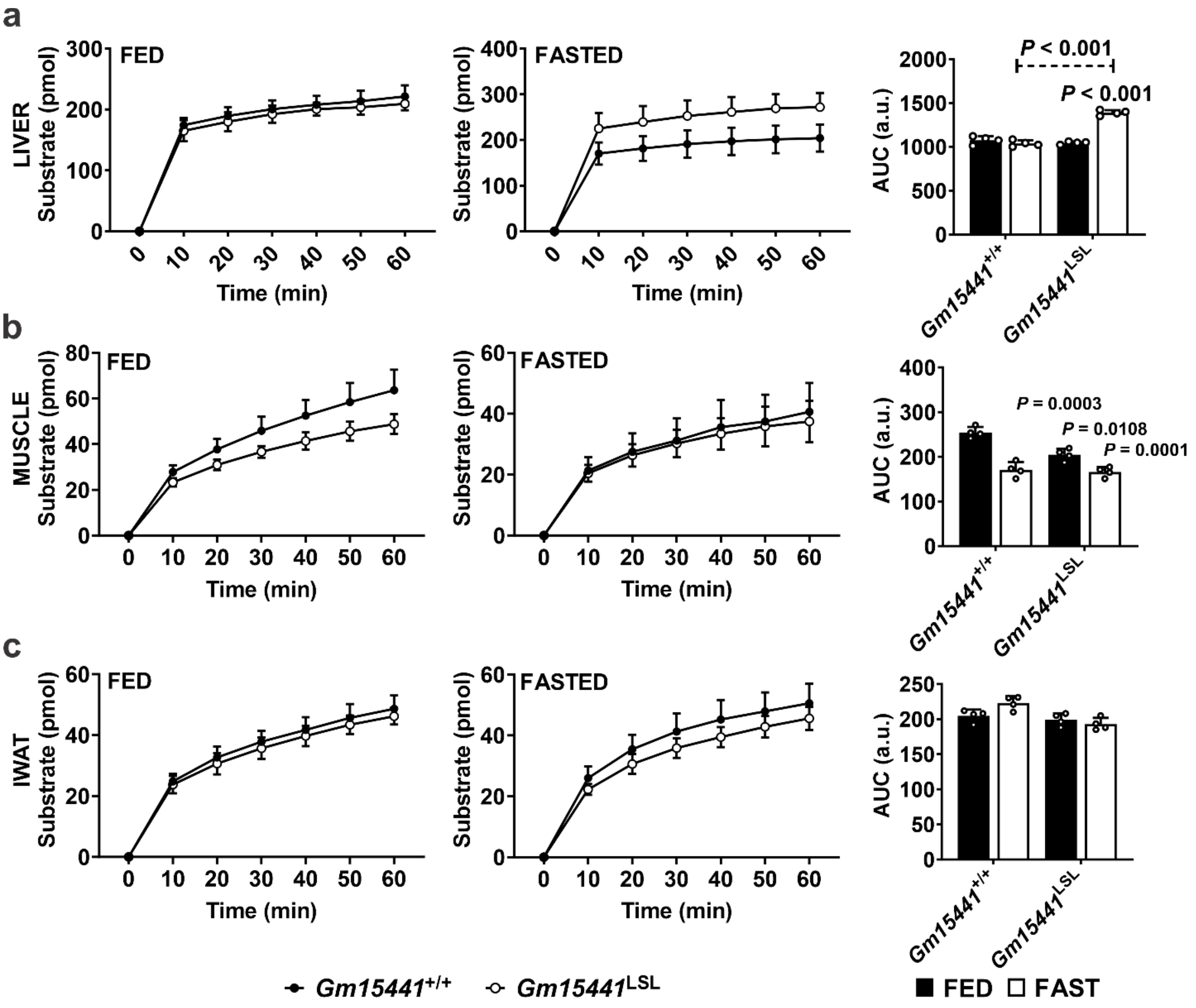

Supplementary Figure 11. Total lipase activity in *Gm15441*$^{+/+}$ and *Gm15441*^{LSL} mice under fed or fasting conditions.

Gm15441$^{+/+}$ or *Gm15441*^{LSL} were fed or fasted for 24 hours, and lipase activity was measured in freshly isolated tissues. Lipase activity from (**a**) liver, (**b**) skeletal muscle, and (**c**) inguinal white adipose tissue (IWAT) from *Gm15441*$^{+/+}$ and *Gm15441*^{LSL} mice. Area under the curve (AUC) was calculated and tested for significance. Data are mean ± SD (n = 4). Asterisks above bars indicate significance versus fed group of the same genotype. a.u., arbitrary units. Adjusted *p* values, provided in the panels, as determined by ANOVA with Tukey's multiple comparison correction, two-sided for comparisons to *Gm15441*$^{+/+}$ or as shown (dashed horizontal lines).

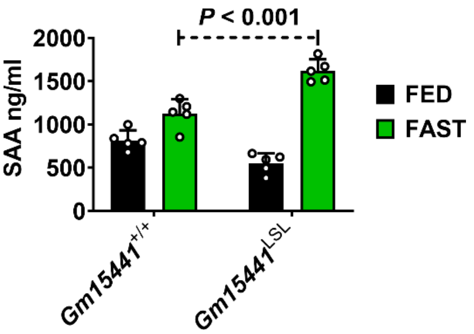

Supplementary Figure 12. Plasma SSA levels in fasted *Gm15441*$^{+/+}$ and *Gm15441*^{LSL} mice. Mice were fasted for 24 hours then plasma SSA levels were measured by ELISA (n = 5). Adjusted *p* values, provided in the panels, as determined by ANOVA with Tukey's multiple comparison correction, two-sided for comparisons between fast-stimulated and unstimulated livers of the same genotype, or as shown (dashed horizontal lines).

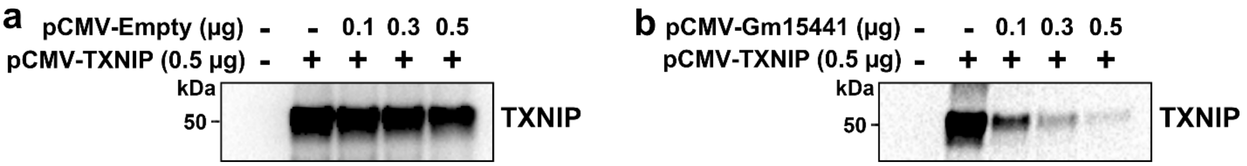

Supplementary Figure 13. Gm15441 represses Txnip translation.

Reticulocyte-based transcription and translation assays were performed using pCMV-Txnip expression plasmid (0.5 μg) in combination with increasing amounts (0.1, 0.3, or 0.5 μg) of either pCMV-Empty or pCMV-Gm15441 expression vectors. **a,** Western blot analysis of TXNIP protein expression from pCMV-Txnip plasmid with increasing concentrations of pCMV-Empty plasmid (negative controls) or **b,** increasing concentrations of pCMV-Gm15441 expression plasmid.

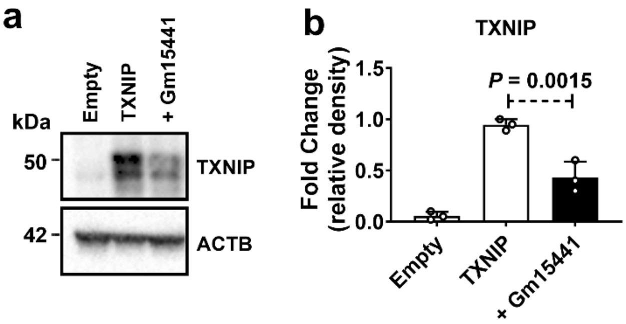

Supplementary Figure 14. Effect of *Gm15441* expression on TXNIP protein levels in Hepa-1 cells.

Hepa-1 cells were co-transfected with a TXNIP expression vector and either an empty control plasmid or Gm15441-expressing plasmid. **a,** Western blotting of TXNIP and ACTB in whole cell lysates from Hepa-1 cells. **b,** Densitometric analysis of TXNIP protein expression. Each data point represents mean ± SD (n = 3). Adjusted *p* values, provided in the panels, as determined by ANOVA with Tukey's multiple comparison correction, one-sided for comparisons as shown (dashed horizontal line).

Supplementary Table 1. Pathway analysis of upregulated genes from WY-14643 treated mice liver.

	Term	Gene Counts	P-Value
**1**	GO:0006629~lipid metabolic process	79	4.11E-26
**2**	GO:0006635~fatty acid beta-oxidation	22	1.12E-17
**3**	Peroxisome	36	5.49E-23
**4**	DNA replication	28	2.62E-16
**5**	DNA repair	35	6.71E-09
**6**	Cell cycle	68	1.62E-13

DAVID pathway analysis for genes up regulated by WY-14643 treatment in mouse liver. Shown is the overall enrichment score for each cluster, and the gene count, gene list, and False Discovery Rate (FDR) for the top term in each cluster, as determined by DAVID analysis using default conditions.

Supplementary Table 2. Pathway analysis of downregulated genes from WY-14643 treated mouse liver.

	Term	Gene Counts	P-Value
**1**	Secreted	133	3.96E-24
**2**	Monooxygenase	32	6.02E-19
**3**	Immunity	44	1.19E-12
**4**	Serine protease inhibitor	17	4.88E-09
**5**	mmu00980:Metabolism of xenobiotics by CYPs	16	5.07E-08
**6**	GO:0009615~response to virus	15	1.10E-06

DAVID pathway analysis for genes down regulated by WY-14643 treatment in mouse liver. Shown is the overall enrichment score for each cluster, and the gene count, gene list, and False Discovery Rate (FDR) for the top term in each cluster, as determined by DAVID analysis using default conditions.

Supplementary Table 3. Assembled boundary sequence for exon 2/3-specific 5' RACE products

5' Product*[#]
CACTTTGGAGWGTTGCAGCCMGGWGGATGGTCTGATCTTCTGCACTCTGAGGCTCTTGCM<br>CGCCACGATGCGCAAGTCCCTGAGATAATGTGATTGCCTCTGACTGAGGACAGCTTCTGA<br>GTGAACACTTTGGTCTGGCCATTGGCAAGGTGAAAGAAAAATGTGCTTAGGAGAGGATTT<br>CATGACTCGCCTGAGCCAGCGCAATTCATTTCGAWATTACCCGTCCGGCCACCTTCTCCCC<br>GCTGCCGTACACCTTCTCGGGGTCSTTGAAGACCACCTCAAAAGASTTGATCTTCTTGARC<br>ATCACCATGATTGAGCCKAGTGGGTTCARGAAAAACGAAAGCCGGAGAGGAAAAAAATC<br>TTTAAKAACCCTTCAAAGCAAACGAAGAGCTCGCCTCCGTAAAGTCAGGGCTTGAAAACC<br>CAAACGAAAATGTAAAGAAAGCTTCAAACAAACCCTAACAACTTTTCCAAAAGGTGCCTG<br>GAAGTTTCAGCCTGCCGGCTGTGCCAAAAGCTTTCAGGAGAAAAATAACAGCTGTCTTCC<br>GCAACTGGAGAAACTCTGGAAACCCCGAGACCAGAGGAGGAGAGTGTCAAGCGGCTGCC<br>GGAAACGGCTTATATAGCCGCCTGGCTTGGCGCTCCACGCGAGTGCTGGCCTGGAGGCTC<br>GTGCAGCCCTCGTGCACAGTTCTCCCATTGGCTACTTGGCCCTTGTTTACCAGGCCTCCAA<br>CCAATCAGCGAGGCCGCCGGGGCGCGTGGACACGGTGTGCTCCTAGCGGGGAAAATGGTT<br>GTTGTTCGGGTGCGCAGCC

* K = G or T; M = A or C; R = A or G; S = G or C; and W = A or T

[#] Exon boundary denoted by red line

Supplementary Table 4. Densitometry analysis of CASP1 (cleaved) protein bands.

Gm15441$^{+/+}$							
			ACTIN	CASP1	Normalize	Average	Final
Chow	shRNA-Ctrl	1st	4106.397	11157.46	2.717092	2.146813	1.26564
			5266.033	10703.95	2.032639		0.946817
			5217.79	8821.752	1.690707		0.787543
		2nd	17628.94	31029.61	1.760152	1.673948	1.051498
			18889.4	31341.66	1.65922		0.991202
			19632.52	31460.54	1.602471		0.9573
	shRNA-Txnip	1st	5149.376	6796.61	1.31989		0.614814
			6704.497	7636.196	1.138966		0.530538
			7382.79	7563.296	1.02445		0.477196
		2nd	24504.59	23367.62	0.953602		0.569672
			25752.35	21733.26	0.843933		0.504157
			26776.47	21798.62	0.814096		0.486333
WY14643	shRNA-Ctrl	1st	6452.79	8705.489	1.349105		0.628422
			5784.134	11004.97	1.902614		0.886251
			6183.962	10039.44	1.623464		0.756221
		2nd	21879.16	21838.87	0.998158		0.59629
			19861.82	21413.89	1.078143		0.644072
			19458.04	21262.74	1.092749		0.652797
	shRNA-Txnip	1st	6853.447	9193.146	1.34139		0.624829
			6169.255	6990.66	1.133145		0.527827
			5579.447	6869.317	1.231182		0.573493
		2nd	22633.49	20432.74	0.902766		0.539304
			21282.84	19513.97	0.916887		0.54774
			21810.47	19924.72	0.913539		0.545739
Gm15441^{LSL}							
			ACTIN	CASP1	Normalize	Average	Final
Chow	shRNA-Ctrl	1st	2459.406	8789.782	3.573945	4.085553	0.874776
			2371.456	10667.39	4.498244		1.101012
			2417.406	10115.56	4.184469		1.024211
		2nd	16508.62	23370.74	1.415669		0.845707
			17673.16	26231.3	1.484245		0.886674
			18274.11	22384.16	1.224911		0.73175
	shRNA-Txnip	1st	3577.406	8345.296	2.332779		0.570982
			2777.113	7812.296	2.8131		0.688548
			3470.87	7326.539	2.110865		0.516666
		2nd	22636.16	20352.21	0.899102		0.537115
			17984.21	18434.48	1.025037		0.612347
			22361.94	20959.74	0.937296		0.559931
WY14643	shRNA-Ctrl	1st	2767.577	9586.924	3.464013		0.847869
			2821.698	8808.61	3.121741		0.764093
			3108.406	10371.75	3.336679		0.816702
		2nd	16338.43	28465.01	1.742212		1.04078
			18994.16	26360.01	1.387796		0.829056
			20773.38	22065.74	1.062213		0.634555
	shRNA-Txnip	1st	4371.062	8933.56	2.043796		0.50025
			2431.698	9179.267	3.774838		0.923948
			3694.477	8525.953	2.307756		0.564858
		2nd	25315.77	26163.57	1.033489		0.617396
			22207.64	25128.77	1.131537		0.675969
			27453.61	24328.18	0.886156		0.529381

Supplementary Table 5. The list of differential gene expression analysis of RefSeq genes from *Gm15441*$^{+/+}$ (WT) and *Gm15441*^{LSL} (KO) mouse liver.

	Probe ID	WT+Wy/WT	KO+Wy/KO	KO/WT	KO+Wy/WT+Wy
1	**GM15441**	UP	0	Up	Down
2	**TXNIP**	Down	Up	0	Up
3	CYP2A22	0	Up	0	Up
4	DAB1	0	Up	0	Up
5	PGLYRP1	0	Up	0	Up
6	CENPI	0	Up	0	Up
7	DTL	0	Up	0	Up
8	MCM5	0	Up	0	Up
9	MCM6	0	Up	0	Up
10	TRHDE	0	Up	0	Up
11	MCM3	0	Up	0	Up
12	IGSF23	0	Up	0	Up
13	SAA2	0	Down	0	Down
14	SAA1	0	Down	0	Down
15	LCN2	0	Down	0	Down
16	TFF3	0	Down	0	Down
17	ORM2	0	Down	0	Down
18	BHMT	0	Down	0	Down
19	AA465934	0	0	0	Up
20	TSPYL5	0	0	0	Up
21	GPR133	0	0	0	Up
22	PCSK9	Down	0	0	Up
23	AACS	Down	0	0	Up
24	HYLS1	Down	0	0	Up
25	MUP9	Down	0	Up	Up
26	SNTG1	0	Down	Up	0
27	FAM84B	0	Down	Up	0
28	GM5801	0	Down	Up	0
29	LOC100048884	0	Down	Up	0
30	ABHD1	Up	0	Up	0
31	APOA4	0	0	Up	0
32	WDR72	0	0	Up	0
33	A730098P11RIK	0	0	Up	0
34	CYP2A4	Up	Up	Up	0
35	UGT1A9	Up	0	Up	0
36	COX6B2	Up	Up	0	Up
37	SELENBP2	Down	Down	0	Up
38	HSD3B5	Down	Down	0	Up
39	BC018473	0	0	Up	Up
40	CTSE	Up	Up	Up	Up
41	NNMT	Down	Down	0	Down
42	FOXQ1	Down	Down	Down	0

Supplementary Table 6. Gm15441 donor plasmid sequence.

GAGCTCAATGCCATTTTTCAGTCACAGTTTGATGGCTCCACAGCACAAGGTTCACA
CAGAATGTCTGTTGTCACAGAAAACGCCTTAGTCCAGGTGTGCTGAGTGACACCT
ATAAACCCAGTGCTCAGAGCTTGGGGCCCCCCGTGCAGAGTGAGGTCCAGGCCAG
CTTGGGTGACAGAGAGAGACCAGTCTCGGAAAGGAAATAAAATCTTGTCGATAGT
GCTAGATGTGGTAGGGCTGGGGCTGGGGCTGGGATACCAGGAGAGGGGGCATAT
GTTCTGCCTCTTGCAGGATCAAATCTCTTGAGTGGCCTTGGGTGTTGTTATCTGTCC
TTCCCTGGCCACTCAGTTCTCATAACAAGTCTGTGTACACAAAGATGCAGACCTTC
CTGGTCTCTCCATCCCTTAGCCTGGCCCTCTTTCTCATTCTTGTCCCTGCTCCAACC
CTCAAGTCCCTGGAAACCCAGCTGCATAAAAAACTTTTCCGTCAACTTTGACCTAG
TTTCCTGATCGCAGCATTAAAGGGGAGGGGCTACTAAATGTTTGAACAGGTGAGA
AATGGGGGAGGACTTCAGGTGAGAGTTTTCACAGCAATGGGGAGAGGTAGGTAC
CAGCACACTGTAAACAGACAAAAGGATCTCATGAATCTAAGATGCAGATCAGTTG
GCAAAGTGTTTGCCCAGTGTAGACCGAGCCCGATTTCAGCCTCCAGCAGTGCATA
AAGTGAGCGTGGAAACACTCGAATGCCTATGAATCCAGTGCTAGGAAGGTGGGGC
CAGAGGGCCAGAAATTCAAGGTCACCCTGAACTCGAGATAACTTCGTATAGCATA
CATTATACGAAGTTATCTGACCTCTTCTCTTCCTCCCACAGGACAAACTTGTTTATT
GCAGCTTATAATGGTTACAAATAAAGCAATAGCATCACAAATTTCACAAATAAAG
CATTTTTTTCACTGCATTCTAGTTGTGGTTTGTCCAAACTCATCAATGTATCTTAAA
CTTGTTTATTGCAGCTTATAATGGTTACAAATAAAGCAATAGCATCACAAATTTCA
CAAATAAAGCATTTTTTTCACTGCATTCTAGTTGTGGTTTGTCCAAACTCATCAAT
GTATCTTAAACTTGTTTATTGCAGCTTATAATGGTTACAAATAAAGCAATAGCATC
ACAAATTTCACAAATAAAGCATTTTTTTCACTGCATTCTAGTTGTGGTTTGTCCAA
ACTCATCAATGTATCTTAGCATGGACGTCATAACTTCGTATAGCATACATTATACG
AAGTTATAGACACAGCCTAGACTAAATGAAACCCAGTATCAAAAGACAAAGAAG
GAAAAATGTCACCTGGCTTGGATAAAGGCACAGGCTGTGAGCTTGGTGACCTGTG
TCTACAGAAGTCCTCTGACCTCCTGGGCACTGTGGTATACAGACACAAATGATTGT
TCTTTTTATTTATTTATGTTTTATTGTTTATTTTATTTTTTGTTTGTTTGTTTTTGTTTT
TTTGAGACAGGGTTTCTCTGTGTAGCCCTGGCTGTCCTGGAACTCACTTTGTAGAC
CAGGCTGGCCTTGACCTTAGAAATCTGCCTGCCTCTGCCTCCCGAGTGCTGGGATT
AAAGGCGTGCGCCAGTGAGGCAGGAAAGTGACAGGTGCGCTATAGCGAATGAAT
GATAAAGATCAATTACTAGCGCAGAAAAGGATTTCTTTTTTTTTCAATTTCTGTTC
AGTTCTGTGTTCACAGCTTTTTTTGTGTGATGTGTGGTTTTGTGGTTTGTTTGGGGG
ATAAAAGTGCTTGCTCTGTAGCCCTAGCAGACCTGGCGGTACCTGTGGCCAAGGC
TGTCTTGAATTCTTGGCAATCCTCCTGCTTTTGCCTCTCAAATGCTGGGTTTTAGGT
GTGCATGACCACAGCGAGCCTCCCCTTGGTTTCTAATACAGTGCTTCAGAGAGTGT
ACTGGTGTGTGGAGAGCTATGGAAAACCCATTTTGACGAAAAACATTTAGATGAA
CCAAAAACCAGAAAGAATGGTGTGATGTATTCTGCCATCTTTGTTTCTCATGTGTG
TAGCTTTTTCCTGAACAAAATAACTGTCGAC
7-806: Left homology arm; 1288-2087: Right homology arm; 813-846 and 1253-1286: LoxP; 813-1286: Floxed STOP

Supplementary Table 7. List of primer sequences used in qPCR, sequencing, and cloning.

**qPCR primers**			
**Gene symbol**	**Gene name**	**Forward sequence**	**Reverse sequence**
Abcc6	ATP-binding cassette, sub-family C, member 6	TGGTATTCGTGGCTGCTACA	GCAGAGTCTGTGTCACCTGGA
Acacb	acetyl-Coenzyme A carboxylase beta	CAGGTCCTCCCTGCTGATTC	TCGGGCTACCTTGAGAGCAT
Acadm	acyl-Coenzyme A dehydrogenase, medium chain	GAACCAGACCTACAGTCGCA	GCTCCACTAGCAGCTTTCCA
Acox1	acyl-Coenzyme A oxidase 1	GGGCACGGCTATTCTCACAG	CATCAAGAACCTGGCCGTCT
Acsl1	acyl-CoA synthetase long-chain family member 1	CGATGGCTGTTGGACTTTGC	CACCCAGGCTCGACTGTATC
ApoC3	apolipoprotein C-III	TCTGCCCGAGCTGAAGAGGTA	CGCTACTTAGCGCATCCTGG
Cbfa2t3	CBFA2/RUNX1 translocation partner 3	CGGCCCCTGGCCATACC	CACCATGTCCATGATGCAGTTC
Cyp2c54	cytochrome P450, family 2, subfamily c, polypeptide 54	CTAATTAAAGGAGCCCAGGAAGA	CATTGACTCTGTCCCACCAA
Cyp4a10	cytochrome P450, family 4, subfamily a, polypeptide 10	AAGGGTCAAACACCTCTGGA	GATGGACGCTCTTTACCCAA
Cyp4a14	cytochrome P450, family 4, subfamily a, polypeptide 14	AGCAAACTGTTTCCCAATGC	ACCCCTCTAGATTTGCACCA
E11Rik	RIKEN cDNA 4931417E11 gene	CCGAGCGCTTTGAAGAATTTTG	TTGTCCGTTACTACATCCGGG
Fabp1	fatty acid binding protein 1	AGTCAAGGCAGTCGTCAAGC	ATGTCGCCCAATGTCATGGT
Fbp1	fructose bisphosphatase 1	GCACAGCTCTATGGTATCGCT	CACAGGTAGCGTAGGACGAC
Fasn	fatty acid synthase	GTGATAGCCGGTATGTCGGG	TAGAGCCCAGCCTTCCATCT

Gm15441	predicted gene 15441	ATCCGGACATCAAGGAGTATTCG	TTATCTCAGGGACTTGCGCATC
Il22ra1	interleukin 22 receptor, alpha 1	TGACTATGGAGACCCGCAAC	GGTTTGATGGTAGTGTGCTGC
Neg	Geneblock of negative control	TGAATGAACCGCCTACGGTG	AATGCAGGCCCTGAAACCAA
Pck1	phosphoenolpyruvate carboxykinase 1	CTGAAGGTGTCCCCCTTGTC	GATCTTGCCCTTGTGTTCTGC
Pdk4	pyruvate dehydrogenase kinase, isoenzyme 4	TTCCAGGCCAACCAATCCAC	TGGCCCTCATGGCATTCTTG
Pklr	pyruvate kinase liver and red blood cell	CTTCCCCTTGCTCTACCGTG	AGCCACGAAGCTTTCCACTT
Ppara	peroxisome proliferator activated receptor alpha	AGAGCCCCATCTGTCCTCTC	ACTGGTAGTCTGCAAAACCAAA
Ppp1r3c	protein phosphatase 1, regulatory subunit 3C	GAGCTGCACCAGAATGATCC	AGGTGGTGAATGAGCCAAGC
Pygl	liver glycogen phosphorylase	TGTGGCAGAAGTGGTGAACA	GGCTGGAATGACTTTTTCGGC
Slc2a2	solute carrier family 2, member 2	TCCCTTGGTTCATGGTTGCT	CCCAAGGAAGTCCGCAATGT
Txnip	thioredoxin interacting protein	GAAGGCTTTTCTCGATCGCC	CTGGTGCCATTAGGTCAGGG
XLOC 009018	ncRNA_inter_chr16_13170	ATTAAATCAGCATTGTCTGCTGGA	AGTGTTCACCTTCAGTTTGCCA
XLOC 011646	ncRNA_inter_chr19_15132	TCATGGTGGACAGGAGGTTTTC	GCTGCATGGTTCTTCCTCCATA
XLOC 013968	ncRNA_inter_chr2_1830	CCATGAGTAACTCCACTTCTGGAT	GTGTGCACATTAGGTGGCAG
XLOC 016119	ncRNA_inter_chr4_3651	CTCTCACCTGCAAGGTCTAGATT	GCTAGGATATCTCTGCATTCCCC
XLOC 020538	MTR034310	TTCCTGGAGGAAAACAGGAGC	TGAGAACTGGGGATGCTGGTA
XLOC 023573	ncRNA_as_chr8_7528	CCCACCTGACCTGTCTAGC	AATAAGACCCTGCTTCGTGGTTT

XLOC 024310	ncRNA_inter_chr9_8249	GCCCTAACTTCCCACCTTCAC	GGTCTTTGGTTCATCATCCATCC
XLOC 024748	ncRNA_inter_chr9_7907	CTATCACCGGACAGTTTAACAGC	CTGTGGGGGATCCAGTTCATTA
XLOC 024896	ncRNA_inter_chr9_8081	GGTATGGTTGTAGCCATAGTGGT	TCCATGCGTTCAGAATCACCTTTA
**Genotyping primers**			
**Gene name**		**Forward sequence**	**Reverse sequence**
Gm15441 Lox-STOP-Lox (LSL)		TGCGAGGCACGATATGGCGA	AGCGCACCTGTCACTTTCCTGC
**ChIP Primers**		**Forward sequence**	**Reverse sequence**
Geneblock A	PPRE reporter construct geneblock A	TACAATCCACTTGCAGAGGTCAA	ATGAAGTGTAGTAAGGGAAGGGCAG
Geneblock B	PPRE reporter construct geneblock B	GCATAGAGAGCTCAGGTGCC	TCCAAACCTCCATAGACCAGTG
Geneblock C	PPRE reporter construct geneblock C	CAACGAAAGCCTGTGAGGTTG	GACAGGGTCTCTTACAGCTCA
Geneblock D	PPRE reporter construct geneblock D	ACTCCAGACGTTTGCTTGGT	TGGGGATCAGTTGTGCTGTC
Geneblock E	PPRE reporter construct geneblock E	TGGATGCAGCAAATTTAGGTTGA	CCTTTCCCTTACCAAACCGC
Geneblock F	PPRE reporter construct geneblock F	AGTGGAGGGGAGACACCATC	CCCACACACGCCATTTTGTA
**5'RACE primers**			
**Forward sequence**			
TCATACACATACGATTTAGGTGACACTATAGAGCGGCCGCCTGCAGGAAA			
**Reverse sequence**			
CGAAATGAATTGCGCTGGCTCAGGC			

Supplementary Table 8. Gm15441 target sequence used for fluorescence in situ hybridization probes*

GGAGCAAGCCGATAAGCAGCACCCCTCCATGGCCTCTGCATCAGCTCCTGCCTCC
AGGTTCCTGCCCTTGCCTTCCCTCACTGATGGACTGTGATCCGGACATCAAGGAGT
ATTCGACTTTGAGGATGTTGCAGCCCAGGATGGATGGTCTGATCTTCTGCACTCTG
AGGCTCTTGCCACGCCACGATGCGCAAGTCCCTGAGATAATGTGATTGCCTCTGAC
TGAGGACAGCTTCTGAGTGAACACTTTGGTCTGGCCATTGGCAAGGACCTTGGCC
ACGCCGCAAGCCAGGATCCTGACGGCTTTGACTCGGGTAACTTCACACACTTCCA
CTATTACCCGTCCGGCCACCTTCTCCCCGCTGCCGTACACCTTCTCGGGGTCGTTG
AAGACCACCTCAAAAGACTTGATCTTCTTGAACATCACCATGATTGAGCCGAGTG
GGTTCAAGAAAAACGAAAGCCGGAGAGGAAAAAAATCTTTAAGAACCCTTCAAA
GCAAACGAAGAGCTCGCCTCCGTAAAGTCAGGGCTTGAAAACCCAAACGAAAAT
GTAAAGAAAGCTTCAAACAAACCCTAACAACTTTTCCAAAAGGTGCCTGGAAGTT
TCAGCCTGCCGGCTGTGCCAAAAGCTTTCAGGAGAAAAATAACAGCTGTCTTCCG
CAACTGGAGAAACTCTGGAAACCCCGAGACCAGAGGAGGAGAGTGTCAAGCGGC
TGCCGGAAACGGCTTATATAGCCGCCTGGCTTGGCGCTCCACGCGAGTGCTGGCC
TGGAGGCTCGTGCAGCCCTCGTGCACAGTTCTCCCATTGGCTACTTGGCCCTTGTT
TACCAGGCCTCCAACCAATCAGCGAGGCCGCCGGGGCGCGTGGACACGGTGTGCT
CCTAGCGGGGAAAATGGTTGTTGTTCGGGTGCGCAGCCTTGCCAGCAGGAGGGGC
GGGGCCCGGGGCGACGGGTGCCCTCCCTCCCCCCGGGAGGCTGGGACCCCGGGAC
CCTGAACCTCATCCAGGGGACTCCTCTTGGGAGCTGGGTTATTTGGGTGTGTGCTT
TGTTTTTTTTTCCCCCTTCTTTTTTTAGGTGAAAGAAAAATGTGCTTAGGAGAGGA
TTTCATGACTCGCCTGAGCCAGCGCAATTCATTTCGAGGTGACCATTTATTCCTGG
CTCACAAGTTCGCAAGTCTTGTGAAAAACAATCCCATCGCACCGAGGATACTCCA
GGATGGAGCGGAGGGCGAGAGTGCAGTCGTGTTAGTGAGAAAGAGCCCACCCGA
TATGCTCCTATTGCACTTCTATTGAAGAATAAAAGGCTGCGGTCTCTCCAGACGAG
AACTTGTCAGATATTTTAAAGTCTGGCTTTCAGTCCTTTCTGGTGAATGCTCTGGTG
TCCGCCGATGCCTTAGAAGTGGAGTTAAGAGTTGTGAGCTGCCGTTTTTTGGTTCT
GGGACTCGAACTCGTTTCCTCTGATACTATCAACCACCAAGCCATCTCTTCAGCCC
CAGCTACATAAGACTTCAGGAGAACCAGAGAGATGGTGCCAGGCCTGCCAACCTG
AGTTCAATAGCAGAACCCACACAGTAGCAAAAGAACCAACTCTGACCTCCCCTCT
CCCCCTCCCCTCTCCTTCCCCTCTCCAGTAGTCACTCCAGAGACACCCAAAATAAA
AAATTTAAATGT

* Red text denotes sequence that is antisense to *Txnip* and excluded as a target
