# Supplementary material for: Long non-coding RNA Gm15441 attenuates hepatic inflammasome activation in response to PPARA agonism and fasting
Source: Nat Commun. 2020 Nov 17;11:5847. doi: 10.1038/s41467-020-19554-7 (PMC7673042; doi:10.1038/s41467-020-19554-7)
Supplement: Supplementary file 2 — Description of Additional Supplementary Files [file 41467_2020_19554_MOESM2_ESM.docx]

Description of Additional Supplementary Files

File Name: Supplementary Data 1

# Description: Differential expression analysis of RefSeq genes. WY, WY-14643; Resp, Response; WT, *Ppara*^+/+^; KO, *Ppara*^-/-^

File Name: Supplementary Data 2

# Description: Differential expression analysis of lncRNA genes. WY, WY-14643; Resp, Response; WT, *Ppara*^+/+^; KO, *Ppara*^-/-^

File Name: Supplementary Data 3

# Description: LncRNAs responsive to activators of PPARα, CAR, and PXR in mouse liver.

Groups #1, #2: lncRNAs that are consistently induced, or consistently repressed, by all 3 nuclear receptors, as indicated; Groups #3, #4: lncRNAs consistently induced or repressed by PPARα and by CAR, but not by PXR; Groups #5, #6: lncRNAs consistently induced or repressed by PPARα and by PXR, but not by CAR; Groups #7, #8: lncRNAs induced or repressed by PPARα but showing the opposite responses to activators of CAR, PXR, or both receptors. See Melia et al, 2019 for genomic coordinates and gene and isoform structures of lncRNAs. ‘Inter’, intergenic lncRNA; ‘as’, lncRNA that is anti-sense to a protein-coding gene; ‘intra’, lncRNA that is intragenic to, and transcribed from the same strand as a protein-coding gene but whose exons do not overlap those of the protein-coding gene.
