# Supplementary material for: Long non-coding RNA Gm15441 attenuates hepatic inflammasome activation in response to PPARA agonism and fasting
Source: Nat Commun. 2020 Nov 17;11:5847. doi: 10.1038/s41467-020-19554-7 (PMC7673042; doi:10.1038/s41467-020-19554-7)
Supplement: Supplementary file 3 — Supplemental Data 1 [file 41467_2020_19554_MOESM3_ESM.pdf]

Supplementary Data 1. Differential expression analysis of RefSeq genes.

WY, WY-14643; Resp, Response; WT, *Ppara*$^{+/+}$; KO, *Ppara*$^{-/-}$

	**WY Resp. WT liver**	**WY Resp. KO liver**		**WY Resp. WT liver**	**WY Resp. KO liver**		**WY Resp. WT liver**	**WY Resp. KO liver**
**Gene ID**	**Exp. No. 332**	**Exp. No. 334**	**Gene ID**	**Exp. No. 332**	**Exp. No. 334**	**Gene ID**	**Exp. No. 332**	**Exp. No. 334**
FAM47E	Down	Down	SULT1A1	Down	0	PSMA5	Up	0
XAF1	Down	Down	TMEM19	Down	0	SLC27A1	Up	0
GM3776	Down	Down	ANTXR2	Down	0	SLC27A2	Up	0
SYTL1	Down	Down	ASL	Down	0	VNN3	Up	0
SUCNR1	Down	Down	GPR110	Down	0	SLC27A4	Up	0
CLIC5	Down	`	FGG	Down	0	ABCD2	Up	0
HAMP2	Down	Down	IFI44	Down	0	COPS6	Up	0
MEG3	Down	Down	ZBTB48	Down	0	ACOT1	Up	0
RAD51B	Up	Down	C8B	Down	0	EXO1	Up	0
HSPA1A	Up	Down	CES2A	Down	0	CFD	Up	0
SERPINA7	Up	Down	TRF	Down	0	ADRA1A	Up	0
XLR4A	0	Down	RDH5	Down	0	ADRB3	Up	0
PIK3C2B	0	Down	AKR1C14	Down	0	HPRT	Up	0
4833420G17RIK	0	Down	PLA1A	Down	0	HSPA1L	Up	0
MUP19	0	Down	IL1RAP	Down	0	HSPH1	Up	0
MUP12	0	Down	CYP2C50	Down	0	HSPB1	Up	0
GSTA1	0	Down	FUT10	Down	0	NR4A2	Up	0
MME	0	Down	RGS3	Down	0	SEMA5B	Up	0
CYP46A1	0	Down	BMF	Down	0	VLDLR	Up	0
IHH	0	Down	PRAF2	Down	0	SGK2	Up	0
REC114	0	Down	SARDH	Down	0	CHAF1A	Up	0
MTUS2	0	Down	TNIP2	Down	0	PDK4	Up	0
SLC8A3	0	Down	UGP2	Down	0	PMM1	Up	0
SUGCT	0	Down	VASN	Down	0	PDE7B	Up	0
FMR1NB	0	Down	SUSD4	Down	0	MFI2	Up	0
ACPP	0	Down	CBX7	Down	0	PTPLA	Up	0
MALAT1	0	Down	PPP1R1B	Down	0	ACOX1	Up	0
A630020A06	0	Down	SLC7A4	Down	0	HUNK	Up	0
3010033K07RIK	0	Down	CBS	Down	0	NAP1L1	Up	0

MYOM3	Down	Up	SLC22A7	Down	0	AQP3	Up	0
SAA1	Down	Up	BC021614	Down	0	MAP3K6	Up	0
GBP2B	Down	Up	HHAT	Down	0	UCHL3	Up	0
CDKN1A	Up	Up	6330416G13RIK	Down	0	CTPS	Up	0
LCN2	Up	Up	GCKR	Down	0	RUNDC3A	Up	0
MT2	Up	Up	C1S1	Down	0	HSD17B10	Up	0
DUSP8	Up	Up	FRS3	Down	0	ECH1	Up	0
MYC	Up	Up	CD207	Down	0	ATXN10	Up	0
MT1	Up	Up	TIFA	Down	0	GALK1	Up	0
PLIN5	Up	Up	FRMD4B	Down	0	PLA2G6	Up	0
LAD1	Up	Up	LRRC3	Down	0	NUDT5	Up	0
INSIG2	Up	Up	OASL1	Down	0	GAL3ST1	Up	0
ZFP455	0	Up	AKR1D1	Down	0	FANCA	Up	0
MUP13	0	Up	BC089597	Down	0	PKD2L2	Up	0
SCARA5	0	Up	GPX6	Down	0	TLR5	Up	0
GM1966	0	Up	CYP2C70	Down	0	ACADVL	Up	0
ARNTL	0	Up	CCM2L	Down	0	TUBA8	Up	0
DIO1	0	Up	GBP7	Down	0	FABP1	Up	0
SERPINA3C	0	Up	DKK4	Down	0	SPAG5	Up	0
GEM	0	Up	PPP1R42	Down	0	CTPS2	Up	0
MARCO	0	Up	VEPH1	Down	0	TRPM1	Up	0
RNASEL	0	Up	PITPNC1	Down	0	SLC4A4	Up	0
DLGAP1	0	Up	GLYAT	Down	0	ST3GAL6	Up	0
LYVE1	0	Up	CTH	Down	0	ABHD2	Up	0
CES4A	0	Up	TIFAB	Down	0	SLC23A2	Up	0
CD300E	0	Up	ARID5A	Down	0	PNLIPRP1	Up	0
TTLL8	0	Up	ANK3	Down	0	PDZRN3	Up	0
FAM107A	0	Up	ARHGAP9	Down	0	TYRO3	Up	0
MIR8108	0	Up	MGAT2	Down	0	NCAPG	Up	0
9030619P08RIK	0	Up	ZDHHC14	Down	0	ACSL4	Up	0
CYP2C44	Down	0	FAM129B	Down	0	HEBP2	Up	0
ADAMTS7	Down	0	ITPKA	Down	0	MAD2L1	Up	0
ZC4H2	Down	0	C8A	Down	0	PDSS1	Up	0
SLC13A5	Down	0	LRRK1	Down	0	HAO2	Up	0
MAML3	Down	0	UGT2B1	Down	0	PTBP2	Up	0
PIK3R6	Down	0	OSBP2	Down	0	PLCE1	Up	0

AMICA1	Down	0	IGJ	Down	0	PKP1	Up	0
I830012O16RIK	Down	0	SLC37A1	Down	0	HSD17B12	Up	0
ARMCX6	Down	0	CADM4	Down	0	DYNLL1	Up	0
ZFP658	Down	0	RDH9	Down	0	FADS2	Up	0
MUP21	Down	0	ABCA8A	Down	0	ARL2	Up	0
MUP20	Down	0	ACAT3	Down	0	PDCD5	Up	0
ECM2	Down	0	HSD3B2	Down	0	MTCH2	Up	0
ANKRD6	Down	0	NEURL3	Down	0	RAB9	Up	0
SLC22A28	Down	0	OLFM3	Down	0	ABCB9	Up	0
SHF	Down	0	NRN1	Down	0	COPZ2	Up	0
FAM189B	Down	0	BC029214	Down	0	PTPN4	Up	0
G2E3	Down	0	OBP2A	Down	0	UBE2L6	Up	0
CYP2C67	Down	0	SYNE4	Down	0	HACL1	Up	0
C330021F23RIK	Down	0	DPYD	Down	0	MTOR	Up	0
NTRK2	Down	0	DYNC2LI1	Down	0	FGF21	Up	0
CFHR2	Down	0	LIMD2	Down	0	RAET1D	Up	0
UGT2B36	Down	0	ITIH5	Down	0	ANKRD2	Up	0
GM4788	Down	0	TMEM71	Down	0	TRPM5	Up	0
ACMSD	Down	0	TXNDC16	Down	0	SLC25A20	Up	0
BBS1	Down	0	ADAMTS10	Down	0	PLIN4	Up	0
LIN7A	Down	0	DDX58	Down	0	XRCC2	Up	0
AGAP2	Down	0	PLXNB1	Down	0	ABHD1	Up	0
GLS2	Down	0	GBP9	Down	0	SEC61A2	Up	0
TMEM231	Down	0	ZFP454	Down	0	PTPLAD1	Up	0
GUCY1A2	Down	0	PODN	Down	0	CALN1	Up	0
DNAAF3	Down	0	OLFML1	Down	0	ZBTB32	Up	0
ACSL6	Down	0	AI118078	Down	0	USH2A	Up	0
9130409I23RIK	Down	0	DCLK3	Down	0	CDC42EP5	Up	0
CCDC160	Down	0	SNTG2	Down	0	HNRNPH1	Up	0
TNFSFM13	Down	0	OLFM2	Down	0	PXMP4	Up	0
FAM134B	Down	0	APOL9A	Down	0	ARPP19	Up	0
GM4841	Down	0	PYHIN1	Down	0	ACKR2	Up	0
PNPLA1	Down	0	1110051M20RIK	Down	0	HR	Up	0
RIPPLY1	Down	0	DNAIC1	Down	0	FIGNL1	Up	0
GM11437	Down	0	ARHGAP26	Down	0	GPRC5B	Up	0
KCNJ10	Down	0	MMD2	Down	0	SCUBE1	Up	0
PARP14	Down	0	2810007J24RIK	Down	0	LPAR3	Up	0

MUP3	Down	0	MYH10	Down	0	PEX19	Up	0
CYP2C68	Down	0	CATSPERD	Down	0	PKMYT1	Up	0
GBP10	Down	0	SP110	Down	0	ASPA	Up	0
GBP11	Down	0	ATP6V0D2	Down	0	TOR3A	Up	0
URAD	Down	0	GSAP	Down	0	PALMD	Up	0
ARRDC3	Down	0	FAM26F	Down	0	NDC80	Up	0
HSD11B1	Down	0	OSBPL8	Down	0	CML5	Up	0
SCIMP	Down	0	MRGPRE	Down	0	HILPDA	Up	0
SLC1A2	Down	0	SNHG11	Down	0	ISYNA1	Up	0
BRDT	Down	0	ARHGAP18	Down	0	CROT	Up	0
CPS1	Down	0	LRTM1	Down	0	EHHADH	Up	0
ATP8B4	Down	0	SLC22A30	Down	0	PANK1	Up	0
SETDB2	Down	0	SLC17A4	Down	0	ALG14	Up	0
KCNT2	Down	0	CAMK1D	Down	0	ASF1B	Up	0
CCNO	Down	0	KALRN	Down	0	RTN4	Up	0
GPAT2	Down	0	SLC41A2	Down	0	GINS4	Up	0
DHTKD1	Down	0	EIF5A2	Down	0	HSDL2	Up	0
HTRA4	Down	0	MKX	Down	0	TNFRSF23	Up	0
CCDC88B	Down	0	D630003M21RIK	Down	0	FABP4	Up	0
CES1B	Down	0	VGLL4	Down	0	MTAP	Up	0
METTL7A3	Down	0	E2F2	Down	0	EVPL	Up	0
SRPX2	Down	0	FAM198A	Down	0	MRPL17	Up	0
FETUB	Down	0	COLGALT2	Down	0	ABHD6	Up	0
NDUFA4L2	Down	0	PREX1	Down	0	NDUFA3	Up	0
F830016B08RIK	Down	0	KLHL23	Down	0	CHCHD6	Up	0
CYP2C69	Down	0	TMEM67	Down	0	DNAJC15	Up	0
CYP2D11	Down	0	ZFP334	Down	0	AIG1	Up	0
CYP3A59	Down	0	VTCN1	Down	0	CENPP	Up	0
GAS7	Down	0	NECAB1	Down	0	APTX	Up	0
NOS1AP	Down	0	FAM65B	Down	0	SPC25	Up	0
FGA	Down	0	CREB3L2	Down	0	PNKD	Up	0
CMAH	Down	0	DPY19L3	Down	0	NDUFB3	Up	0
NCAM2	Down	0	ZFP385B	Down	0	ZWINT	Up	0
CEACAM2	Down	0	LRRC4C	Down	0	GEMIN2	Up	0
SHANK2	Down	0	GULO	Down	0	MAP1LC3A	Up	0
LAIR1	Down	0	EGFLAM	Down	0	ASB17	Up	0
FCGBP	Down	0	SCAI	Down	0	RNF186	Up	0
WISP3	Down	0	TMEM241	Down	0	ETFDH	Up	0

NUCB2	Down	0	AI182371	Down	0	LONP2	Up	0
GM12250	Down	0	PLD4	Down	0	BAIAP2L1	Up	0
HSBP1L1	Down	0	TNFRSF14	Down	0	DSN1	Up	0
BC024139	Down	0	CAR5B	Down	0	SH3D21	Up	0
PPFIA4	Down	0	NOSTRIN	Down	0	CAND2	Up	0
TGTP2	Down	0	PIK3R3	Down	0	MASTL	Up	0
CES3B	Down	0	DSG1C	Down	0	ACBD4	Up	0
MYO1B	Down	0	GSTP2	Down	0	NKAIN1	Up	0
TTBK1	Down	0	NFASC	Down	0	ARL8B	Up	0
ITPRIPL1	Down	0	FAM19A2	Down	0	CDT1	Up	0
PLXDC1	Down	0	SPRN	Down	0	ZC3H13	Up	0
MSL3L2	Down	0	PLK5	Down	0	RETSAT	Up	0
LY6E	Down	0	FAT4	Down	0	DECR1	Up	0
PHF11C	Down	0	WFDC21	Down	0	MMD	Up	0
IGLON5	Down	0	FAM229B	Down	0	AGPAT2	Up	0
3110052M02RIK	Down	0	FAM25C	Down	0	SLC25A30	Up	0
KLHL33	Down	0	GBP6	Down	0	LSM3	Up	0
APOL9B	Down	0	LZTS3	Down	0	PLLP	Up	0
BDH2	Down	0	RANBP3L	Down	0	PPP2R2D	Up	0
LRRC56	Down	0	ELMO1	Down	0	CELA3B	Up	0
LOC100038947	Down	0	CES2B	Down	0	SUMF2	Up	0
CCDC162	Down	0	4430402I18RIK	Down	0	TUBB6	Up	0
GPR155	Down	0	B4GALNT3	Down	0	PPCS	Up	0
GM7694	Down	0	HCRTR2	Down	0	ZWILCH	Up	0
LOC100048884	Down	0	SERPINA11	Down	0	3010026O09RIK	Up	0
MUP16	Down	0	CYP2D12	Down	0	DCAKD	Up	0
MUP17	Down	0	KNG2	Down	0	CDCA8	Up	0
H2-Q9	Down	0	CYP2C54	Down	0	NHP2	Up	0
AI607873	Down	0	H2-AB1	Down	0	FAM195A	Up	0
SMIM22	Down	0	TES	Down	0	GGCT	Up	0
BIN2	Down	0	ACE	Down	0	ETFB	Up	0
C920025E04RIK	Down	0	H2-Q6	Down	0	MOGAT1	Up	0
AFF3	Down	0	EGFR	Down	0	CGREF1	Up	0
DDX60	Down	0	H19	Down	0	FITM1	Up	0
PZP	Down	0	GM5424	Down	0	COMTD1	Up	0
AATK	Down	0	GM4956	Down	0	GINS1	Up	0
ADRA1B	Down	0	SERPINA4-PS1	Down	0	1700030J22RIK	Up	0

ADRB1	Down	0	CYP2D13	Down	0	TEN1	Up	0
AMD2	Down	0	GM10319	Down	0	HFE2	Up	0
ANG	Down	0	BC029722	Down	0	TRIP13	Up	0
BIRC3	Down	0	MIR99AHG	Down	0	ZSWIM7	Up	0
ASS1	Down	0	2610035D17RIK	Down	0	ANXA13	Up	0
CIITA	Down	0	BC024386	Down	0	TMEM147	Up	0
CAR8	Down	0	RIAN	Down	0	KRTCAP3	Up	0
CAR3	Down	0	MIRG	Down	0	2010003K11RIK	Up	0
SERPINA6	Down	0	1700018L02RIK	Down	0	APITD1	Up	0
CD37	Down	0	MARCKSL1-PS4	Down	0	NT5DC2	Up	0
CD48	Down	0	SNHG12	Down	0	GOLM1	Up	0
CFI	Down	0	4930581F22RIK	Down	0	LYSMD2	Up	0
CHRM1	Down	0	MIR24-1	Down	0	GPATCH2L	Up	0
DMBT1	Down	0	MIR22	Down	0	ATAD2	Up	0
CYBA	Down	0	MIR568	Down	0	QPCT	Up	0
CYP26A1	Down	0	MIR22HG	Down	0	SLC25A33	Up	0
CYP2A5	Down	0	1500017E21RIK	Down	0	FAM213A	Up	0
CYP2C29	Down	0	ADH6-PS1	Down	0	GFOD2	Up	0
CYP2F2	Down	0	CYP2C53-PS	Down	0	KDSR	Up	0
CYP3A11	Down	0	5430416N02RIK	Down	0	4921507P07RIK	Up	0
CYP7B1	Down	0	A730020M07RIK	Down	0	MICALCL	Up	0
DACH1	Down	0	GM16548	Down	0	GPR39	Up	0
GADD45A	Down	0	C730036E19RIK	Down	0	RFX4	Up	0
EEF2K	Down	0	0610005C13RIK	Down	0	PEX1	Up	0
CELA2A	Down	0	2310001H17RIK	Down	0	FAM110C	Up	0
ENPEP	Down	0	KANTR	Down	0	METTL7B	Up	0
CES1C	Down	0	2310040G24RIK	Down	0	SLC41A3	Up	0
ESR1	Down	0	GM16793	Down	0	ARHGEF3	Up	0
FGFR3	Down	0	GM19522	Down	0	VWA8	Up	0
FGFR4	Down	0	3110045C21RIK	Down	0	SYCE2	Up	0

FMO3	Down	0	GM10804	Down	0	CENPU	Up	0
FST	Down	0	GM12718	Down	0	ACSL5	Up	0
FZD8	Down	0	4930480G23RIK	Down	0	APMAP	Up	0
GNA14	Down	0	GDAP10	Down	0	EPN3	Up	0
GNAT1	Down	0	1700012D01RIK	Down	0	PAQR7	Up	0
GNAT2	Down	0	GM17644	Down	0	ENDOD1	Up	0
GPLD1	Down	0	2900009J06RIK	Down	0	WDR73	Up	0
GRM8	Down	0	2900076A07RIK	Down	0	ZDHHC13	Up	0
CXCL1	Down	0	5730435O14RIK	Down	0	DDX28	Up	0
GSTA2	Down	0	4933438K21RIK	Down	0	1600014K23RIK	Up	0
GSTM2	Down	0	1700028E10RIK	Down	0	SLC39A5	Up	0
GSTM6	Down	0	0610031O16RIK	Down	0	SLC35F2	Up	0
GUCA1A	Down	0	4930470H14RIK	Down	0	SULF2	Up	0
H2-BL	Down	0	1700092C10RIK	Down	0	CHAF1B	Up	0
H2-T24	Down	0	GM16063	Down	0	DDHD2	Up	0
MR1	Down	0	AW112010	Down	0	PYGO1	Up	0
FOXQ1	Down	0	GM20319	Down	0	2610020H08RIK	Up	0
HSD3B5	Down	0	MIR7219	Down	0	CENPN	Up	0
SDC2	Down	0	4930459C07RIK	Down	0	CDA	Up	0
HTR1D	Down	0	ZFP941	Up	0	ORAOV1	Up	0
IFI47	Down	0	PLEKHA8	Up	0	WDR89	Up	0
IFIT1	Down	0	UTP14B	Up	0	PRCP	Up	0
IL12RB1	Down	0	NAT8L	Up	0	ANGPTL1	Up	0
IL15	Down	0	RAB44	Up	0	TMEM135	Up	0
IL18	Down	0	TACC2	Up	0	SASS6	Up	0
IL1B	Down	0	SLC25A42	Up	0	BMPER	Up	0
INHBA	Down	0	RNASE13	Up	0	1700056E22RIK	Up	0
LAG3	Down	0	HSPB6	Up	0	FCF1	Up	0
BLNK	Down	0	HS3ST6	Up	0	PHF19	Up	0
MASP1	Down	0	WFDC16	Up	0	RIN2	Up	0
MUG1	Down	0	E2F8	Up	0	GRIP1	Up	0
MUG2	Down	0	NRP	Up	0	CEP55	Up	0

NAB2	Down	0	LMAN2L	Up	0	ACOT12	Up	0
NFKBIE	Down	0	RSAD1	Up	0	CCDC3	Up	0
MYCN	Down	0	SCN4B	Up	0	XRCC3	Up	0
NPTX1	Down	0	TRIM6	Up	0	TMED5	Up	0
ORM1	Down	0	GM4952	Up	0	ELOVL7	Up	0
OTC	Down	0	SLC25A34	Up	0	LRRC48	Up	0
PAH	Down	0	4930404N11RIK	Up	0	SPATA17	Up	0
PDE9A	Down	0	ZMYND12	Up	0	KBTBD11	Up	0
TULP2	Down	0	ANKS6	Up	0	CCDC146	Up	0
SERPINF2	Down	0	ADAMTS15	Up	0	FAM183B	Up	0
PLSCR2	Down	0	PLA2G4F	Up	0	ACYP2	Up	0
PRKG2	Down	0	ZBTB45	Up	0	MORC4	Up	0
PIPOX	Down	0	ZFP667	Up	0	4930402H24RIK	Up	0
PTGDS	Down	0	SERINC4	Up	0	NLN	Up	0
RAC2	Down	0	SNX22	Up	0	4930579G24RIK	Up	0
RASA3	Down	0	CDC6	Up	0	RAB30	Up	0
RGS4	Down	0	ENTPD5	Up	0	PREX2	Up	0
SCN7A	Down	0	PKNOX2	Up	0	TMEM98	Up	0
CX3CL1	Down	0	SPECC1	Up	0	GSTK1	Up	0
ST3GAL1	Down	0	SYBU	Up	0	DET1	Up	0
SLC17A1	Down	0	KLHDC9	Up	0	PPP1R3G	Up	0
SLC3A1	Down	0	LRRC14B	Up	0	SLC52A2	Up	0
SERPINA1A	Down	0	NTRK1	Up	0	STYXL1	Up	0
SERPINA1B	Down	0	1700039E15RIK	Up	0	SPATC1L	Up	0
SERPINA1C	Down	0	YEATS2	Up	0	SRXN1	Up	0
SERPINA1E	Down	0	FANCD2	Up	0	DTL	Up	0
SERPINA3N	Down	0	8-Mar	Up	0	UNC5B	Up	0
SERPINA3M	Down	0	5031439G07RIK	Up	0	TTLL11	Up	0
SERPINB9	Down	0	UAP1L1	Up	0	CALR3	Up	0
SLC6A6	Down	0	FHDC1	Up	0	ST5	Up	0
TBXA2R	Down	0	ABCC4	Up	0	2700049A03RIK	Up	0
PHLDA1	Down	0	NLRP12	Up	0	TICRR	Up	0

TERT	Down	0	BC048644	Up	0	MRAP	Up	0
THRSP	Down	0	LRRC8B	Up	0	KCNK10	Up	0
TNNC1	Down	0	ZCCHC16	Up	0	POLQ	Up	0
TNFSF10	Down	0	ZFP872	Up	0	SLA2	Up	0
UOX	Down	0	THNSL2	Up	0	LBH	Up	0
WAS	Down	0	RUFY4	Up	0	PLB1	Up	0
ZAP70	Down	0	TMEM136	Up	0	HTRA3	Up	0
CHRNB1	Down	0	PNLDC1	Up	0	ZFP449	Up	0
ADCY9	Down	0	ST3GAL5	Up	0	NOL3	Up	0
AVIL	Down	0	CACNB4	Up	0	EFCAB11	Up	0
ALAS2	Down	0	TOMM40L	Up	0	ARMC9	Up	0
ALDOC	Down	0	COL28A1	Up	0	STPG1	Up	0
AMD1	Down	0	WDR93	Up	0	WRNIP1	Up	0
AKR1B7	Down	0	GM11992	Up	0	DLST	Up	0
C3AR1	Down	0	DHRS4	Up	0	ZFP202	Up	0
C4B	Down	0	LIPE	Up	0	CNNM1	Up	0
CAR1	Down	0	RAD54B	Up	0	NRG4	Up	0
CAR11	Down	0	IL1RN	Up	0	DMPK	Up	0
CASP3	Down	0	KNTC1	Up	0	ZFP423	Up	0
CCRN4L	Down	0	OIP5	Up	0	KRT23	Up	0
CD83	Down	0	PROCA1	Up	0	RAB34	Up	0
CDH1	Down	0	MUP2	Up	0	NPNT	Up	0
CFH	Down	0	CERKL	Up	0	CNNM4	Up	0
CHRD	Down	0	BSG	Up	0	AMN	Up	0
CISH	Down	0	NRP2	Up	0	OPHN1	Up	0
CLCN2	Down	0	PTPN6	Up	0	FAM126A	Up	0
CCR3	Down	0	SLC16A5	Up	0	TAC4	Up	0
CCR5	Down	0	THG1L	Up	0	IMPA2	Up	0
CNP	Down	0	DDIAS	Up	0	HSD17B11	Up	0
CYP1A1	Down	0	UNC79	Up	0	RAD51C	Up	0
CYP1A2	Down	0	EFCAB3	Up	0	RIMS2	Up	0
CYP2C37	Down	0	ADAMTS14	Up	0	HIST2H3C2	Up	0
CYP2C40	Down	0	OXTR	Up	0	DEF8	Up	0
DBT	Down	0	RAP1GAP	Up	0	PAWR	Up	0
LEFTY1	Down	0	NIPAL1	Up	0	GRHPR	Up	0
SAMD9L	Down	0	ZFP174	Up	0	RNF144A	Up	0
F7	Down	0	TLN2	Up	0	ACO2	Up	0
FCGR1	Down	0	UGGT2	Up	0	PASK	Up	0
FES	Down	0	MFSD7B	Up	0	ACAA1A	Up	0
FOS	Down	0	MUP6	Up	0	KIRREL	Up	0

GAMT	Down	0	DGKH	Up	0	PAPLN	Up	0
GHR	Down	0	MYH7B	Up	0	ROGDI	Up	0
GM2A	Down	0	CISD3	Up	0	P2RY14	Up	0
GNMT	Down	0	FAM131C	Up	0	ACOT8	Up	0
GOT1	Down	0	CYP4A32	Up	0	SLC5A2	Up	0
GSTA4	Down	0	WFIKKN1	Up	0	MEF2D	Up	0
GSTM1	Down	0	CYP4F17	Up	0	CHRDL2	Up	0
GSTM3	Down	0	CYP2A22	Up	0	ARHGAP27	Up	0
H2-Q7	Down	0	COL6A6	Up	0	4931406C07RIK	Up	0
H2-T10	Down	0	CENPW	Up	0	NCAPG2	Up	0
H2-T23	Down	0	CBFA2T3	Up	0	STK17B	Up	0
HAL	Down	0	CELF2	Up	0	EHD4	Up	0
HC	Down	0	UHRF1	Up	0	MMADHC	Up	0
HEXB	Down	0	BC048609	Up	0	SPON2	Up	0
IER2	Down	0	PLIN1	Up	0	ACACB	Up	0
IFIT3	Down	0	8-Sep	Up	0	RHOU	Up	0
IGF1	Down	0	4930506M07RIK	Up	0	CDCP1	Up	0
IL18BP	Down	0	CORIN	Up	0	AKR1C18	Up	0
IRX1	Down	0	RAD54L	Up	0	NCALD	Up	0
KIF9	Down	0	FAM178B	Up	0	LARS	Up	0
KLF12	Down	0	MUP8	Up	0	SYT12	Up	0
MAFB	Down	0	GM9992	Up	0	ACOT2	Up	0
LAMA3	Down	0	ACSS3	Up	0	ACOT3	Up	0
LECT1	Down	0	ADTRP	Up	0	ACOT4	Up	0
LECT2	Down	0	ALS2CL	Up	0	ELOVL5	Up	0
PSMB8	Down	0	SSC4D	Up	0	SLC22A27	Up	0
LST1	Down	0	TMEM184A	Up	0	TYMP	Up	0
MXD1	Down	0	KIF26B	Up	0	PPP2R4	Up	0
MBL2	Down	0	MDM1	Up	0	PLA2G16	Up	0
MPEG1	Down	0	PKP3	Up	0	GALNT2	Up	0
MX1	Down	0	RMI2	Up	0	DLGAP5	Up	0
NEB	Down	0	LRRC51	Up	0	ACAT1	Up	0
NNMT	Down	0	MINOS1	Up	0	CHRNA2	Up	0
ORM2	Down	0	EME2	Up	0	CHPT1	Up	0
PIGR	Down	0	PCSK5	Up	0	KLHDC8A	Up	0
PPIB	Down	0	MTTP	Up	0	SLC24A1	Up	0
PRLR	Down	0	MAP3K12	Up	0	NCAPH	Up	0

PRODH	Down	0	OSBPL3	Up	0	ELMOD3	Up	0
RGS16	Down	0	TRIM13	Up	0	CSAD	Up	0
SAA2	Down	0	CHADL	Up	0	HIP1R	Up	0
SAA3	Down	0	DNAH6	Up	0	PEX16	Up	0
SAA4	Down	0	RAD18	Up	0	LPCAT3	Up	0
APCS	Down	0	PPP1R3E	Up	0	PRC1	Up	0
SCNN1A	Down	0	EDA	Up	0	AY074887	Up	0
CCL9	Down	0	RPGR	Up	0	ACNAT2	Up	0
SELP	Down	0	KCNMB3	Up	0	BAG2	Up	0
SEMA3E	Down	0	GM3336	Up	0	SLC44A3	Up	0
SLC25A14	Down	0	KIFC1	Up	0	DUOXA1	Up	0
SNAI2	Down	0	DCDC2B	Up	0	CHTF18	Up	0
SORL1	Down	0	A630076J17RIK	Up	0	DHRS7B	Up	0
SERPINA3K	Down	0	FER1L5	Up	0	ACOT5	Up	0
SERPINB8	Down	0	GM867	Up	0	MFSD7C	Up	0
TFF3	Down	0	KBTBD12	Up	0	IFI27L2B	Up	0
TGTP1	Down	0	COL12A1	Up	0	HAUS4	Up	0
TNFSF12	Down	0	ITGA10	Up	0	SMOX	Up	0
XIRP1	Down	0	ACADL	Up	0	SDCBP2	Up	0
GREM2	Down	0	ACADM	Up	0	HADHB	Up	0
MMP17	Down	0	PLIN2	Up	0	NXNL1	Up	0
SPRY4	Down	0	ADRA2A	Up	0	TOX	Up	0
WIF1	Down	0	ALPL	Up	0	GRHL1	Up	0
MAP2K6	Down	0	ALDH3A2	Up	0	CENPI	Up	0
HOMER2	Down	0	ALDOA	Up	0	FANCI	Up	0
OMD	Down	0	AQP7	Up	0	RHBDD2	Up	0
AOAH	Down	0	RHOC	Up	0	KRT79	Up	0
IRF5	Down	0	ATF3	Up	0	ACAA1B	Up	0
AHSG	Down	0	PHB2	Up	0	ERCC6L	Up	0
C2	Down	0	HRK	Up	0	HPDL	Up	0
C9	Down	0	BLM	Up	0	OLFR20	Up	0
CD4	Down	0	ANXA2	Up	0	SLCO4A1	Up	0
HAPLN1	Down	0	CALCR	Up	0	STX3	Up	0
FLT3L	Down	0	CCNG2	Up	0	SLC18A1	Up	0
GSTP1	Down	0	CD36	Up	0	SLC16A11	Up	0

LIFR	Down	0	SCARB2	Up	0	PHOSPHO1	Up	0
PSMB9	Down	0	CD9	Up	0	OPLAH	Up	0
MX2	Down	0	CDK1	Up	0	CPNE5	Up	0
ORM3	Down	0	CHEK1	Up	0	SLC36A2	Up	0
PKLR	Down	0	CHKB	Up	0	ADAM32	Up	0
PSMB10	Down	0	CHIL1	Up	0	GRWD1	Up	0
TTR	Down	0	CIDEA	Up	0	CLSTN3	Up	0
FGD2	Down	0	ELOVL3	Up	0	BC030867	Up	0
NAGLU	Down	0	COL11A1	Up	0	FAM102A	Up	0
SLCO1A1	Down	0	CPOX	Up	0	CMTM4	Up	0
CYP2G1	Down	0	CRAT	Up	0	ACSF2	Up	0
LY75	Down	0	CSF2RB	Up	0	IGSF11	Up	0
GCAT	Down	0	CTSE	Up	0	SPIRE2	Up	0
TNFRSF19	Down	0	CYCS	Up	0	MAB21L3	Up	0
HEYL	Down	0	CYP17A1	Up	0	CYP4A12B	Up	0
ZFP354C	Down	0	CYP4A14	Up	0	SLC16A13	Up	0
ENPP2	Down	0	DBI	Up	0	TMPRSS7	Up	0
NOX4	Down	0	DEFB1	Up	0	ZC3HAV1L	Up	0
CFHR1	Down	0	ECT2	Up	0	TBC1D25	Up	0
ISG15	Down	0	ENC1	Up	0	MEGF11	Up	0
FBXO17	Down	0	EPHX2	Up	0	TMEM120A	Up	0
NT5C	Down	0	EPS8	Up	0	ACOT6	Up	0
SYT3	Down	0	FABP2	Up	0	C330027C09RIK	Up	0
C6	Down	0	ACSL1	Up	0	SERINC2	Up	0
BOK	Down	0	FAS	Up	0	OTOP1	Up	0
BNIP2	Down	0	FBP2	Up	0	SEL1L3	Up	0
IRF7	Down	0	SMC2	Up	0	AGPAT9	Up	0
PPP1R3C	Down	0	FRAT1	Up	0	ADPRHL1	Up	0
VAMP5	Down	0	FUT1	Up	0	CCDC14	Up	0
RAB25	Down	0	FZD6	Up	0	SERPINB1C	Up	0
TCIRG1	Down	0	G0S2	Up	0	PRUNE	Up	0
HPX	Down	0	GABRB2	Up	0	RBMX2	Up	0

GBP3	Down	0	SLC6A9	Up	0	FITM2	Up	0
ITIH4	Down	0	GRN	Up	0	DCUN1D3	Up	0
ABCC6	Down	0	GYK	Up	0	RCCD1	Up	0
LGALS8	Down	0	HADH	Up	0	TDRP	Up	0
SELENBP2	Down	0	HCN2	Up	0	UBTD2	Up	0
FMN2	Down	0	HELLS	Up	0	IBA57	Up	0
HGFAC	Down	0	HMGB3	Up	0	FAM73A	Up	0
HES6	Down	0	HMGCL	Up	0	IMPG2	Up	0
GRASP	Down	0	HMGCS2	Up	0	CDH22	Up	0
CXCL14	Down	0	HSD17B4	Up	0	PTRH2	Up	0
EXTL1	Down	0	HSPA2	Up	0	FANCB	Up	0
WSB1	Down	0	HSPE1	Up	0	MDFIC	Up	0
SLC22A4	Down	0	HYAL1	Up	0	TRPM4	Up	0
TLE2	Down	0	IDUA	Up	0	TMEM65	Up	0
NUPR1	Down	0	IMPACT	Up	0	NAV2	Up	0
SLC2A5	Down	0	INHBE	Up	0	ACAD11	Up	0
IKBKE	Down	0	INPP1	Up	0	D330045A20RIK	Up	0
DUSP14	Down	0	ITGA2	Up	0	CHCHD10	Up	0
CYP2D22	Down	0	ITGA6	Up	0	ALS2CR12	Up	0
PDE4B	Down	0	JRK	Up	0	FAM73B	Up	0
SULT1B1	Down	0	LAMB3	Up	0	TMEM139	Up	0
B3GALNT1	Down	0	ANPEP	Up	0	LRRC39	Up	0
B3GALT1	Down	0	LPL	Up	0	SPTLC3	Up	0
SLCO1B2	Down	0	MCM3	Up	0	LRFN3	Up	0
SULT5A1	Down	0	MCM2	Up	0	SLC35G1	Up	0
SRSF4	Down	0	MCM4	Up	0	TMEM245	Up	0
ABCB11	Down	0	MCM5	Up	0	CLSPN	Up	0
FABP7	Down	0	MCM6	Up	0	OSCAR	Up	0
CXCL10	Down	0	MCM7	Up	0	RAPGEF5	Up	0
EVC	Down	0	SMCP	Up	0	P3H4	Up	0
PIWIL2	Down	0	MEIG1	Up	0	6430573F11RIK	Up	0
SH2D2A	Down	0	ME1	Up	0	FSD1L	Up	0
TTYH1	Down	0	MDH2	Up	0	TOPBP1	Up	0
CRYBB3	Down	0	MPP1	Up	0	TMCC3	Up	0
RSAD2	Down	0	MTNR1A	Up	0	A530016L24RIK	Up	0
HYOU1	Down	0	GADD45B	Up	0	EXOC6B	Up	0

PRG4	Down	0	MYO1C	Up	0	GNAL	Up	0
DEXI	Down	0	NFE2L1	Up	0	CENPT	Up	0
CES1G	Down	0	MAP4K4	Up	0	ATP8B5	Up	0
RNASE4	Down	0	NMT2	Up	0	STRIP2	Up	0
BCO1	Down	0	NXPH1	Up	0	DAB1	Up	0
SMPD3	Down	0	P2RY1	Up	0	TMEM1<br>54	Up	0
SQRDL	Down	0	PCTP	Up	0	LRRN4	Up	0
MOXD1	Down	0	PDE1B	Up	0	CYP4F3<br>9	Up	0
DACT1	Down	0	PEG3	Up	0	SYNPO	Up	0
SYT9	Down	0	PFKFB1	Up	0	SLC9A7	Up	0
FN3K	Down	0	ABCB4	Up	0	TMTC2	Up	0
FNDC4	Down	0	PITX3	Up	0	PSAT1	Up	0
CYP4F14	Down	0	SERPINE1	Up	0	ACAA2	Up	0
SLC15A3	Down	0	PRIM1	Up	0	ZFP3	Up	0
APOC3	Down	0	PSMB4	Up	0	SUN3	Up	0
GPM6B	Down	0	PSMC3IP	Up	0	CCDC73	Up	0
H2-Q8	Down	0	PSMD4	Up	0	TUSC5	Up	0
KNG1	Down	0	PTGFR	Up	0	EME1	Up	0
C1RA	Down	0	PVRL2	Up	0	ESYT3	Up	0
ETHE1	Down	0	ABCD3	Up	0	MED12L	Up	0
CML1	Down	0	RAD51AP1	Up	0	TGM4	Up	0
BMYC	Down	0	RAET1A	Up	0	AIFM2	Up	0
RTP4	Down	0	RAET1B	Up	0	FA2H	Up	0
TMEM86B	Down	0	RAET1C	Up	0	AMIGO2	Up	0
CTNNBIP1	Down	0	RDH16	Up	0	HIST2H<br>3C1	Up	0
AOX3	Down	0	RPIA	Up	0	CXCR1	Up	0
CYP2D40	Down	0	RRM1	Up	0	CIDEC	Up	0
UBA7	Down	0	RRM2	Up	0	ZSWIM3	Up	0
ICAM4	Down	0	S100A10	Up	0	GALE	Up	0
PDE6H	Down	0	SCD1	Up	0	ZDHHC<br>2	Up	0
HDAC9	Down	0	ST3GAL2	Up	0	NANOS<br>1	Up	0
CYP27A1	Down	0	ST6GALNAC<br>2	Up	0	NRG1	Up	0
CYP4F16	Down	0	ST8SIA3	Up	0	RNF24	Up	0
NUDT7	Down	0	STIL	Up	0	THEMIS	Up	0
HAAO	Down	0	SLC16A1	Up	0	SLC35F1	Up	0
TCTEX1D2	Down	0	SPRR1A	Up	0	COQ4	Up	0
IFITM3	Down	0	TEAD1	Up	0	PRRG4	Up	0
FBXO36	Down	0	TECTB	Up	0	NCEH1	Up	0
ARL4D	Down	0	TGM2	Up	0	DCTD	Up	0
THEM5	Down	0	TGM3	Up	0	FAR2	Up	0

TMEM218	Down	0	RAN	Up	0	ACAD12	Up	0
ACOT11	Down	0	TPM2	Up	0	GINS2	Up	0
RDM1	Down	0	TGOLN1	Up	0	ADCK2	Up	0
COL27A1	Down	0	TGOLN2	Up	0	HADHA	Up	0
ACADSB	Down	0	TUBB2A	Up	0	TMEM56	Up	0
PROZ	Down	0	UCP3	Up	0	ARHGAP11A	Up	0
HMGN3	Down	0	UPP1	Up	0	FFAR4	Up	0
LRP2BP	Down	0	WNT11	Up	0	GPR135	Up	0
DYX1C1	Down	0	WNT5A	Up	0	TMEM59L	Up	0
ALDH3B1	Down	0	ADIPOQ	Up	0	A430033K04RIK	Up	0
IAH1	Down	0	ADAM11	Up	0	KIF20B	Up	0
TCTN2	Down	0	ADH7	Up	0	DSCC1	Up	0
SERPINA12	Down	0	ANXA5	Up	0	TONSL	Up	0
PPP1R14A	Down	0	BRCA2	Up	0	PLD6	Up	0
ZFP579	Down	0	CAPN2	Up	0	COX6B2	Up	0
LRRC16A	Down	0	CAR2	Up	0	COX19	Up	0
PDGFRL	Down	0	CASP8	Up	0	SCYL2	Up	0
HAGHL	Down	0	CCNA2	Up	0	FERMT1	Up	0
CHAC1	Down	0	CCT3	Up	0	BHLHB9	Up	0
SULT1C2	Down	0	CD24A	Up	0	RAET1E	Up	0
GSDMD	Down	0	CDC45	Up	0	PAQR9	Up	0
C1QTNF2	Down	0	CPT1B	Up	0	IGSF21	Up	0
ERP27	Down	0	CPT2	Up	0	C2CD4C	Up	0
SAT2	Down	0	CYP4A10	Up	0	CASC4	Up	0
ENHO	Down	0	DAPK2	Up	0	IFNK	Up	0
RPAIN	Down	0	ECI1	Up	0	UNC93A	Up	0
1810046K07RIK	Down	0	DNASE1	Up	0	DOT1L	Up	0
MCM10	Down	0	RHBDF1	Up	0	MMS22L	Up	0
SDR9C7	Down	0	KHDRBS3	Up	0	CTIF	Up	0
METTL7A1	Down	0	FABP3	Up	0	NEIL2	Up	0
CD209G	Down	0	FCGRT	Up	0	CYP4A31	Up	0
AFAP1	Down	0	FPGS	Up	0	UGT1A1	Up	0
IYD	Down	0	GPD1	Up	0	CHRM2	Up	0
ALDH1L1	Down	0	GPD2	Up	0	CCDC120	Up	0
DCLK2	Down	0	GNGT1	Up	0	UGT3A1	Up	0
ARRDC2	Down	0	GSTT2	Up	0	RCAN2	Up	0
LDHD	Down	0	HSPD1	Up	0	MARVELD3	Up	0

SNTG1	Down	0	HSPA1B	Up	0	Sept5	Up	0
CYP2U1	Down	0	HSP90AA1	Up	0	XIST	Up	0
IZUMO4	Down	0	SLC6A4	Up	0	GM5088	Up	0
IFIH1	Down	0	ID1	Up	0	DIO3OS	Up	0
RARRES2	Down	0	ITGA4	Up	0	NEAT1	Up	0
SLC46A3	Down	0	KCNAB1	Up	0	ATP10D	Up	0
SELO	Down	0	KCNK3	Up	0	CHKBCPT1B	Up	0
ETNPPL	Down	0	FABP5	Up	0	CDK3-PS	Up	0
1300017J02RIK	Down	0	LAMC1	Up	0	B430010I23RIK	Up	0
SERPINA9	Down	0	LEPR	Up	0	A330069E16RIK	Up	0
F11	Down	0	LGALS4	Up	0	C230091D08RIK	Up	0
CYP2C55	Down	0	LGALS6	Up	0	A330040F15RIK	Up	0
UGT2A3	Down	0	LIG1	Up	0	1700008J07RIK	Up	0
ALDH1B1	Down	0	LMO4	Up	0	GM10638	Up	0
TTC39C	Down	0	LY6D	Up	0	1700020N01RIK	Up	0
3110082I17RIK	Down	0	MID1	Up	0	GM10012	Up	0
1700049G17RIK	Down	0	MTHFR	Up	0	2310068J16RIK	Up	0
STRA6L	Down	0	MYOM1	Up	0	PNPLA2	Up	0
PAQR8	Down	0	NCK2	Up	0	D430020J02RIK	Up	0
IQCE	Down	0	NEK2	Up	0	GM10069	Up	0
TMC5	Down	0	ORC1	Up	0	GM15760	Up	0
SNX29	Down	0	HSPA4L	Up	0	3930402G23RIK	Up	0
PPP4R4	Down	0	PDE4D	Up	0	B430212C06RIK	Up	0
PYROXD2	Down	0	PEX11A	Up	0	BC031361	Up	0
RSPH9	Down	0	ABCB1B	Up	0	E030024N20RIK	Up	0
TMEM14A	Down	0	PLTP	Up	0	GM10790	Up	0
APOL7A	Down	0	POLD1	Up	0	1700048O20RIK	Up	0
AP3M2	Down	0	POLE	Up	0	D330041H03RIK	Up	0

GBP8	Down	0	POLE2	Up	0	AI507597	Up	0
ZFP773	Down	0	POU4F1	Up	0	4632428C04RIK	Up	0
ABHD14B	Down	0	PRKDC	Up	0	MIR3091	Up	0
UPP2	Down	0	PRTN3	Up	0	1300002E11RIK	Up	0
FAM81A	Down	0	PSAP	Up	0	9330159M07RIK	Up	0
IFI27L2A	Down	0	PTGFRN	Up	0	GM15663	Up	0
2310014L17RIK	Down	0	RAD51	Up	0	1700023L04RIK	Up	0
CNRIP1	Down	0	RAD51D	Up	0	CTCFLOS	Up	0
CCDC151	Down	0	SCNN1G	Up	0	9430091E24RIK	Up	0
CRYL1	Down	0	SHCBP1	Up	0	B130034C11RIK	Up	0
AK7	Down	0	SLC16A7	Up	0	GM15441	Up	0
ABCG3	Down	0	SLC22A3	Up	0	GM15401	Up	0
AKR1C6	Down	0	SLC22A5	Up	0	GM19619	Up	0
SLCO1A4	Down	0	SOX9	Up	0	AV051173	Up	0
F13B	Down	0	SYCP3	Up	0	1700027J07RIK	Up	0
MUP1	Down	0	TIMELESS	Up	0	4931402G19RIK	Up	0
REN1	Down	0	TOP2A	Up	0	C130083M11RIK	Up	0
REN2	Down	0	TRAIP	Up	0	9430037G07RIK	Up	0
CDO1	Down	0	UCP2	Up	0	4930556M19RIK	Up	0
NGFR	Down	0	UCK1	Up	0	2310039L15RIK	Up	0
OAS1C	Down	0	UNC119	Up	0	2410003L11RIK	Up	0
MPV17L	Down	0	VNN1	Up	0	4930413G21RIK	Up	0
DQX1	Down	0	TRPV2	Up	0	C330022C24RIK	Up	0
CELA1	Down	0	BACE1	Up	0	FAM219AOS	Up	0
PDE6C	Down	0	XCR1	Up	0	GM10865	Up	0
NREP	Down	0	IMPDH1	Up	0	9530080O11RIK	Up	0

CLEC2H	Down	0	MAB21L2	Up	0	GM4890	Up	0
HRG	Down	0	MGLL	Up	0	GM5420	Up	0
S1PR5	Down	0	TENM3	Up	0	GM1976	Up	0
GPR98	Down	0	ECI2	Up	0	GM10677	Up	0
SLC13A3	Down	0	SGCB	Up	0	4930506C21RIK	Up	0
ACSM1	Down	0	SGCG	Up	0	STXBP3B	Up	0
STEAP4	Down	0	SRP54A	Up	0	MIR7676-1	Up	0
HNMT	Down	0	ABCG2	Up	0	MIR7676-2	Up	0
BAIAP2	Down	0	DECR2	Up	0	MIR8093	Up	0
DNAH5	Down	0	MAPK13	Up	0	RPL14-PS1	Up	0
						GM1600	Up	0
