# Supplementary material for: Long non-coding RNA Gm15441 attenuates hepatic inflammasome activation in response to PPARA agonism and fasting
Source: Nat Commun. 2020 Nov 17;11:5847. doi: 10.1038/s41467-020-19554-7 (PMC7673042; doi:10.1038/s41467-020-19554-7)
Supplement: Supplementary file 4 — Supplemental Data 2 [file 41467_2020_19554_MOESM4_ESM.pdf]

Supplementary Data 2. Differential expression analysis of lncRNA genes.

WY, WY-14643; Resp, Response; WT, *Ppara*$^{+/+}$; KO, *Ppara*$^{-/-}$

	**WY Response in WT liver (246 Up, 202 down)**	**WY Response in KO liver (16 Up, 5 down)**		**WY Response in WT liver (246 Up, 202 down)**	**WY Response in KO liver (16 Up, 5 down)**
**LncRNA gene ID**	**Exp. No. 338**	**Exp. No. 340**	**LncRNA gene ID**	**Exp. No. 338**	**Exp. No. 340**
nc_intra_c9_8171	Up	0	nc_as_c19_14940	Up	0
nc_inter_cx_15495	Up	0	nc_as_c19_14847	Up	0
nc_inter_cx_15394	Up	0	nc_as_c19_14782	Up	0
nc_inter_c9_8301	Up	0	nc_as_c18_14687	Up	0
nc_inter_c9_8175	Up	0	nc_as_c18_14615	Up	0
nc_inter_c9_8164	Up	0	nc_as_c18_14384	Up	0
nc_inter_c9_8158	Up	0	nc_as_c17_14041	Up	0
nc_inter_c9_8136	Up	0	nc_as_c17_13916	Up	0
nc_inter_c9_8122	Up	0	nc_as_c17_13877	Up	0
nc_inter_c9_8081	Up	0	nc_as_c17_13822	Up	0
nc_inter_c9_7993	Up	0	nc_as_c15_12907	Up	0
nc_inter_c9_7992	Up	0	nc_as_c15_12840	Up	0
nc_inter_c9_7991	Up	0	nc_as_c14_12017	Up	0
nc_inter_c9_7989	Up	0	nc_as_c13_11451	Up	Up
nc_inter_c9_7986	Up	0	nc_as_c13_11278	Up	0
nc_inter_c9_7908	Up	0	nc_as_c12_10896	Up	0
nc_inter_c9_7885	Up	0	nc_as_c10_9275	Up	0
nc_inter_c9_7791	Up	0	nc_as_c10_9064	Up	0
nc_inter_c8_7654	Up	0	nc_intra_c5_4738	Down	0
nc_inter_c8_7626	Up	0	nc_intra_c12_10888	Down	0
nc_inter_c8_7612	Up	0	nc_intra_c12_10886	Down	0
nc_inter_c8_7518	Up	0	nc_intra_c12_10870	Down	0
nc_inter_c8_7511	Up	0	nc_intra_c12_10859	Down	0
nc_inter_c8_7420	Up	0	nc_intra_c12_10856	Down	0
nc_inter_c8_7286	Up	0	nc_intra_c12_10851	Down	0
nc_inter_c8_7211	Up	0	nc_intra_c12_10508	Down	0
nc_inter_c8_7169	Up	0	nc_intra_c1_613	Down	0
nc_inter_c8_7105	Up	0	nc_intra_c1_611	Down	0
nc_inter_c8_6876	Up	0	nc_intra_c1_604	Down	0
nc_inter_c8_6744	Up	0	nc_inter_c9_8132	Down	0
nc_inter_c8_6743	Up	0	nc_inter_c9_7994	Down	0
nc_inter_c8_6742	Up	0	nc_inter_c9_7951	Down	0

nc_inter_c7_6558	Up	0	nc_inter_c9_7809	Down	0
nc_inter_c7_6402	Up	0	nc_inter_c8_7430	Down	0
nc_inter_c7_6308	Up	0	nc_inter_c8_7423	Down	0
nc_inter_c7_6195	Up	0	nc_inter_c8_7012	Down	0
nc_inter_c7_5993	Up	0	nc_inter_c8_6977	Down	0
nc_inter_c6_5845	Up	0	nc_inter_c8_6942	Down	0
nc_inter_c6_5816	Up	0	nc_inter_c8_6896	Down	Up
nc_inter_c6_5700	Up	0	nc_inter_c8_6881	Down	0
nc_inter_c6_5612	Up	0	nc_inter_c7_6679	Down	0
nc_inter_c6_5543	Up	0	nc_inter_c7_6220	Down	0
nc_inter_c6_5475	Up	0	nc_inter_c7_6113	Down	0
nc_inter_c6_5429	Up	0	nc_inter_c7_6079	Down	0
nc_inter_c6_4996	Up	0	nc_inter_c7_6073	Down	0
nc_inter_c6_4853	Up	0	nc_inter_c7_6022	Down	0
nc_inter_c5_4791	Up	0	nc_inter_c6_5650	Down	0
nc_inter_c5_4654	Up	0	nc_inter_c6_5556	Down	0
nc_inter_c5_4550	Up	0	nc_inter_c6_5551	Down	0
nc_inter_c5_4482	Up	0	nc_inter_c6_5402	Down	0
nc_inter_c5_4476	Up	0	nc_inter_c6_5376	Down	0
nc_inter_c5_4379	Up	0	nc_inter_c6_5368	Down	0
nc_inter_c5_4366	Up	0	nc_inter_c6_5322	Down	0
nc_inter_c5_4225	Up	0	nc_inter_c6_5316	Down	0
nc_inter_c5_4189	Up	0	nc_inter_c6_5118	Down	0
nc_inter_c5_4065	Up	0	nc_inter_c5_4819	Down	0
nc_inter_c5_4063	Up	0	nc_inter_c5_4784	Down	0
nc_inter_c4_3860	Up	0	nc_inter_c5_4777	Down	0
nc_inter_c4_3784	Up	0	nc_inter_c5_4639	Down	0
nc_inter_c4_3782	Up	0	nc_inter_c5_4578	Down	0
nc_inter_c4_3721	Up	0	nc_inter_c5_4343	Down	0
nc_inter_c4_3651	Up	0	nc_inter_c5_4338	Down	0
nc_inter_c4_3618	Up	0	nc_inter_c5_4069	Down	0
nc_inter_c4_3574	Up	0	nc_inter_c5_4066	Down	0
nc_inter_c4_3562	Up	0	nc_inter_c5_3988	Down	0
nc_inter_c4_3525	Up	0	nc_inter_c4_3731	Down	0
nc_inter_c4_3380	Up	0	nc_inter_c4_3468	Down	0
nc_inter_c4_3203	Up	0	nc_inter_c4_3295	Down	0
nc_inter_c4_3142	Up	0	nc_inter_c4_3294	Down	0
nc_inter_c4_3047	Up	0	nc_inter_c4_3282	Down	0
nc_inter_c3_2990	Up	0	nc_inter_c4_3228	Down	0
nc_inter_c3_2791	Up	0	nc_inter_c4_3079	Down	0
nc_inter_c3_2790	Up	0	nc_inter_c3_2887	Down	0
nc_inter_c3_2789	Up	0	nc_inter_c3_2798	Down	0
nc_inter_c3_2783	Up	0	nc_inter_c3_2779	Down	0
nc_inter_c3_2335	Up	0	nc_inter_c3_2676	Down	0

nc_inter_c3_2334	Up	0	nc_inter_c3_2663	Down	0
nc_inter_c3_2331	Up	0	nc_inter_c3_2639	Down	0
nc_inter_c3_2207	Up	0	nc_inter_c3_2504	Down	0
nc_inter_c2_2086	Up	0	nc_inter_c3_2431	Down	0
nc_inter_c2_2085	Up	0	nc_inter_c3_2169	Down	0
nc_inter_c2_2017	Up	Up	nc_inter_c3_2168	Down	0
nc_inter_c2_2016	Up	Up	nc_inter_c3_2167	Down	0
nc_inter_c2_2015	Up	0	nc_inter_c2_2066	Down	0
nc_inter_c2_2010	Up	0	nc_inter_c2_1990	Down	0
nc_inter_c2_1986	Up	0	nc_inter_c2_1889	Down	0
nc_inter_c2_1822	Up	0	nc_inter_c2_1887	Down	0
nc_inter_c2_1760	Up	0	nc_inter_c2_1830	Down	0
nc_inter_c2_1594	Up	0	nc_inter_c2_1782	Down	0
nc_inter_c2_1570	Up	0	nc_inter_c2_1302	Down	0
nc_inter_c2_1549	Up	0	nc_inter_c2_1236	Down	0
nc_inter_c2_1548	Up	0	nc_inter_c2_1235	Down	0
nc_inter_c2_1481	Up	0	nc_inter_c2_1233	Down	0
nc_inter_c2_1289	Up	0	nc_inter_c2_1232	Down	0
nc_inter_c2_1211	Up	0	nc_inter_c2_1157	Down	0
nc_inter_c19_15186	Up	0	nc_inter_c2_1156	Down	0
nc_inter_c19_15132	Up	0	nc_inter_c19_15097	Down	0
nc_inter_c19_15080	Up	0	nc_inter_c19_14990	Down	0
nc_inter_c19_15058	Up	0	nc_inter_c19_14965	Down	0
nc_inter_c19_15025	Up	0	nc_inter_c19_14954	Down	0
nc_inter_c19_14999	Up	0	nc_inter_c19_14803	Down	0
nc_inter_c19_14947	Up	0	nc_inter_c18_14678	Down	0
nc_inter_c19_14908	Up	0	nc_inter_c18_14525	Down	0
nc_inter_c19_14822	Up	0	nc_inter_c18_14341	Down	0
nc_inter_c19_14746	Up	0	nc_inter_c17_14189	Down	0
nc_inter_c18_14683	Up	0	nc_inter_c17_14182	Down	0
nc_inter_c18_14650	Up	0	nc_inter_c17_13938	Down	0
nc_inter_c18_14649	Up	0	nc_inter_c17_13843	Down	0
nc_inter_c18_14647	Up	0	nc_inter_c17_13742	Down	0
nc_inter_c18_14646	Up	0	nc_inter_c17_13576	Down	0
nc_inter_c18_14446	Up	0	nc_inter_c16_13433	Down	0
nc_inter_c18_14376	Up	0	nc_inter_c16_13348	Down	0
nc_inter_c17_14163	Up	0	nc_inter_c16_13316	Down	0
nc_inter_c17_14140	Up	0	nc_inter_c16_13177	Down	0
nc_inter_c17_14002	Up	0	nc_inter_c16_13176	Down	0
nc_inter_c17_13918	Up	0	nc_inter_c16_13173	Down	0
nc_inter_c17_13842	Up	0	nc_inter_c16_13171	Down	0
nc_inter_c17_13841	Up	0	nc_inter_c16_13170	Down	0
nc_inter_c17_13787	Up	0	nc_inter_c16_13161	Down	0
nc_inter_c17_13783	Up	0	nc_inter_c15_12319	Down	0

nc_inter_c17_13749	Up	0	nc_inter_c14_12304	Down	0
nc_inter_c17_13666	Up	0	nc_inter_c14_11949	Down	0
nc_inter_c16_13525	Up	0	nc_inter_c13_11768	Down	0
nc_inter_c16_13491	Up	0	nc_inter_c13_11670	Down	Up
nc_inter_c16_13488	Up	0	nc_inter_c13_11669	Down	0
nc_inter_c16_13475	Up	0	nc_inter_c13_11385	Down	Up
nc_inter_c16_13349	Up	0	nc_inter_c13_11227	Down	0
nc_inter_c16_13232	Up	0	nc_inter_c13_11212	Down	0
nc_inter_c16_13225	Up	0	nc_inter_c13_11104	Down	0
nc_inter_c16_13190	Up	0	nc_inter_c13_11046	Down	Up
nc_inter_c16_13081	Up	0	nc_inter_c12_10922	Down	Down
nc_inter_c16_13049	Up	0	nc_inter_c12_10910	Down	0
nc_inter_c15_12875	Up	0	nc_inter_c12_10717	Down	0
nc_inter_c15_12872	Up	0	nc_inter_c12_10628	Down	0
nc_inter_c15_12868	Up	0	nc_inter_c12_10595	Down	0
nc_inter_c15_12846	Up	0	nc_inter_c12_10454	Down	0
nc_inter_c15_12836	Up	0	nc_inter_c11_9925	Down	0
nc_inter_c15_12835	Up	0	nc_inter_c11_9856	Down	0
nc_inter_c15_12834	Up	Up	nc_inter_c11_9511	Down	0
nc_inter_c15_12833	Up	0	nc_inter_c11_9434	Down	0
nc_inter_c15_12642	Up	0	nc_inter_c11_10078	Down	0
nc_inter_c15_12606	Up	0	nc_inter_c11_10021	Down	0
nc_inter_c15_12538	Up	0	nc_inter_c10_9394	Down	0
nc_inter_c15_12530	Up	0	nc_inter_c10_9264	Down	0
nc_inter_c15_12529	Up	0	nc_inter_c10_9210	Down	0
nc_inter_c15_12514	Up	0	nc_inter_c10_8829	Down	0
nc_inter_c15_12451	Up	0	nc_inter_c10_8794	Down	0
nc_inter_c15_12365	Up	0	nc_inter_c10_8746	Down	0
nc_inter_c14_12199	Up	0	nc_inter_c10_8471	Down	Down
nc_inter_c14_12198	Up	0	nc_inter_c1_838	Down	0
nc_inter_c14_12188	Up	0	nc_inter_c1_630	Down	0
nc_inter_c14_12058	Up	0	nc_inter_c1_610	Down	0
nc_inter_c14_11978	Up	0	nc_inter_c1_602	Down	0
nc_inter_c14_11945	Up	0	nc_inter_c1_591	Down	0
nc_inter_c14_11942	Up	0	nc_inter_c1_543	Down	0
nc_inter_c13_11657	Up	0	nc_inter_c1_425	Down	0
nc_inter_c13_11628	Up	0	nc_inter_c1_406	Down	0
nc_inter_c13_11552	Up	0	nc_inter_c1_278	Down	0
nc_inter_c13_11399	Up	0	nc_inter_c1_235	Down	0
nc_inter_c13_11376	Up	0	nc_inter_c1_1035	Down	0
nc_inter_c13_11292	Up	0	nc_as_c9_8401	Down	0
nc_inter_c13_11254	Up	0	nc_as_c9_7825	Down	0
nc_inter_c13_11249	Up	0	nc_as_c9_7767	Down	0
nc_inter_c13_11216	Up	0	nc_as_c8_7528	Down	0

nc_inter_c13_11165	Up	0	nc_as_c8_7521	Down	0
nc_inter_c13_11049	Up	0	nc_as_c7_6643	Down	0
nc_inter_c12_10942	Up	0	nc_as_c7_6546	Down	0
nc_inter_c12_10672	Up	Down	nc_as_c7_6302	Down	0
nc_inter_c12_10286	Up	0	nc_as_c7_6300	Down	0
nc_inter_c12_10240	Up	0	nc_as_c7_6166	Down	0
nc_inter_c11_9993	Up	0	nc_as_c7_6065	Down	0
nc_inter_c11_9992	Up	0	nc_as_c7_5950	Down	0
nc_inter_c11_9965	Up	0	nc_as_c7_5911	Down	0
nc_inter_c11_9716	Up	0	nc_as_c6_5124	Down	0
nc_inter_c11_9626	Up	0	nc_as_c5_4370	Down	0
nc_inter_c11_9429	Up	0	nc_as_c4_3452	Down	0
nc_inter_c11_10143	Up	0	nc_as_c4_3300	Down	0
nc_inter_c11_10091	Up	0	nc_as_c4_3298	Down	0
nc_inter_c11_10029	Up	0	nc_as_c4_3297	Down	0
nc_inter_c10_9418	Up	0	nc_as_c4_3207	Down	0
nc_inter_c10_9313	Up	0	nc_as_c3_2936	Down	0
nc_inter_c10_9254	Up	0	nc_as_c3_2800	Down	0
nc_inter_c10_9251	Up	0	nc_as_c3_2485	Down	0
nc_inter_c10_9236	Up	0	nc_as_c2_1652	Down	0
nc_inter_c10_9177	Up	0	nc_as_c2_1560	Down	0
nc_inter_c10_9138	Up	0	nc_as_c2_1457	Down	0
nc_inter_c10_9059	Up	0	nc_as_c2_1343	Down	0
nc_inter_c10_8950	Up	0	nc_as_c2_1133	Down	0
nc_inter_c10_8868	Up	0	nc_as_c19_15151	Down	0
nc_inter_c10_8867	Up	0	nc_as_c19_15130	Down	0
nc_inter_c10_8866	Up	0	nc_as_c19_14977	Down	0
nc_inter_c10_8770	Up	0	nc_as_c19_14968	Down	0
nc_inter_c1_941	Up	0	nc_as_c19_14916	Down	0
nc_inter_c1_899	Up	0	nc_as_c18_14443	Down	0
nc_inter_c1_260	Up	0	nc_as_c18_14332	Down	0
nc_inter_c1_259	Up	0	nc_as_c17_14065	Down	0
nc_inter_c1_238	Up	0	nc_as_c17_13901	Down	0
nc_inter_c1_118	Up	0	nc_as_c16_13310	Down	0
nc_inter_c1_115	Up	0	nc_as_c16_13273	Down	0
nc_inter_c1_1032	Up	0	nc_as_c16_13146	Down	0
nc_as_c9_8393	Up	0	nc_as_c16_13145	Down	0
nc_as_c8_7281	Up	0	nc_as_c15_12340	Down	0
nc_as_c8_7185	Up	0	nc_as_c14_12150	Down	0
nc_as_c8_7071	Up	0	nc_as_c13_11596	Down	0
nc_as_c7_6542	Up	0	nc_as_c13_11563	Down	0
nc_as_c7_6493	Up	0	nc_as_c13_11185	Down	0
nc_as_c7_6192	Up	0	nc_as_c13_11127	Down	0
nc_as_c7_6021	Up	0	nc_as_c12_10884	Down	0

nc_as_c7_6009	Up	0	nc_as_c12_10850	Down	0
nc_as_c6_5250	Up	0	nc_as_c11_9684	Down	0
nc_as_c6_4824	Up	0	nc_as_c11_10149	Down	0
nc_as_c5_4655	Up	0	nc_as_c11_10122	Down	0
nc_as_c5_4325	Up	0	nc_as_c10_8962	Down	0
nc_as_c5_4085	Up	0	nc_as_c10_8848	Down	0
nc_as_c4_3435	Up	0	nc_as_c10_8847	Down	0
nc_as_c3_2781	Up	0	nc_as_c10_8460	Down	Up
nc_as_c3_2732	Up	0	nc_as_c1_798	Down	0
nc_as_c3_2620	Up	0	nc_as_c1_762	Down	0
nc_as_c2_2065	Up	0	nc_as_c1_504	Down	0
nc_as_c2_1724	Up	0	nc_as_c1_264	Down	0
nc_as_c2_1665	Up	0	nc_intra_c12_10883	0	Up
nc_as_c2_1309	Up	0	nc_inter_c9_8350	0	Down
nc_as_c2_1240	Up	0	nc_inter_c8_7393	0	Down
nc_as_c2_1101	Up	0	nc_inter_c8_6943	0	Up
nc_as_c19_15090	Up	0	nc_inter_c15_12684	0	Up
nc_as_c19_15060	Up	0	nc_inter_c13_11523	0	Up
nc_as_c19_14973	Up	0	nc_inter_c11_9650	0	Up
nc_as_c19_14966	Up	0	nc_inter_c11_9631	0	Up
			nc_as_c11_9447	0	Up
