# Supplementary material for: Long non-coding RNA Gm15441 attenuates hepatic inflammasome activation in response to PPARA agonism and fasting
Source: Nat Commun. 2020 Nov 17;11:5847. doi: 10.1038/s41467-020-19554-7 (PMC7673042; doi:10.1038/s41467-020-19554-7)
Supplement: Supplementary file 5 — Supplemental Data 3 [file 41467_2020_19554_MOESM5_ESM.pdf]

Supplementary Data 3. LncRNAs responsive to activators of PPARα, CAR, and PXR in mouse liver.

Groups #1, #2: lncRNAs that are consistently induced, or consistently repressed, by all 3 nuclear receptors, as indicated; Groups #3, #4: lncRNAs consistently induced or repressed by PPARα and by CAR, but not by PXR; Groups #5, #6: lncRNAs consistently induced or repressed by PPARα and by PXR, but not by CAR; Groups #7, #8: lncRNAs induced or repressed by PPARα but showing the opposite responses to activators of CAR, PXR, or both receptors. See Melia et al, 2019 for genomic coordinates and gene and isoform structures of lncRNAs. 'Inter', intergenic lncRNA; 'as', lncRNA that is anti-sense to a protein-coding gene; 'intra', lncRNA that is intragenic to, and transcribed from the same strand as a protein-coding gene but whose exons do not overlap those of the protein-coding gene.

LncRNA gene ID	PPARα response	CAR response (male)	PXR response	Nuclear receptor response group #
nc_inter_c15_12835	Up	Up	Up	1
nc_inter_c15_12836	Up	Up	Up	1
nc_inter_c19_14947	Up	Up	Up	1
nc_as_c5_4655	Up	Up	Up	1
nc_inter_c16_13225	Up	Up	Up	1
nc_inter_c5_4654	Up	Up	Up	1
nc_inter_c9_8301	Up	Up	Up	1
nc_inter_c4_3618	Up	Up (F-only)	Up	1
nc_inter_c12_10910	Down	Down (F-only)	Down	2
nc_inter_c16_13170	Down	Down (F-only)	Down	2
nc_inter_c16_13177	Down	Down (F-only)	Down	2
nc_inter_c7_6220	Down	Down (F-only)	Down	2
nc_as_c2_1343	Down	Down (F-only)	Down	2
nc_inter_c7_6113	Down	Down (F-only)	Down	2
nc_inter_c5_4777	Down	Down (F-only)	Down	2
nc_inter_c6_5551	Down	Down (F-only)	Down	2
nc_inter_c6_5316	Down	Down	Down	2
nc_as_c7_6065	Down	Down	Down	2
nc_as_c19_14977	Down	Down	Down	2
nc_as_c9_8393	Up	Up	Down	3
nc_inter_c13_11254	Up	Up	Down	3
nc_inter_c8_7511	Up	Up	Down	3
nc_as_c2_1101	Up	Up	Down	3
nc_inter_c19_14999	Up	Up		3
nc_inter_c9_7791	Up	Up		3
nc_as_c12_10896	Up	Up		3

nc_inter_c15_12606	Up	Up		3
nc_inter_c6_4853	Up	Up		3
nc_as_c5_4325	Up	Up (F-only)	Down	3
nc_inter_c10_9313	Up	Up (F-only)	Down	3
nc_as_c7_6192	Up	Up (F-only)		3
nc_as_c8_7071	Up	Up (F-only)		3
nc_inter_c10_9254	Up	Up (F-only)		3
nc_inter_c14_12058	Up	Up (F-only)		3
nc_inter_c16_13190	Up	Up (F-only)		3
nc_inter_c16_13349	Up	Up (F-only)		3
nc_inter_c17_13841	Up	Up (F-only)		3
nc_inter_c17_13842	Up	Up (F-only)		3
nc_inter_c4_3142	Up	Up (F-only)		3
nc_inter_c8_7169	Up	Up (F-only)		3
nc_inter_c8_7612	Up	Up (F-only)		3
nc_inter_c12_10672	Up	Up (F-only)		3
nc_inter_c7_6073	Down	Down (F-only)	Up	4
nc_as_c12_10884	Down	Down	Up	4
nc_inter_c9_7809	Down	Down	Up	4
nc_inter_c13_11385	Down	Down	Up	4
nc_inter_c11_9925	Down	Down (F-only)		4
nc_inter_c16_13176	Down	Down (F-only)		4
nc_inter_c3_2504	Down	Down (F-only)		4
nc_inter_c4_3468	Down	Down (F-only)		4
nc_inter_c8_7423	Down	Down (F-only)		4
nc_inter_c8_7430	Down	Down (F-only)		4
nc_inter_c10_9394	Down	Down (F-only)		4
nc_inter_c11_10078	Down	Down (F-only)		4
nc_inter_c15_12319	Down	Down (F-only)		4
nc_inter_c2_1990	Down	Down (F-only)		4
nc_inter_c5_3988	Down	Down (F-only)		4
nc_inter_c9_7994	Down	Down (F-only)		4
nc_as_c19_15130	Down	Down (F-only)		4
nc_inter_c16_13173	Down	Down		4
nc_inter_c8_6942	Down	Down		4
nc_as_c3_2800	Down	Down		4
nc_inter_c5_4578	Down	Down		4
nc_as_c9_7767	Down	Down		4
nc_inter_c11_9965	Up	Down (F-only)	Up	5
nc_inter_c11_10091	Up		Up	5
nc_inter_c15_12833	Up		Up	5
nc_inter_c17_13787	Up		Up	5
nc_inter_c19_15058	Up		Up	5

nc_inter_c2_2015	Up		Up	5
nc_inter_c2_2085	Up		Up	5
nc_inter_c3_2207	Up		Up	5
nc_inter_c8_6742	Up		Up	5
nc_inter_c8_6743	Up		Up	5
nc_as_c10_8848	Down		Down	6
nc_as_c11_10149	Down		Down	6
nc_as_c2_1652	Down		Down	6
nc_as_c5_4370	Down		Down	6
nc_inter_c1_591	Down		Down	6
nc_inter_c10_8829	Down		Down	6
nc_inter_c13_11212	Down		Down	6
nc_inter_c14_11949	Down		Down	6
nc_inter_c16_13171	Down		Down	6
nc_inter_c17_13938	Down		Down	6
nc_inter_c19_14990	Down		Down	6
nc_inter_c2_1302	Down		Down	6
nc_inter_c2_1782	Down		Down	6
nc_inter_c3_2798	Down		Down	6
nc_inter_c4_3731	Down		Down	6
nc_inter_c5_4066	Down		Down	6
nc_inter_c5_4784	Down		Down	6
nc_inter_c6_5322	Down		Down	6
nc_inter_c7_6022	Down		Down	6
nc_inter_c8_7012	Down		Down	6
nc_inter_c9_8122	Up	Down (F-only)	Down	7
nc_inter_c10_9418	Up	Down (F-only)	Down	7
nc_inter_c2_1594	Up	Down (F-only)	Down	7
nc_inter_c9_7989	Up	Down (F-only)	Down	7
nc_inter_c14_11945	Up	Down (F-only)	Down	7
nc_inter_c9_7992	Up	Down (F-only)		7
nc_inter_c8_6744	Up	Down	Up	7
nc_inter_c14_12199	Up	Down	Down	7
nc_inter_c15_12514	Up	Down	Down	7
nc_inter_c4_3651	Up	Down	Down	7
nc_as_c17_14041	Up	Down		7
nc_as_c19_14782	Up	Down		7
nc_inter_c19_15132	Up	Down		7
nc_inter_c12_10942	Up	Down		7
nc_inter_c9_7993	Up	Down		7
nc_as_c3_2781	Up		Down	7
nc_as_c6_5250	Up		Down	7
nc_inter_c1_115	Up		Down	7

nc_inter_c1_238	Up		Down	7
nc_inter_c10_8950	Up		Down	7
nc_inter_c10_9138	Up		Down	7
nc_inter_c13_11216	Up		Down	7
nc_inter_c14_12198	Up		Down	7
nc_inter_c17_14163	Up		Down	7
nc_inter_c18_14650	Up		Down	7
nc_inter_c19_14822	Up		Down	7
nc_inter_c2_1481	Up		Down	7
nc_inter_c3_2790	Up		Down	7
nc_inter_c3_2791	Up		Down	7
nc_inter_c4_3380	Up		Down	7
nc_inter_c5_4065	Up		Down	7
nc_inter_c6_5429	Up		Down	7
nc_inter_c8_7105	Up		Down	7
nc_as_c10_8962	Down	Up (F-only)		8
nc_as_c8_7521	Down	Up (F-only)		8
nc_as_c8_7528	Down	Up (F-only)		8
nc_intra_c5_4738	Down	Up (F-only)		8
nc_inter_c10_9210	Down	Up	Up	8
nc_inter_c5_4338	Down	Up	Up	8
nc_inter_c8_6896	Down	Up	Up	8
nc_as_c10_8460	Down	Up	Up	8
nc_inter_c12_10454	Down	Up	Down	8
nc_as_c11_9684	Down	Up		8
nc_as_c16_13145	Down	Up		8
nc_inter_c1_630	Down	Up		8
nc_inter_c12_10628	Down	Up		8
nc_inter_c4_3294	Down	Up		8
nc_inter_c19_15097	Down	Up		8
nc_as_c13_11596	Down	Up		8
nc_as_c2_1457	Down	Up		8
nc_as_c9_8401	Down	Up		8
nc_inter_c3_2779	Down	Up		8
nc_inter_c6_5118	Down	Up		8
nc_intra_c1_604	Down	Up		8
nc_inter_c4_3282	Down	Up		8
nc_as_c7_6302	Down	Up		8
nc_inter_c2_1887	Down		Up	8
nc_inter_c3_2887	Down		Up	8
nc_inter_c4_3079	Down		Up	8
