# Supplementary material for: Long non-coding RNA Gm15441 attenuates hepatic inflammasome activation in response to PPARA agonism and fasting
Source: Nat Commun. 2020 Nov 17;11:5847. doi: 10.1038/s41467-020-19554-7 (PMC7673042; doi:10.1038/s41467-020-19554-7)
Supplement: Supplementary file 6 — Reporting Summary [file 41467_2020_19554_MOESM6_ESM.pdf]

nature research

Corresponding author(s): Frank J. Gonzalez

Last updated by author(s): September 16, 2020

Reporting Summary

Nature Research wishes to improve the reproducibility of the work that we publish. This form provides structure for consistency and transparency in reporting. For further information on Nature Research policies, see our Editorial Policies and the Editorial Policy Checklist.

Statistics

For all statistical analyses, confirm that the following items are present in the figure legend, table legend, main text, or Methods section.

n/a	Confirmed	
☐	☒	The exact sample size (*n*) for each experimental group/condition, given as a discrete number and unit of measurement
☐	☒	A statement on whether measurements were taken from distinct samples or whether the same sample was measured repeatedly
☐	☒	The statistical test(s) used AND whether they are one- or two-sided<br>*Only common tests should be described solely by name; describe more complex techniques in the Methods section.*
☒	☐	A description of all covariates tested
☐	☒	A description of any assumptions or corrections, such as tests of normality and adjustment for multiple comparisons
☐	☒	A full description of the statistical parameters including central tendency (e.g. means) or other basic estimates (e.g. regression coefficient) AND variation (e.g. standard deviation) or associated estimates of uncertainty (e.g. confidence intervals)
☐	☒	For null hypothesis testing, the test statistic (e.g. *F*, *t*, *r*) with confidence intervals, effect sizes, degrees of freedom and *P* value noted<br>*Give P values as exact values whenever suitable.*
☒	☐	For Bayesian analysis, information on the choice of priors and Markov chain Monte Carlo settings
☒	☐	For hierarchical and complex designs, identification of the appropriate level for tests and full reporting of outcomes
☒	☐	Estimates of effect sizes (e.g. Cohen's *d*, Pearson's *r*), indicating how they were calculated

Our web collection on statistics for biologists contains articles on many of the points above.

Software and code

Policy information about availability of computer code

Data collection	Microscope images were obtained with Keyence BZ-X700 series all-in-one microscope (BZ-X Viewer software, v1.0.0). CT values for qRT-PCR data were obtained with QuantStudio 7 Flex Real-Time PCR System (Thermo Fisher Scientific, v1.3). FISH data was obtained using Aperio Image Scope software (Leicabiosysytems, v12.4.3). TopHat2 (v2.1.1) was used to RNA-seq analysis. HTSeq (0.6.1p1) was used to obtain read counts for exon collapsed regions of RefSeq genes, and featureCounts (1.4.6-p5) was used to obtain read counts for exon collapsed regions of lncRNA genes. EdgeR was used to check Refseq and lncRNA gene significance. ChemiDoc MP system (bio-rad) and Image lab software (bio-rad, v6.0.1 build 34) were used to Western band detection. Glucometer (Bayer) was used to measure glucose level in blood.
Data analysis	Statistical analyses were performed using GraphPad Prism 7.0 (GraphPad Software), Protein densitometry analyses were performed using ImageJ (NIH, v1.52a), PPARA ChIP-seq and RNA-seq analysis were performed using CLC genome Workbench (Qiagen, v20.0.4), Integrated Genome Browser (Bioviz, v9.1.4) and NCBI Gene Expression Omnibus (NCBI).

For manuscripts utilizing custom algorithms or software that are central to the research but not yet described in published literature, software must be made available to editors and reviewers. We strongly encourage code deposition in a community repository (e.g. GitHub). See the Nature Research guidelines for submitting code & software for further information.

Data

Policy information about availability of data

All manuscripts must include a data availability statement. This statement should provide the following information, where applicable:

- Accession codes, unique identifiers, or web links for publicly available datasets
- A list of figures that have associated raw data
- A description of any restrictions on data availability

All data underlying the findings reported in this manuscript are provided as part of the article. PPARA ChIP-seq data was downloaded from NCBI Gene Expression

Omnibus (GSE61817, https://www.ncbi.nlm.nih.gov/geo/query/acc.cgi?acc=GSE61817). Raw and processed RNA-seq data are available at GEO (https://www.ncbi.nlm.nih.gov/gds) accession numbers GSE132385 (https://www.ncbi.nlm.nih.gov/geo/query/acc.cgi?acc=GSE132385) and GSE132386 (https://www.ncbi.nlm.nih.gov/geo/query/acc.cgi?acc=GSE132386).

Field-specific reporting

Please select the one below that is the best fit for your research. If you are not sure, read the appropriate sections before making your selection.

☒ Life sciences ☐ Behavioural & social sciences ☐ Ecological, evolutionary & environmental sciences

For a reference copy of the document with all sections, see nature.com/documents/nr-reporting-summary-flat.pdf

Life sciences study design

All studies must disclose on these points even when the disclosure is negative.

Sample size	The sample sizes were determined based on our previous studies using similar methodologies (Broker CN et al, PMID:30158201, 28082284; Li et al, PMID:28918936) and other publications in the same field. The sample sizes and statistical methods are described in figure legends and in the method section. For biochemical data and quantifications, 3-6 biological replicates were used in a majority of experiments; for imaging data, 3 biological replicates were used.
Data exclusions	No samples or animals were excluded from the study.
Replication	All experiments included in this study were done in biological replicates, as described in each figure legend for each experiment. All reported data was successfully reproduced in independent replicate experiments.
Randomization	C57BL/6 mice were ordered from the Jackson Lab. Each cage was randomly chosen to receive treatments. Animals were assigned to groups randomly. For knockout mice study, experiments were not allocated into random groups because relevant genotypes after genetic crossing were selected for experimentation.
Blinding	Blinding was not relevant to this study because all data from in vitro and in vivo were analyzed in the same way.

Reporting for specific materials, systems and methods

We require information from authors about some types of materials, experimental systems and methods used in many studies. Here, indicate whether each material, system or method listed is relevant to your study. If you are not sure if a list item applies to your research, read the appropriate section before selecting a response.

Materials & experimental systems

n/a	Involved in the study	
☐	☒	Antibodies
☐	☒	Eukaryotic cell lines
☒	☐	Palaeontology and archaeology
☐	☐	Animals and other organisms
☒	☐	Human research participants
☒	☐	Clinical data
☒	☐	Dual use research of concern

Methods

n/a	Involved in the study	
☒	☐	ChIP-seq
☒	☐	Flow cytometry
☒	☐	MRI-based neuroimaging

Antibodies

Antibodies used

CASP1 (Invitrogen, Cat:#14-9832-82, Clone:5B10, LOT:2171961)
IL1B (Cell Signaling, Cat:#12242, Clone:3A6, LOT:1)
ACTB (Cell Signaling, Cat:#8457L, Clone:D6A8, LOT:1)
TXNIP (Novus, Cat:NBP1-54578, Clone:JY2, LOT:A-4)
Anti-rabbit IgG HRP-linked antibody (Cell Signaling, Cat:7074S, LOT:29)
Anti-mouse IgG HRP-linked antibody (Cell Signaling, Cat:7076S, LOT:35)
Histone H3 (Cell Signaling, Cat:#4620, Clone:D2B12, Lot:1)
GFP (MBL, Cat:#598, LOT:076)
PPARα (Abcam, Cat:Ab24509)
Normal rabbit IgG (Cell Signaling, Cat:#2729S)
TUBA1B (Epitomics, Cat:#1878-1, Clone:EP1332Y)

Validation

All the antibodies used in this study were validated by providers and previous reports.
CASP1 antibody has been validated (Westerterp et al., 2017).
IL1B antibody has been validated (Song et al., 2017).

TXNIP antibody has been validated (Dotimas et al., 2016).
ACTB antibody has been validated (Sbiera et al., 2015).
Anti-rabbit IgG HRP-linked antibody has been validated (Boerner et al., 2013)
Anti-mouse IgG HRP-linked antibody has been validated (Peverelli et al., 2014)
Histone H3 has been validated (Ding et al., 2015)
GFP antibody has been validated (Arii J et al., 2018)
PPARα has been validated (Nikolaenko et al., 2014)
Normal rabbit IgG has been validated (Hall et al., 2017)
TUBA1B antibody has been validated (Wehde et al., 2018).

Eukaryotic cell lines

Policy information about cell lines

Cell line source(s)	Hepa-1 (CRL-1830), NIH3T3 (CRL-1658) and primary hepatocyte were used in this study. Hepa-1 and NIH3T3 lines were purchased from American Type Culture Collection (ATCC). Primary hepatocyte was harvested from C57BL/6 male mice liver.
Authentication	None of the cell lines used were authenticated.
Mycoplasma contamination	All cell lines tested negative for mycoplasma contamination
Commonly misidentified lines (See ICLAC register)	No commonly misidentified cell lines were used.

Animals and other organisms

Policy information about studies involving animals; ARRIVE guidelines recommended for reporting animal research

Laboratory animals	Male 8- to 12-week-old mice were used for all studies and all mouse strains were on the C57BL/6J background and maintained on a grain-based control diet (NIH-31). Mice were housed in light (12 hours light: 12 hours darkness cycle) and temperature-controlled rooms (humidity 40-60%) and were provided with water and pelleted chow ad libitum. For generation of Gm15441-null mice, SAGE Laboratories (Cambridge, UK) provided design and construction services for the CRISPR/Cas gene targeting technologies used to create a Gm15441-null mouse line. The targeting strategy results in the insertion of a floxed cassette containing a transcriptional stop repeat within the first intron of Gm15441 (NR_040409.1) (Supplementary Table 3). Presence of the cassette prevents Gm15441 expression. Crossing with a Cre mouse line removes the stop cassette and allows Gm15441 expression to proceed. Microinjection-ready sgRNA, Cas9 mRNA, and a plasmid donor with a floxed stop cassette were purchased from SAGE Laboratories. The sgRNA, Cas9 mRNA, and plasmid donor were then injected into C57BL/6J mouse embryos by the Transgenic Mouse Model Laboratory at the National Cancer Institute (Fredrick, MD) using the manufacturer's recommended protocol. Founder animals were genotyped using primer sets in Supplementary Table 4, and all modifications confirmed by targeted sequencing. Homozygous mice were then backcrossed ten times into the C57BL6 background bred out any off-target effects. All mouse studies were approved by the NCI Animal Care and Use Committee and performed in accordance with the Institute of Laboratory Animal Resources guidelines.
Wild animals	No such animals were used
Field-collected samples	No such samples were used
Ethics oversight	All animal experiments were performed in accordance with the Association for Assessment and Accreditation of Laboratory Animal Care international guidelines and approved by the National Cancer Institute Animal Care and Use Committee.

Note that full information on the approval of the study protocol must also be provided in the manuscript.
